# Supplementary material for: Pocket delipidation induced by membrane tension or modification leads to a structurally analogous mechanosensitive channel state
Source: Structure. 2022 Apr 7;30(4):608–622.e5. doi: 10.1016/j.str.2021.12.004 (PMC9033278; doi:10.1016/j.str.2021.12.004)
Supplement: Document S1. Figures S1–S10 and Tables S1 and S2 [file mmc1.pdf]

Structure, Volume *30*

Supplemental Information

Pocket delipidation induced by membrane tension or modification leads to a structurally analogous mechanosensitive channel state

Bolin Wang, Benjamin J. Lane, Charalampos Kapsalis, James R. Ault, Frank Sobott, Hassane El Mkami, Antonio N. Calabrese, Antreas C. Kalli, and Christos Pliotas

Supplementary Material for

Pocket delipidation induced by membrane tension or modification leads to a structurally analogous mechanosensitive channel state

Bolin Wang,[1,2†] Benjamin J. Lane,[1,2†] Charalampos Kapsalis,[3] James R. Ault,[1,4] Frank Sobott,[1,4] Hassane El Mkami,[5] Antonio N. Calabrese,[1,4] Antreas C. Kalli,[1,6] Christos Pliotas[1,2,3*]

[1]Astbury Centre for Structural Molecular Biology, University of Leeds, UK
[2]School of Biomedical Sciences, Faculty of Biological Sciences, University of Leeds, UK
[3]Biomedical Sciences Research Complex, School of Biology, University of St Andrews, UK
[4]School of Molecular and Cellular Biology, Faculty of Biological Sciences, University of Leeds, UK
[5]School of Physics and Astronomy, University of St Andrews, UK
[6]Leeds Institute of Cardiovascular and Metabolic Medicine, School of Medicine, University of Leeds, UK

[†]These authors contributed equally to this work

*Corresponding author: Christos Pliotas, c.pliotas@leeds.ac.uk

This file contains supplementary:

Figures with legends 1 to 10
Tables with legends 1 to 2

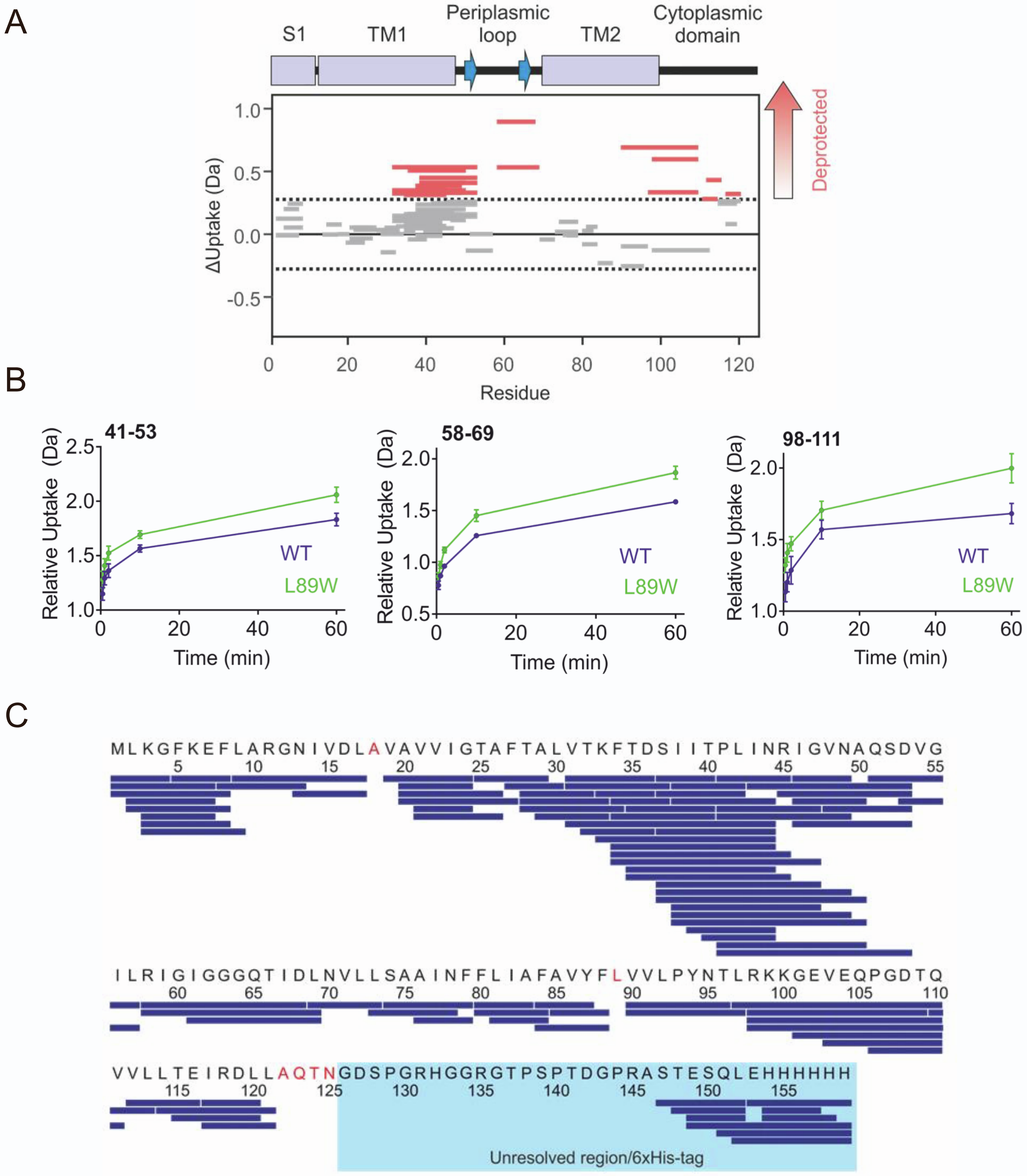

Figure S1. HDX-MS comparing WT and L89W MscL, Related to Figure 1. A. Wood's plots showing the summed differences in deuterium uptake in MscL over all five HDX timepoints, comparing wildtype MscL with L89W MscL (Wood's plots were generated using Deuteros (Lau et al., 2019). Peptides coloured in red, are deprotected from exchange in L89W MscL. No peptides were significantly protected from exchange in L89W MscL compared with wild type MscL. Peptides with no significant difference between conditions, determined using a 95% confidence interval (dotted line), are shown in grey. B. Example deuterium uptake curves for MscL WT (blue) L89W (green). The residue numbers in each peptide are indicated in the top left of each plot. C. Map showing peptides from MscL detected in the HDX-MS experiment (blue bars). Residues in red are not covered by any of the detected peptides. The region highlighted in light blue corresponds to the region of the protein that is not resolved in the x-ray structure and the 6xHis-tag.

A

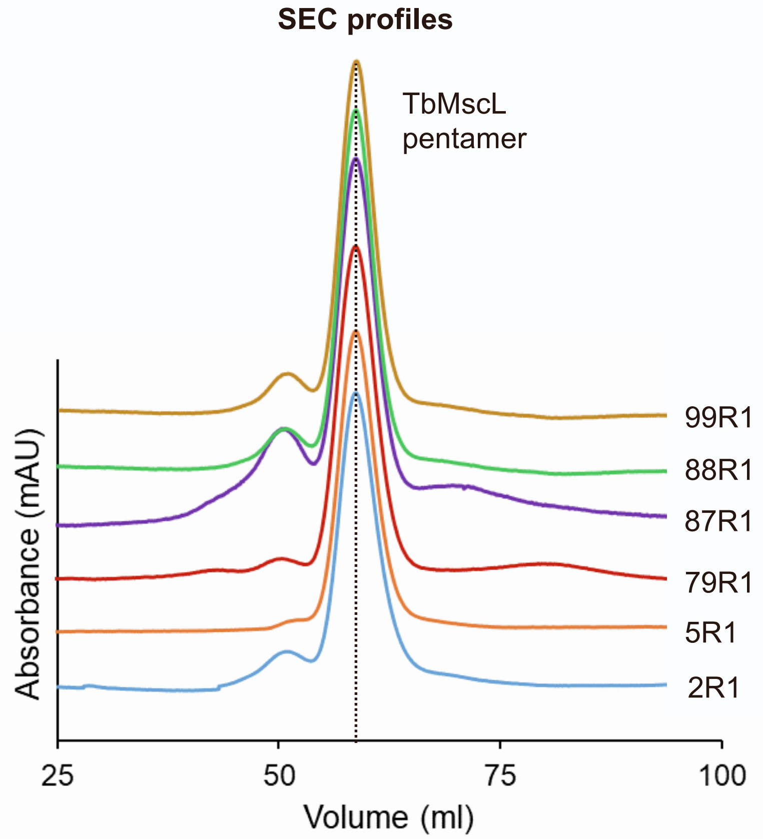

B

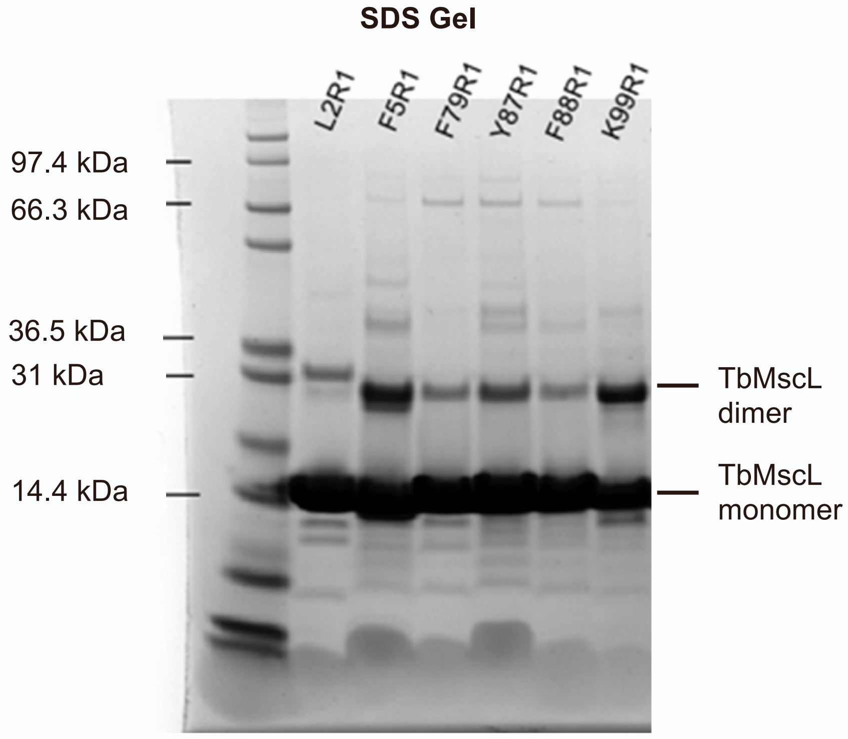

Figure S2. Purification of MscL Mutants, Related to Figure 2 and Figure 3. Sample characterisation and assessment of purified TbMscL proteins used for 3pESEEM and HDX-MS. A. Size exclusion chromatography (SEC) profiles of spin labelled TbMscL mutants representative of multiple protein domains. All purified mutants are monodisperse and in pentameric states in DDM solution, consistent with previous data (Kapsalis et al., 2019). B. SDS gel analysis of the respective mutants (SEC profile peaks). Two distinct bands appear for each mutant, which correspond to the monomeric and dimeric form(s) of the labelled proteins, under SDS denaturing conditions.

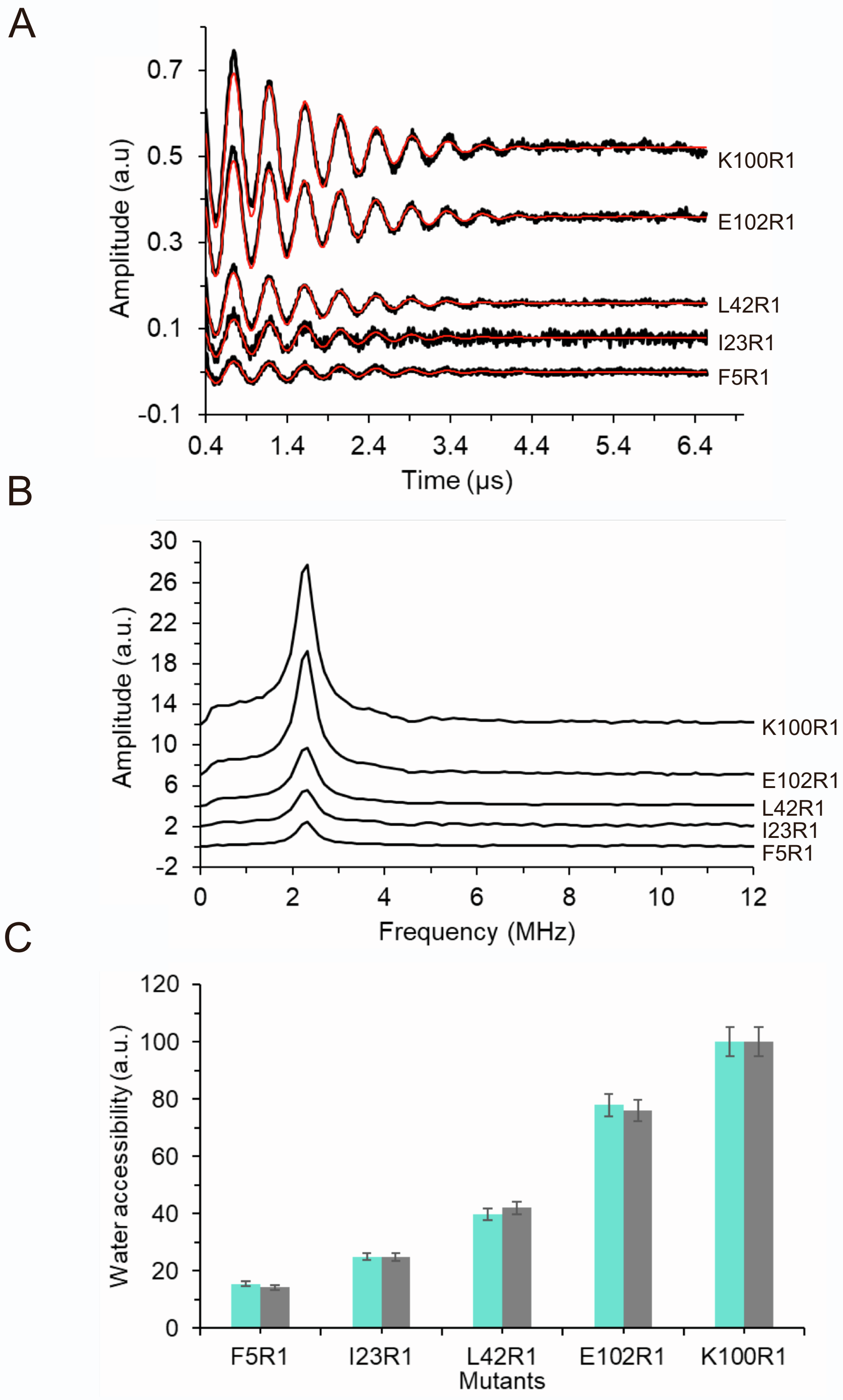

Figure S3. Comparison of Deuterium (solvent) accessibility obtained from 3pESEEM time-domain and frequency spectra, Related to Figure 2 and Figure 3. A Background-corrected time-domain 3pESEEM raw spectra (black traces) with fitting (red) of representative in respect of solvent exposure level and MscL domain coverage spin-labelled mutants. F5R1 is found on the S1, I23R1 and L42R1 on TM1, and K100R1 and E102R1 are at the interface between TM2 and the CHB. B Frequency domain spectra of 3pESEEM data of F5R1, I23R1, L42R1, K100R1, and E102R1. C Column bar charts representing solvent accessibility parameters obtained by two different analysis method approaches. For each sample, the cyan bars correspond to the solvent accessibility derived from the deuterium amplitudes in frequency domain 3pESEEM spectra and normalized to the highest accessibility corresponding to 100%. The grey bars correspond to the solvent accessibility determined from the fitting model to the time domain 3pESEEM spectra and normalized to the highest accessibility corresponding to 100%. More details on data processing in *Materials and Methods.*

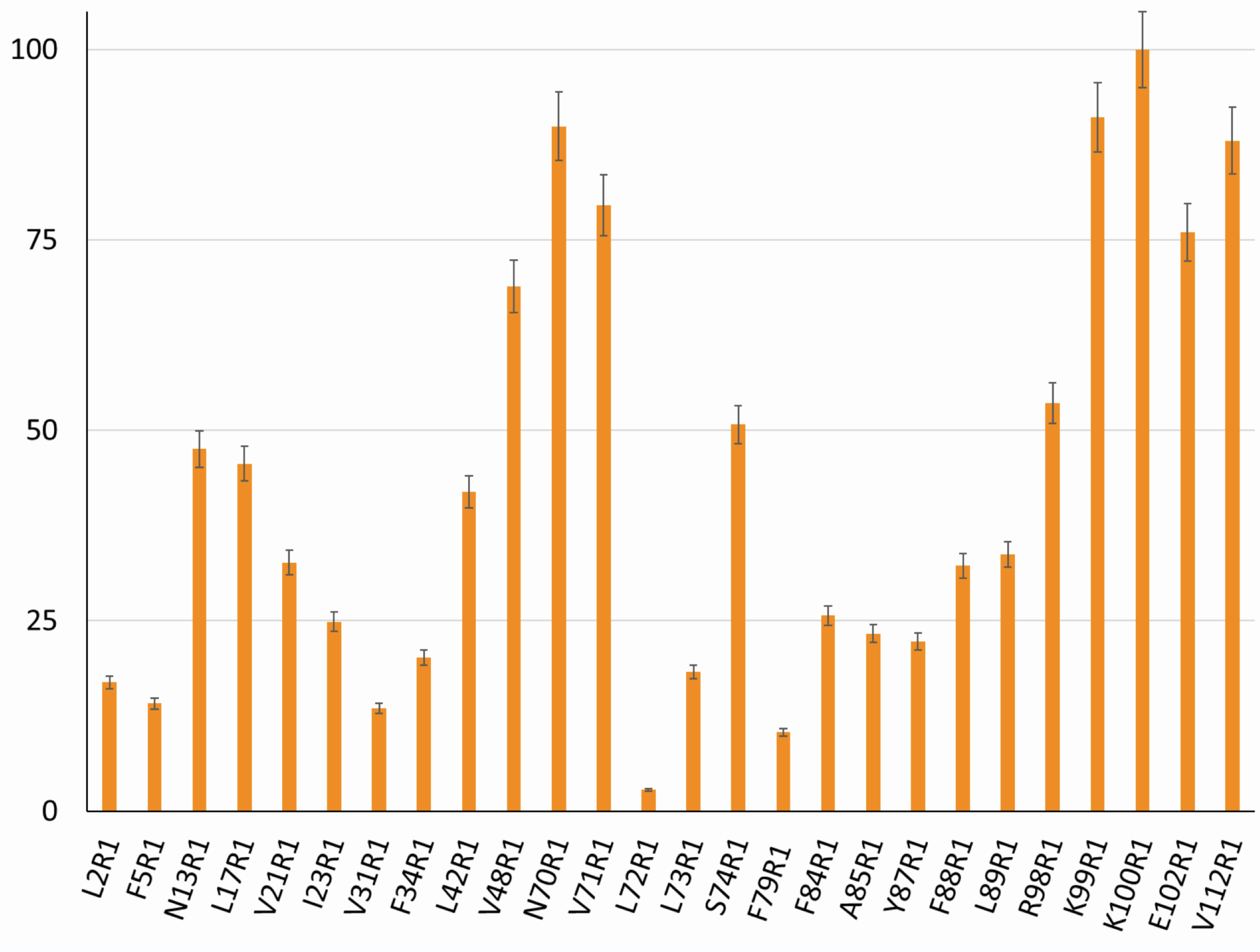

Figure S4. Solvent accessibility of spin-labelled residues, Related to Figure 2. The column bar charts represent the deuterium (or solvent) accessibility of each spin-labelled residue derived from the fitting of 3pESEEM time-domain traces, normalised on a scale between 0 and 100**.** Errors are calculated at 5% to compensate for fitting errors and differences in the relaxation times of different spin labelled residues.

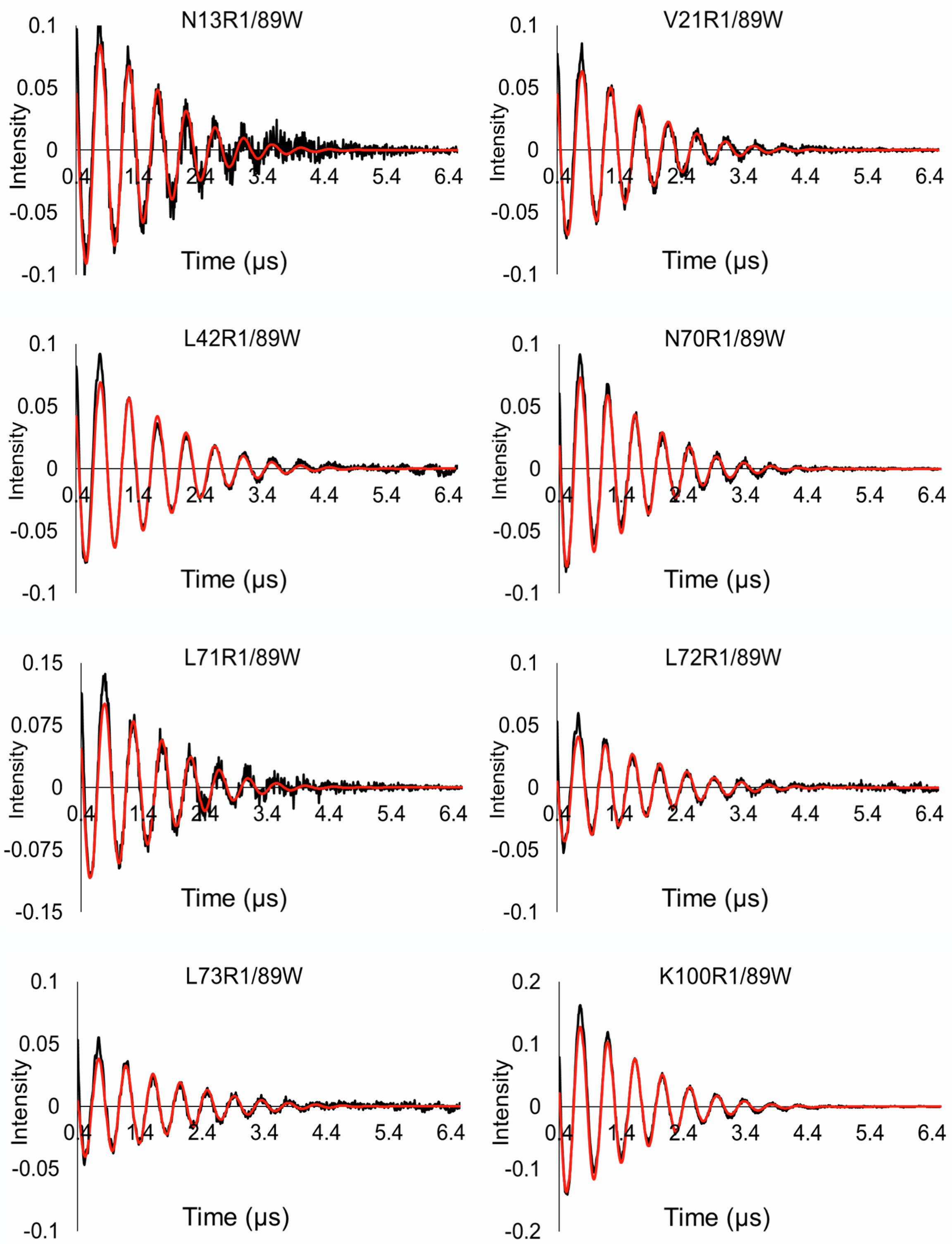

Figure S5. Time-domain 3pESEEM raw (black) and fitted (red) experimental spectra of the double TbMscL mutants N13R1/89W, V21R1/89W, L42R1/89W, N70R1/89W, L71R1/89W, L72R1/89W, L73R1/89W, and K100R1/89W, Related to Figure 3.

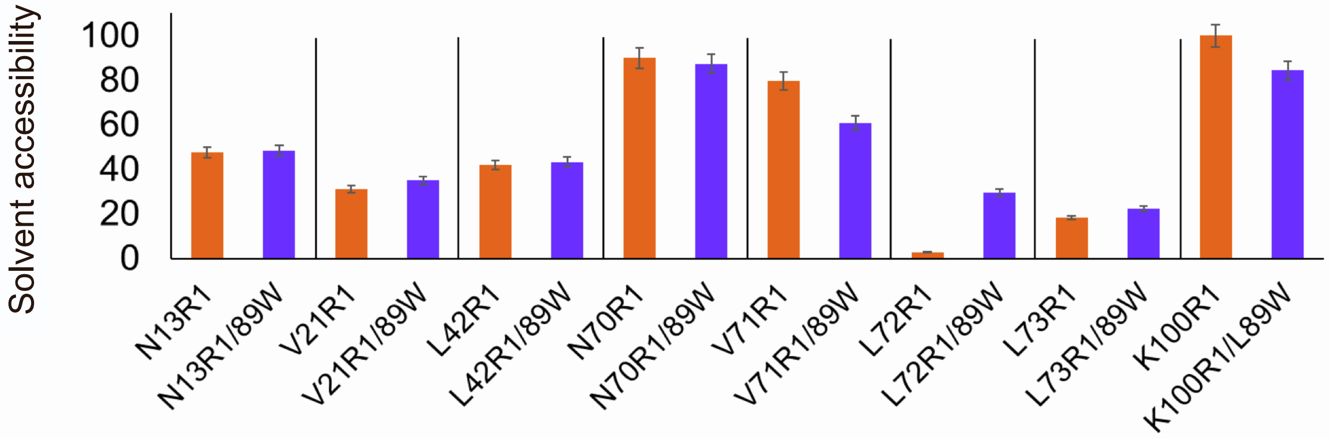

Figure S6. Column bar charts represent the Deuterium accessibility derived from the fitting of 3pESEEM time-domain traces, Related to Figure 3. Errors are calculated at 5% to compensate for the fitting error and differences in relaxation times of different spin labelled residues (*for more details see Methods*).

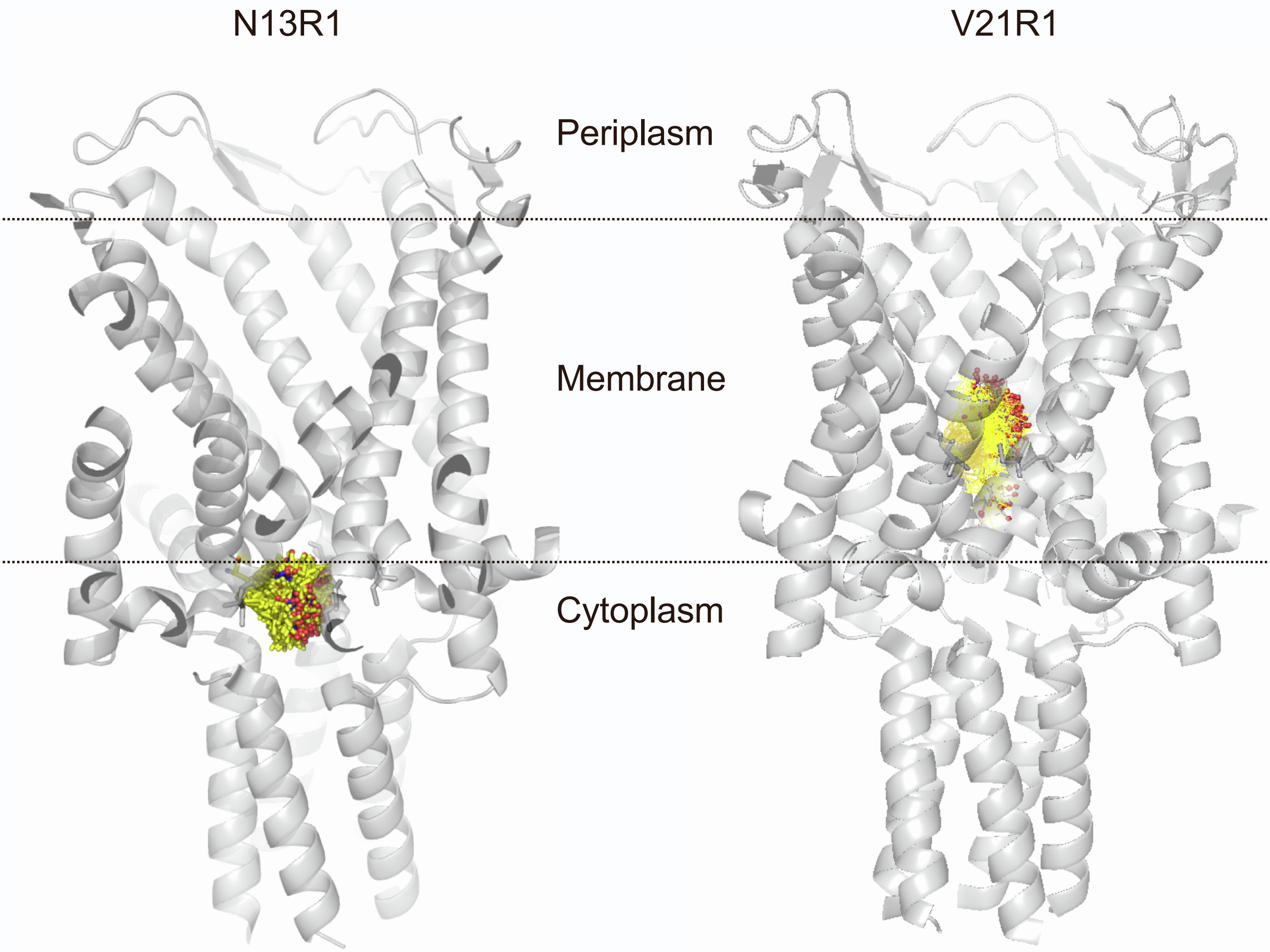

Figure S7. ***In sillico*** **spin labelling of TbMscL (PDB 2OAR, closed state) N13 and V21, Related to Figure 3.** N13R1's side chain is already solvent exposed in the closed state, due to pointing into the cytoplasm, thus unaffected in the expanded intermediate state.

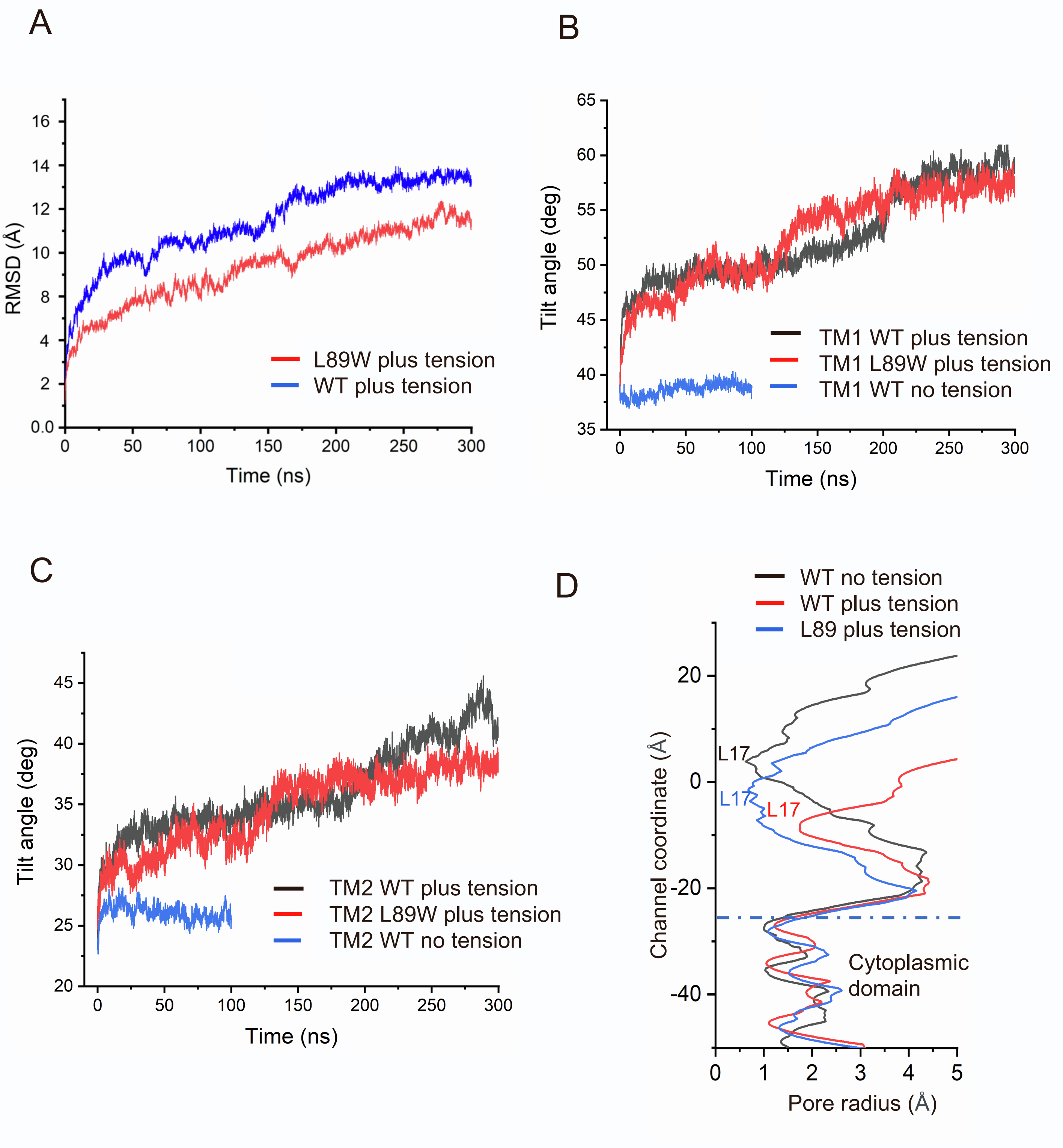

Figure S8. Comparison of WT and L89W MscL in MD simulations under no and applied membrane tension conditions, Related to Figure 4 and Figure 5. A. RMSD between WT and L89W TbMscL under applied to the x-y, parallel to the membrane plane bilayer tension. Tilting angle comparison between under tension and no tension and modified states of B. TM1 and C. TM2 with respect to the z-axis over time. D. MscL pore radius profiles analysis and comparisons using HOLE(Smart et al., 1993). Although L89W MscL undergoes similar major rearrangements to WT MscL under tension its pore remains significantly smaller and almost identical to WT closed MscL under no tension.

A

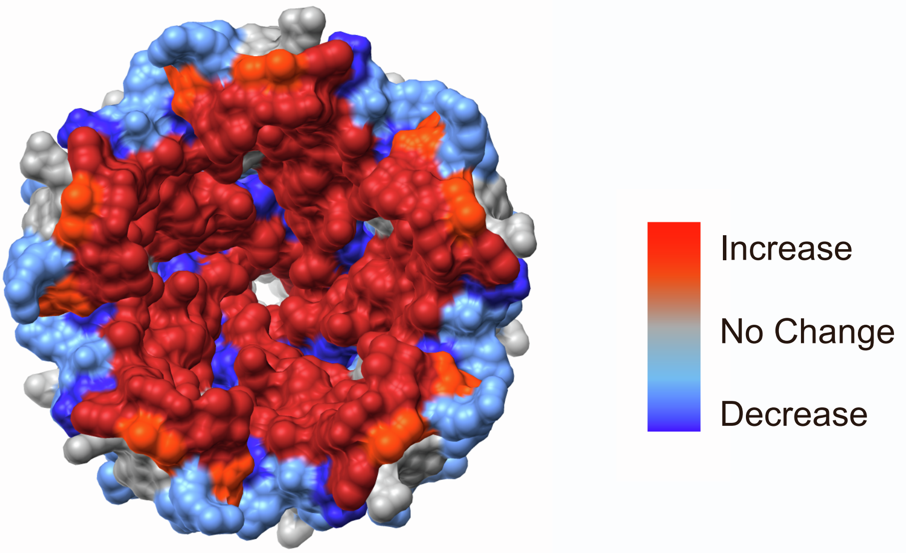

B

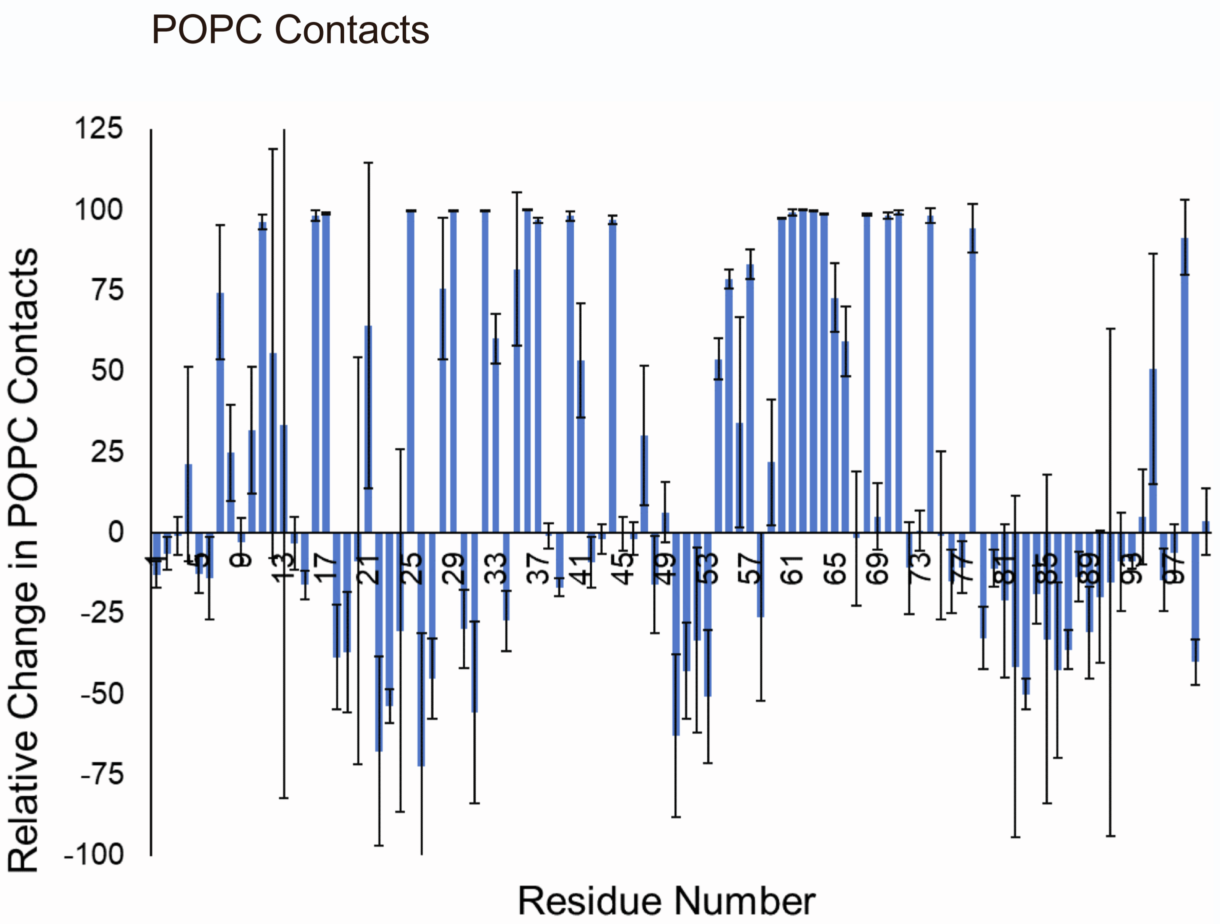

Figure S9 The effect of tension on lipid-residue contacts in MD simulations, Related to Figure 6. A. MscL top pore view showing relative changes in the number of lipid contacts following simulated tension application in the membrane during MD. The blue regions show decrease in lipid contacts, while the red regions show an increase in lipid contacts. B. relative difference between the expanded tension-activated state and the closed (no tension) state of POPC contacts with TbMscL residues.

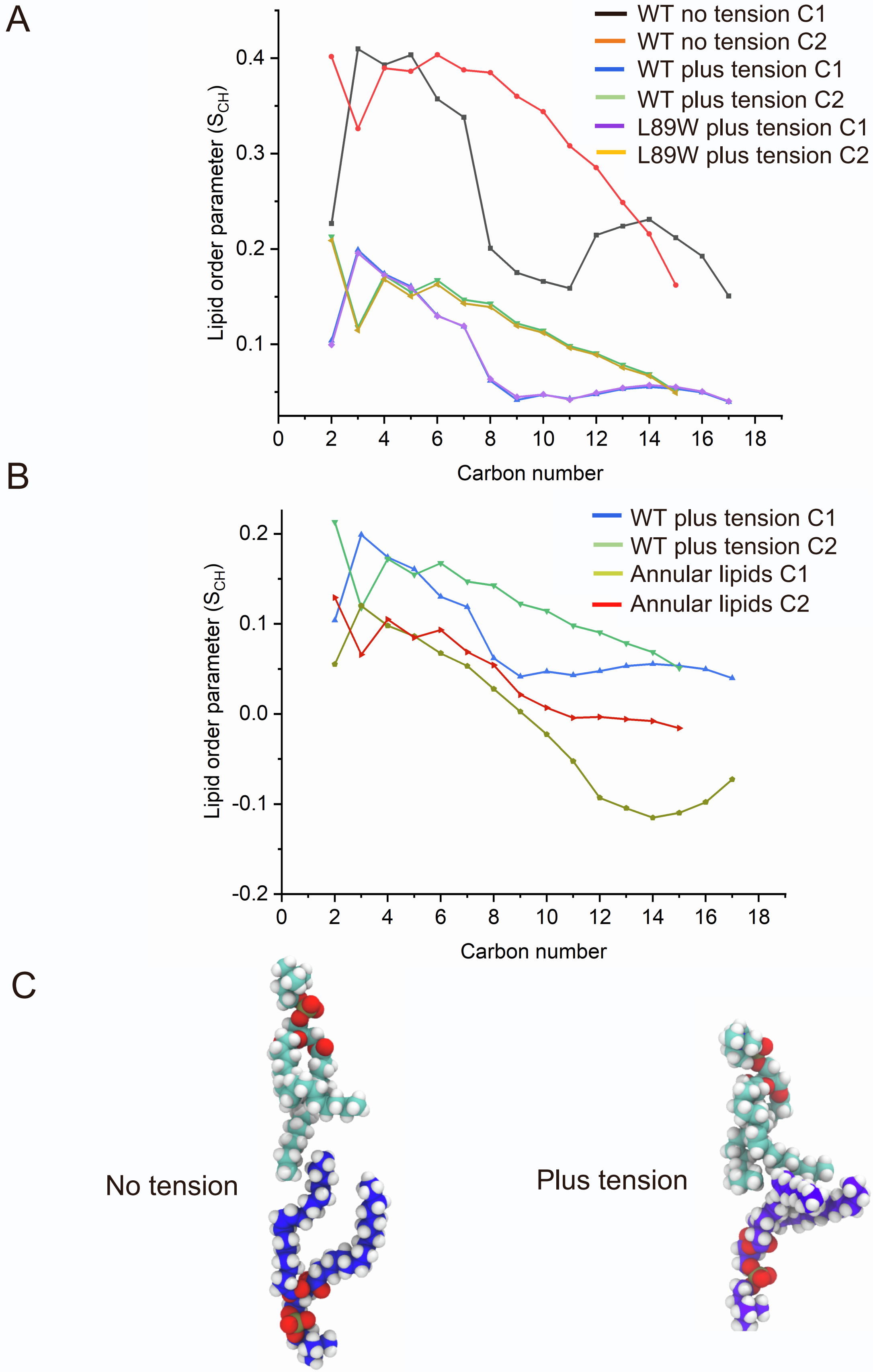

Figure S10. Comparison of the lipid order parameters, Related to Figure 6. A. Comparison for all the lipids included in the simulation of WT MscL with no tension, WT and L89W MscL under tension. Lipid chains are more horizontally oriented under tension compared to chains with no tension while in both L89W and WT MD simulations lipid chains adopt similar horizontal orientations. B. Comparison for all bilayer and annular lipids included in the WT MscL MD simulations under tension. Annular lipids are more "horizontally" oriented than bulk bilayer lipids. C. representative lipids in MD with and no tension. S_{ch} was calculated using the formula: $S_{ch} = ½ * (3 \cos^2θ – 1)$, where θ is the angle formed between the (C-1) and (C+1) vector and the membrane horizontal axis.

Table S1. HDX Data Summary Table, Related to Figure 1.
SD = standard deviation, CI = confidence interval

Data Set	Tb MscL	L89W MscL
**HDX reaction details**	50 mM potassium phosphate pH 7.4, 300 mM NaCl, 0.05% DDM	
**HDX time course (min)**	0.5, 1, 2, 10, 60	
**HDX control samples**	Maximally-labelled controls were not performed.	
**Back-exchange**	~ 30 %	
**# of Peptides**	101	101
**Sequence coverage**	83 %	83 %
**Average peptide length / Redundancy**	7.3 / 5.6	7.3 / 5.6
**Replicates (biological or technical)**	3 technical, 2 biological	3 technical, 2 biological
**Repeatability**	0.049 (average SD)	0.053 (average SD)
**Significant differences in HDX (delta HDX > X D)**	Reference	99% or 95 % CI in summed data using Deuteros

Table S2. Overall RMSD among structures generated in this study by MD (under membrane tension) and the expanded x-ray MscL structure (PDB 4Y7J), Related to Figure 4 and Figure 7.

	WT under tension (MD) (Å)	L89W under tension (MD) (Å)	Expanded x-ray structure (Å)
WT under tension (MD) (Å)	-	3.1	3.3
L89W under tension (MD) (Å)	3.1	-	3.4
