# Supplementary figures and images for: Pocket delipidation induced by membrane tension or modification leads to a structurally analogous mechanosensitive channel state
Source: Structure. 2022 Apr 7;30(4):608–622.e5. doi: 10.1016/j.str.2021.12.004 (PMC9033278; doi:10.1016/j.str.2021.12.004)

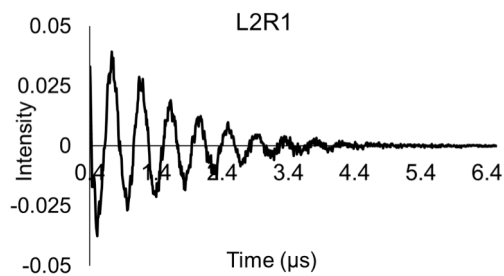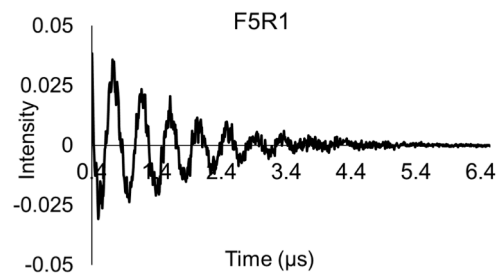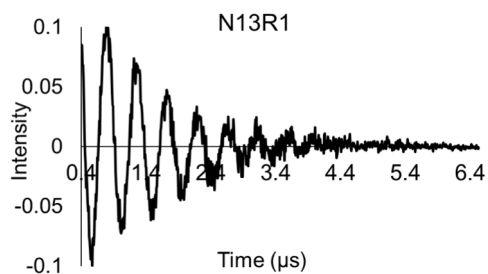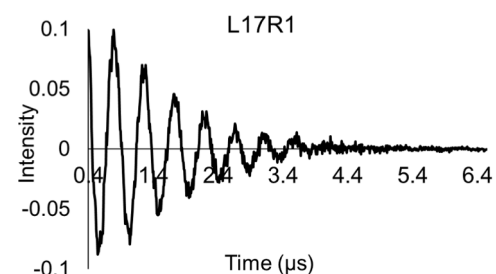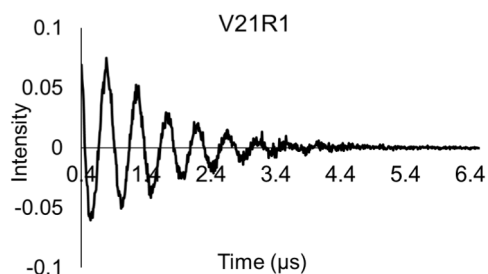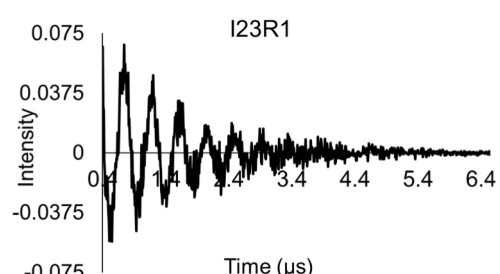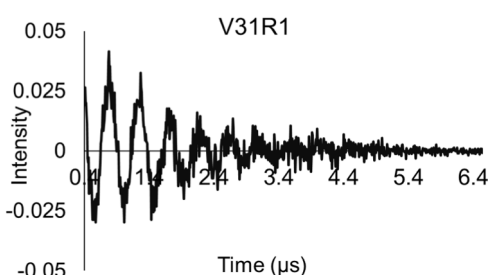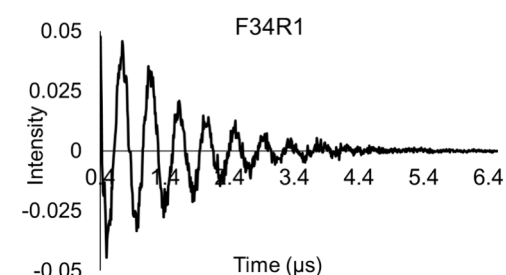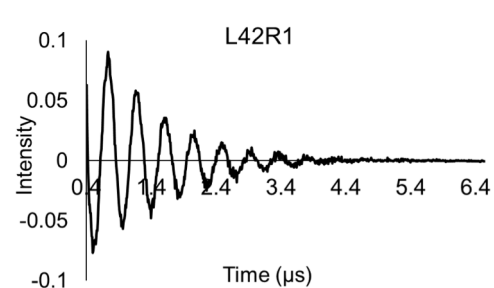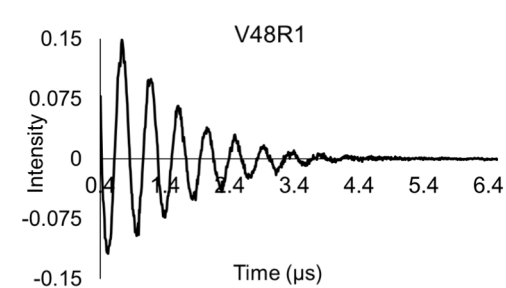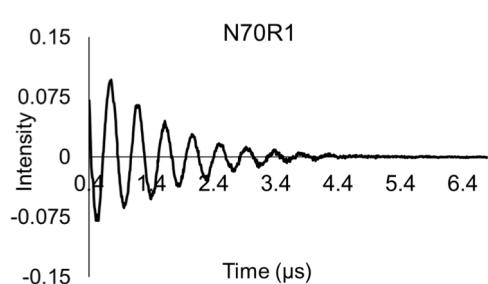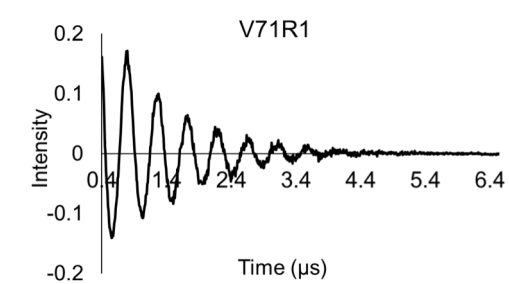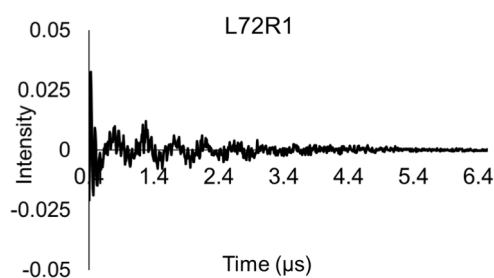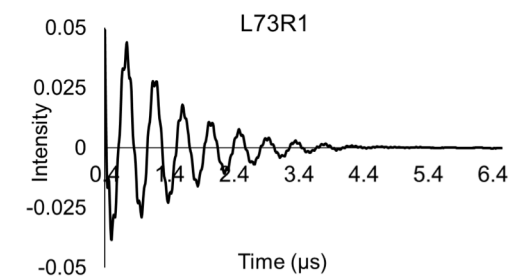

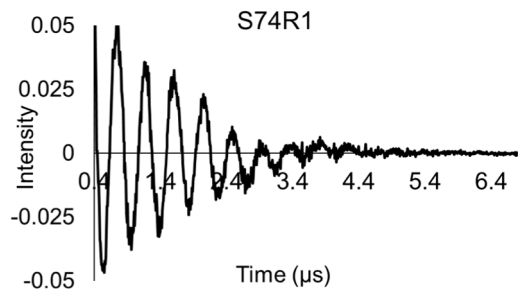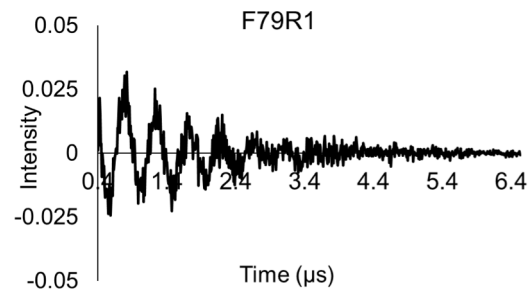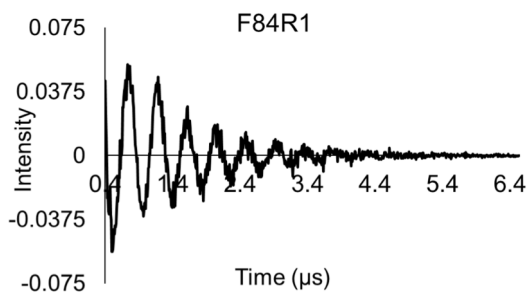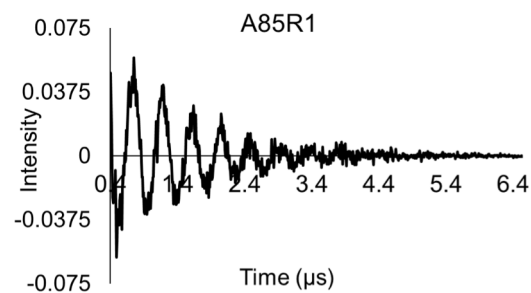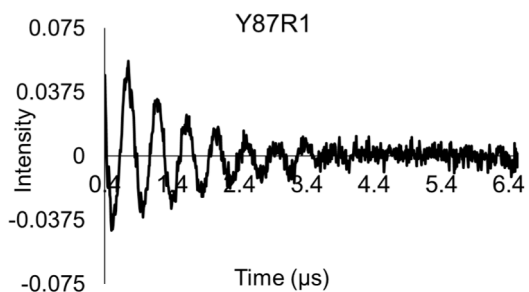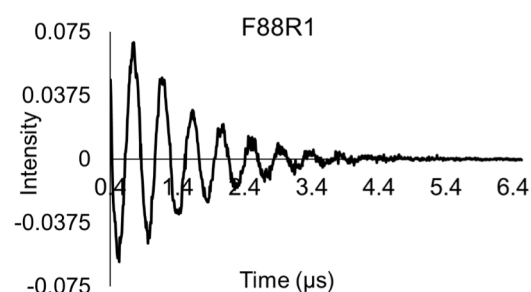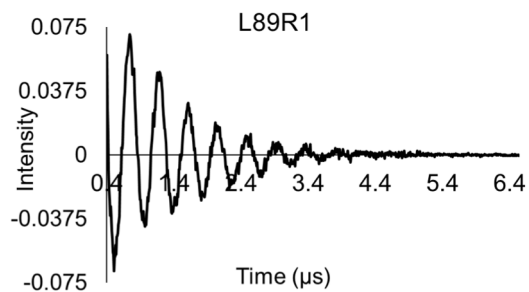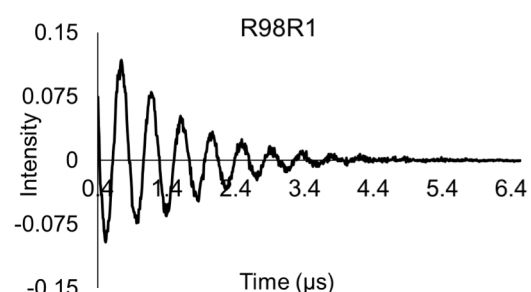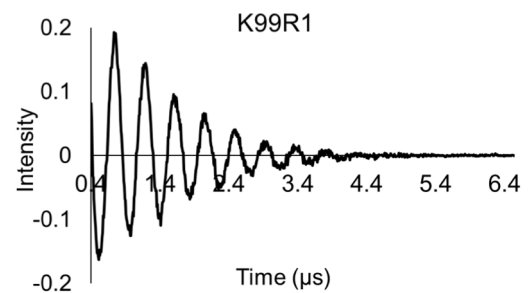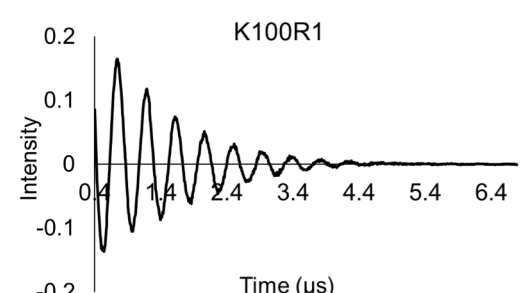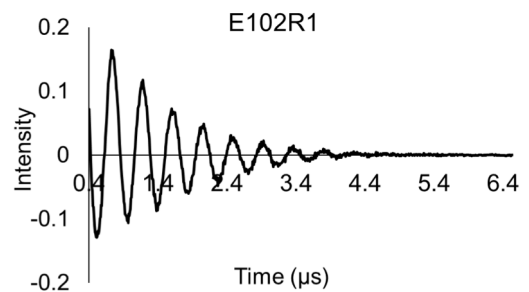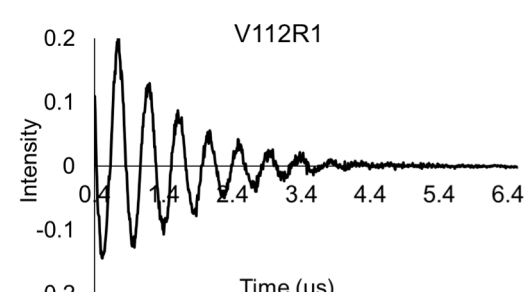

Supplement: Data S1. Background-corrected time-domain 3pESEEM raw experimental spectra used for the solvent accessibility determination of spin-labeled TbMscL residues, related to Figures 2 and 3 [file mmc2.pdf]

N13R1

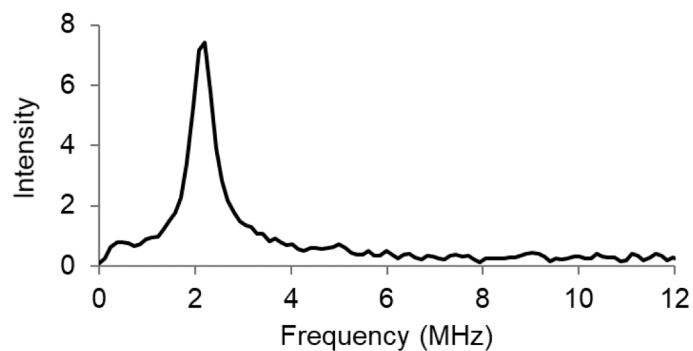

N13R1/L89W

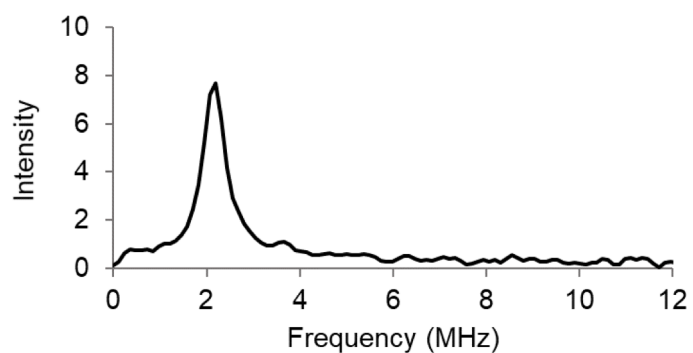

V21R1

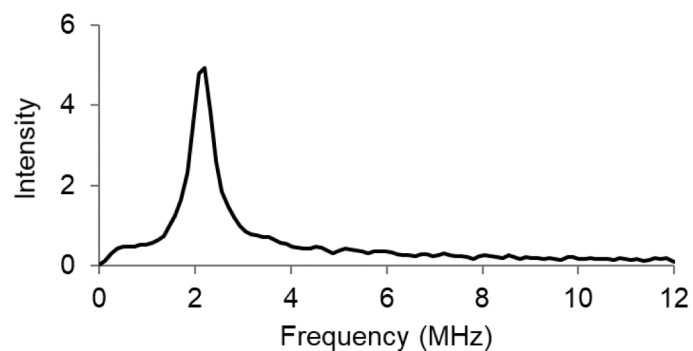

V21R1/L89W

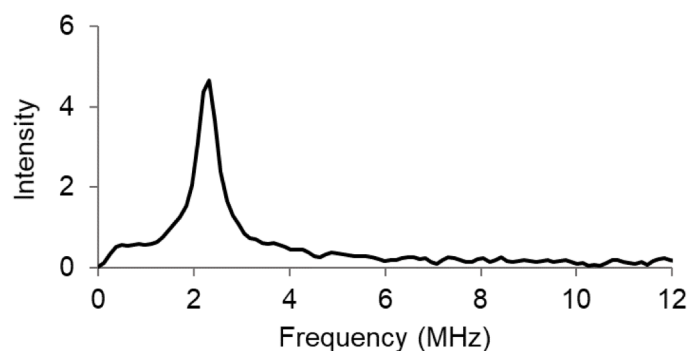

L42R1

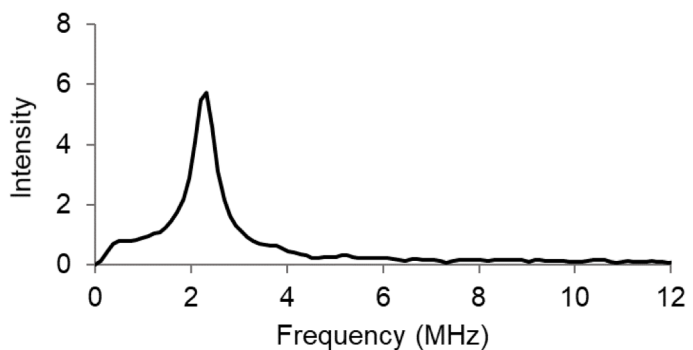

L42R1/L89W

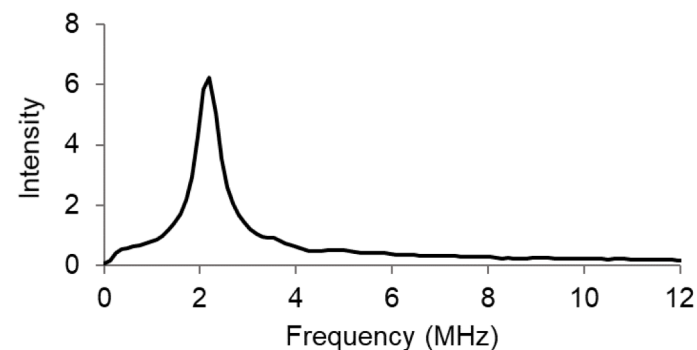

N70R1

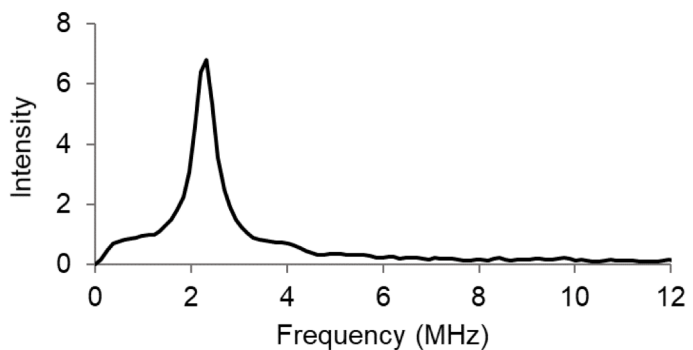

N70R1/L89W

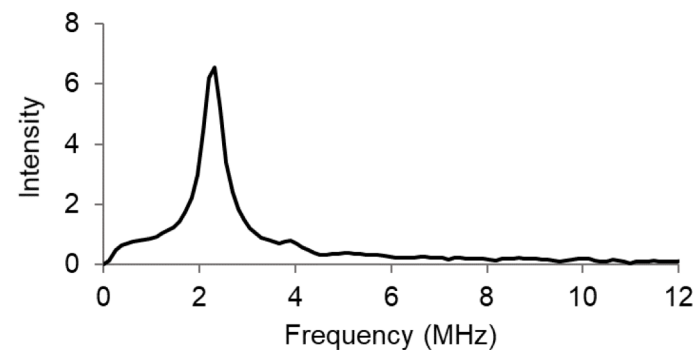

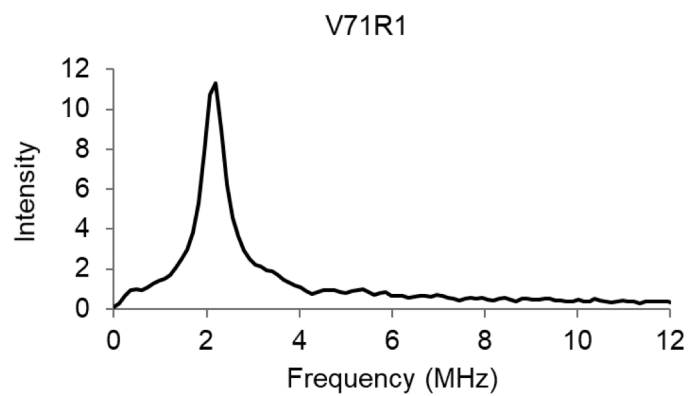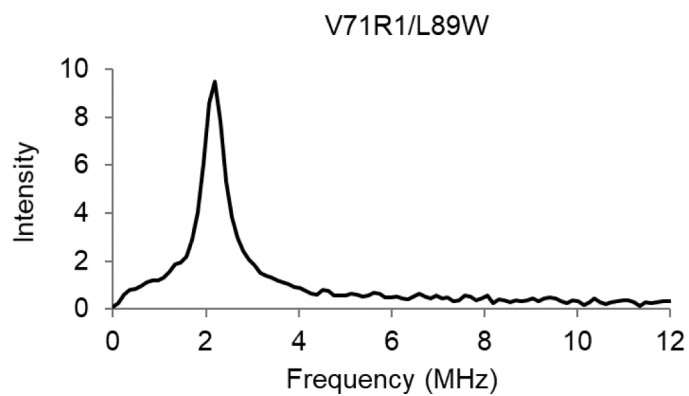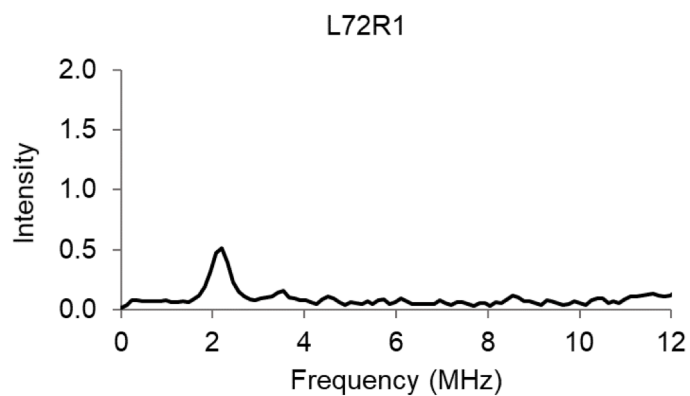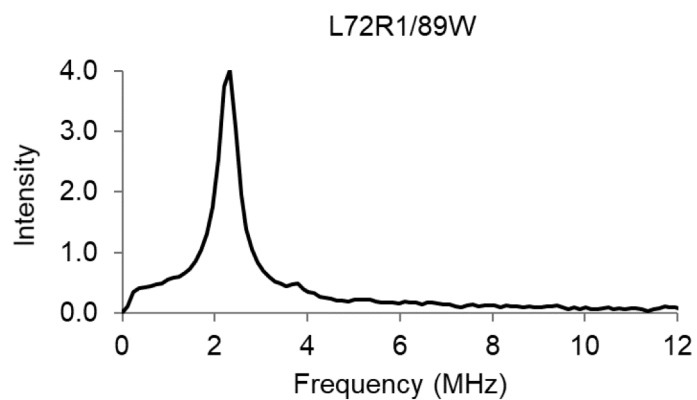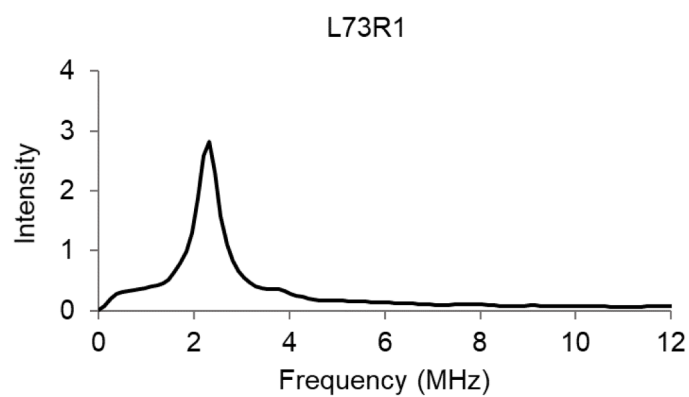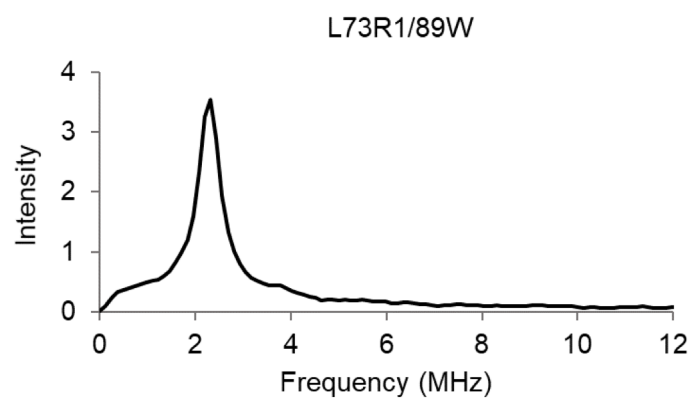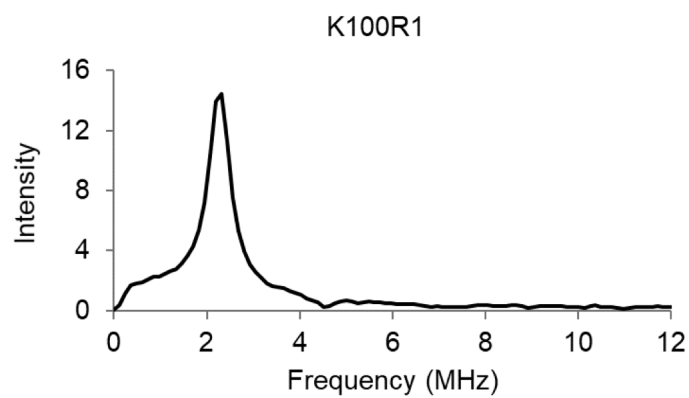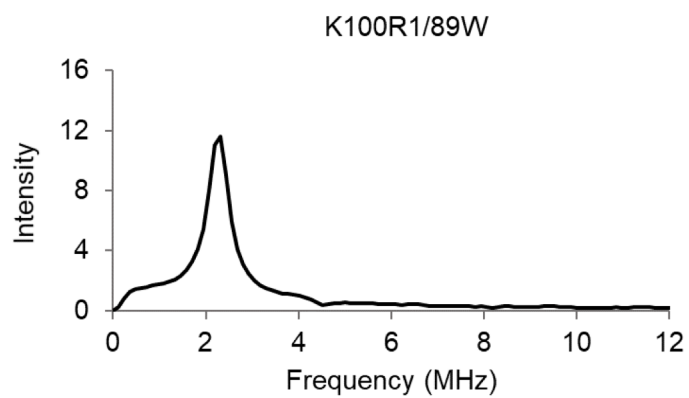

Supplement: Data S2. Frequency domain spectra of 3pESEEM data used for solvent accessibility determination of TbMscL spin-labeled residues 13R1, 13R1/89W, 21R1, 21R1/89W, 42R1, 42R1/89W, 70R1, 70R1/89W, 71R1, 71R1/89W, 72R1, 72R1/89W, 73R1, 73R1/89W, 100R1, and 100R1/89W, related to Figure 3 [file mmc3.pdf]
