# Supplementary material for: Pocket delipidation induced by membrane tension or modification leads to a structurally analogous mechanosensitive channel state
Source: Structure. 2022 Apr 7;30(4):608–622.e5. doi: 10.1016/j.str.2021.12.004 (PMC9033278; doi:10.1016/j.str.2021.12.004)
Supplement: Document S2. Article plus supplemental information [file mmc7.pdf]

Structure

Article

Pocket delipidation induced by membrane tension or modification leads to a structurally analogous mechanosensitive channel state

Graphical abstract

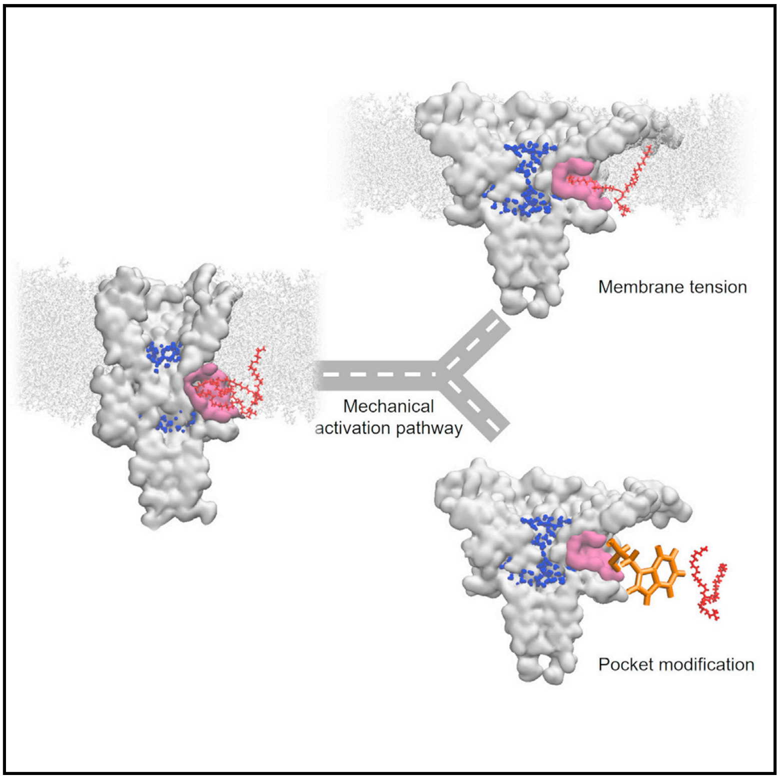

Authors

Bolin Wang, Benjamin J. Lane, Charalampos Kapsalis, ..., Antonio N. Calabrese, Antreas C. Kalli, Christos Pliotas

Correspondence

c.pliotas@leeds.ac.uk

In brief

Wang et al. demonstrate that delipidation of the MscL channel pockets by either membrane tension or modification stabilizes a structurally analogous expanded state. This is a crucial state in the mechanical activation pathway, hinting at direct links between tension-mediated and molecular activation in mechanosensitive ion channels.

Highlights

- Pocket delipidation of TM pockets stabilizes an expanded MscL state
- HDX and EPR probe conformational transitions of MscL
- Bilayer stretching MD independently generates a tension-activated state
- Structural analogy between these states has implications in MS channel regulation

Wang et al., 2022, Structure *30*, 608–622
April 7, 2022 © 2021 The Authors. Published by Elsevier Ltd.
https://doi.org/10.1016/j.str.2021.12.004

CellPress
OPEN ACCESS

Structure

Article

Pocket delipidation induced by membrane tension or modification leads to a structurally analogous mechanosensitive channel state

Bolin Wang,[1,2,7] Benjamin J. Lane,[1,2,7] Charalampos Kapsalis,[3] James R. Ault,[1,4] Frank Sobott,[1,4] Hassane El Mkami,[5] Antonio N. Calabrese,[1,4] Antreas C. Kalli,[1,6] and Christos Pliotas[1,2,3,8,*]

[1]Astbury Centre for Structural Molecular Biology, University of Leeds, Leeds LS2 9JT, UK
[2]School of Biomedical Sciences, Faculty of Biological Sciences, University of Leeds, Leeds LS2 9JT, UK
[3]Biomedical Sciences Research Complex, School of Biology, University of St Andrews, St Andrews KY16 9ST, UK
[4]School of Molecular and Cellular Biology, Faculty of Biological Sciences, University of Leeds, Leeds LS2 9JT, UK
[5]School of Physics and Astronomy, University of St Andrews, St Andrews KY16 9SS, UK
[6]Leeds Institute of Cardiovascular and Metabolic Medicine, School of Medicine, University of Leeds, Leeds LS2 9NL, UK
[7]These authors contributed equally
[8]Lead contact
*Correspondence: c.pliotas@leeds.ac.uk
https://doi.org/10.1016/j.str.2021.12.004

SUMMARY

The mechanosensitive ion channel of large conductance MscL gates in response to membrane tension changes. Lipid removal from transmembrane pockets leads to a concerted structural and functional MscL response, but it remains unknown whether there is a correlation between the tension-mediated state and the state derived by pocket delipidation in the absence of tension. Here, we combined pulsed electron paramagnetic resonance spectroscopy and hydrogen-deuterium exchange mass spectrometry, coupled with molecular dynamics simulations under membrane tension, to investigate the structural changes associated with the distinctively derived states. Whether it is tension- or modification-mediated pocket delipidation, we find that MscL samples a similar expanded subconducting state. This is the final step of the delipidation pathway, but only an intermediate stop on the tension-mediated path, with additional tension triggering further channel opening. Our findings hint at synergistic modes of regulation by lipid molecules in membrane tension-activated mechanosensitive channels.

INTRODUCTION

Specific lipid binding events and changes in transbilayer pressure can modulate the structure of membrane proteins and regulate their function (Brohawn, 2015; Cox et al., 2015; Kapsalis et al., 2019; Laganowsky et al., 2014; Malcolm et al., 2015; Naismith and Booth, 2012; Patrick et al., 2018; Pliotas et al., 2015; Pliotas and Naismith, 2016; Ridone et al., 2018; Teng et al., 2015). Mechanosensitive (MS) ion channels are integral membrane proteins that sense and respond to tension changes in the membrane. Bacterial MS channels are gated mainly by membrane tension, whereas eukaryotic MS channels are multimodal and regulated both by tension and molecular triggers. However, whether molecular and tension activation present mechanistic similarities and whether such mechanisms have evolved through the converged structural plasticity of a common origin is largely unclear. Pressure-sensitive domains, or pockets, formed within transmembrane (TM) regions of bacterial MS channels have been shown to play a role in mechanical sensing and response (Kapsalis et al., 2019; Pliotas et al., 2012, 2015; Pliotas and Naismith, 2016; Ward et al., 2014). These pockets were first identified on the MS channel of small-conductance MscS, leading to the lipids-move-first hypothesis, in which lipids accessing the pockets physically inhibit (directly or allosterically) the movement of pore helices to gate the channel (Pliotas et al., 2012, 2015; Pliotas and Naismith, 2016). The lipids-move-first model, which is consistent with an entropy-driven model for the mechanical sensing of ion channels (Kefauver et al., 2020), was further supported by recent cryoelectron microscopy (cryo-EM) studies on MscS (Flegler et al., 2021; Rasmussen et al., 2019; Zhang et al., 2021) and was extended to the structurally unrelated (to MscS) MscL channel, suggesting an applicability of the model to structurally diverse MS channels (Kapsalis et al., 2019, 2020). When a modification (L89W) was introduced at the entrance of the tension-sensitive pockets in the MscL channel, steric lipid interactions with the backside of the pore-lining helix TM1 were significantly reduced, and as a consequence, MscL expanded, adopting an intermediate state in the absence of tension (Kapsalis et al., 2019, 2020). The mechanical response of MscL suggested that pocket-targeting lipids could act as negative allosteric modulators for MS channels and that tension could be mimicked by molecules or gain-of-function mutations (or modifications) targeting the pocket region, which could disrupt the lipid pathway between the bulk membrane and the pockets to gate the channel (Anishkin et al., 2005; Iscla et al., 2015; Kapsalis et al., 2019; Pliotas and Naismith, 2016). The function of both MscL and MscS can

 © 2021 The Authors. Published by Elsevier Ltd.
This is an open access article under the CC BY license (http://creativecommons.org/licenses/by/4.0/).

CellPress
OPEN ACCESS

be modulated by changes in molecule (including lipids) occupancy within these pockets (Flegler et al., 2021; Kapsalis et al., 2019; Pliotas et al., 2015; Wray et al., 2016, 2019; Zhang et al., 2021), and even subtle structural differences within the same regions could lead to functional differences in different MscL proteins (Kapsalis et al., 2020). This suggests a common regulatory mechanism, tailored to the unique structural landscape of MS channels, which may guide ligand specificity.

Lipids residing within similar inner-leaflet pockets have also been linked to mechanosensation in X-ray and cryo-EM structures of TRPV3 (Deng et al., 2020a), TRAAK (Brohawn et al., 2014), TREK-2 (Dong et al., 2015), YnaI (Flegler et al., 2020), MSL1 (Deng et al., 2020b), and MscS (Flegler et al., 2021; Pliotas et al., 2015; Rasmussen et al., 2019; Zhang et al., 2021). The unique parallel to membrane plane orientation of these pocket lipids may be guided by an amphipathic helix that is important in the mechanosensitivity of MscL (Bavi et al., 2016) and G protein-coupled receptors (GPCRs) (Erdogmus et al., 2019) and is essential in forming the pockets.

Attempts to model MscL channel opening have been previously implemented via steered molecular dynamics (MD) simulations, with added tensions to pull the channel open (Bavi et al., 2017a; Gullingsrud and Schulten, 2003; Jeon and Voth, 2008; Konijnenberg et al., 2014; Martinac et al., 2017), but the tension used in this modeling is an order of magnitude higher than that required to open MscL in giant unilamellar vesicles and spheroplasts (Herrera et al., 2018; Kapsalis et al., 2019; Mukherjee et al., 2014). In other cases, MscL was subjected to extensive modifications or forces were applied for relatively short periods of time (Katsuta et al., 2019; Martinac et al., 2017). MD simulations on MS TREK-2 have shown that the pockets are occupied by lipids only in the absence of membrane stretch (Aryal et al., 2017).

Hydrogen-deuterium exchange mass spectrometry (HDX-MS) and 3-pulse electron spin echo envelope modulation (3pESEEM) spectroscopy are powerful tools in the investigation of membrane protein structural dynamics. The former involves the exchange of protons on a protein with deuterium to generate accessibility information and has been used to provide key insights into the role(s) of lipids in modulating membrane protein conformation (Ahdash et al., 2019; Konermann et al., 2011; Martens et al., 2019; Moller et al., 2019). The latter measures weak hyperfine coupling between unpaired electrons and nuclear spins to probe accessibility to the solvent (D_2O) and has been used to investigate the dynamics of membrane proteins (Cieslak et al., 2010; Hartley et al., 2021; Kapsalis et al., 2019, 2020; Liu et al., 2016; Matalon et al., 2013; Pliotas, 2017; Volkov et al., 2009; Zhang et al., 2015).

Here, we endeavored to investigate whether there is a structural analogy between the physiologically relevant membrane tension-activated (mechanical) state and the one stabilized by modifications that result in pocket delipidation. Transitions between MS channel states may follow a similar pathway to cover the available conformational space, but may not necessarily sample the same discrete intermediates. To address this, we have combined untargeted (HDX-MS) and single-residue (3pESEEM) methods to probe the architecture of MscL upon pocket delipidation and independently generate a tension-activated MscL state by MD simulations. We find that these two different triggers lead to a structurally, and thus functionally analogous state, suggesting a direct link between tension and pocket lipid removal activation in MS channels. Furthermore, our data suggest that upon tension activation, additional structural changes can trigger substantial further opening of the channel pore, suggesting that the structural plasticity of these MS ion channels enables them to respond differently upon receipt of discrete stimuli.

RESULTS

Monitoring channel gating by pocket delipidation with HDX-MS

The substitution of a tryptophan at position L89 in *Mycobacterium tuberculosis* MscL (TbMscL) has been shown to restrict lipid access to channel pockets and destabilize the closed state, leading to an expanded (subconducting) MscL state (Kapsalis et al., 2019). To explore the structural transitions occurring between the two states, we used HDX-MS to measure relative differences in deuterium uptake between the wild-type (WT) (closed) and L89W (expanded by pocket delipidation) channel proteins. We succeeded in obtaining 95% peptide coverage of the entire resolved MscL structure in which the gating occurs (i.e., residues 1–125), including the entire TM domain and the largest part of the cytoplasmic helical bundle (83% overall coverage for the entire construct, including the C-terminal 6xHis-tag) (Figure S1). Differences in uptake, detected at the peptide level, between the two states allowed us to identify regions that became deprotected from exchange (i.e., regions that were more solvent exposed or less hydrogen bonded) in the subconducting L89W state (Figure 1). Three regions of MscL were identified and showed significant changes between the two states. Peptides in the regions containing residues 37–53 (periplasmic loop), 58–69 (top of TM2), and 97–111 (bottom of TM2, cytoplasmic loop, and top of the cytoplasmic helical bundle) had a significantly higher uptake of deuterium in L89W in comparison to WT TbMscL (Figure S1; Table S1). Residues 37–53 run from the top of TM1 and end after the first β-sheet in the loop connecting TM1 to TM2. Residues 58–69 correspond to the middle of the periplasmic loop (peri-loop) up until TM2, and residues 97–111 cover the bottom of TM2 to the top of the C-terminal helical bundle (CHB) (Figures 1 and S1). This is consistent with protein regions located at the cytoplasmic and membrane inner-leaflet interface, which are expected to undergo transitions and become exposed upon channel opening (Anishkin et al., 2005; Bavi et al., 2016, 2017b; Kapsalis et al., 2019; Yang et al., 2012). These HDX-MS data have highlighted the regions important in MscL gating and provided us with the first glimpses into the mechanism of activation of MscL by pocket lipid removal (triggered here by the L89W modification at the pocket entrance).

Solvent accessibility mapping at single-residue resolution by 3pESEEM

While our HDX-MS approach identified regions of change in MscL in the L89W mutant, this approach suffers from several limitations, including that the changes in deuterium uptake were measured at peptide-level resolution, and that differences in deuterium uptake can be ascribed to changes in solvent accessibility and/or changes in hydrogen bonding. Therefore, we sought to investigate the feasibility of 3pESEEM to study the structure of MscL (to identify sites

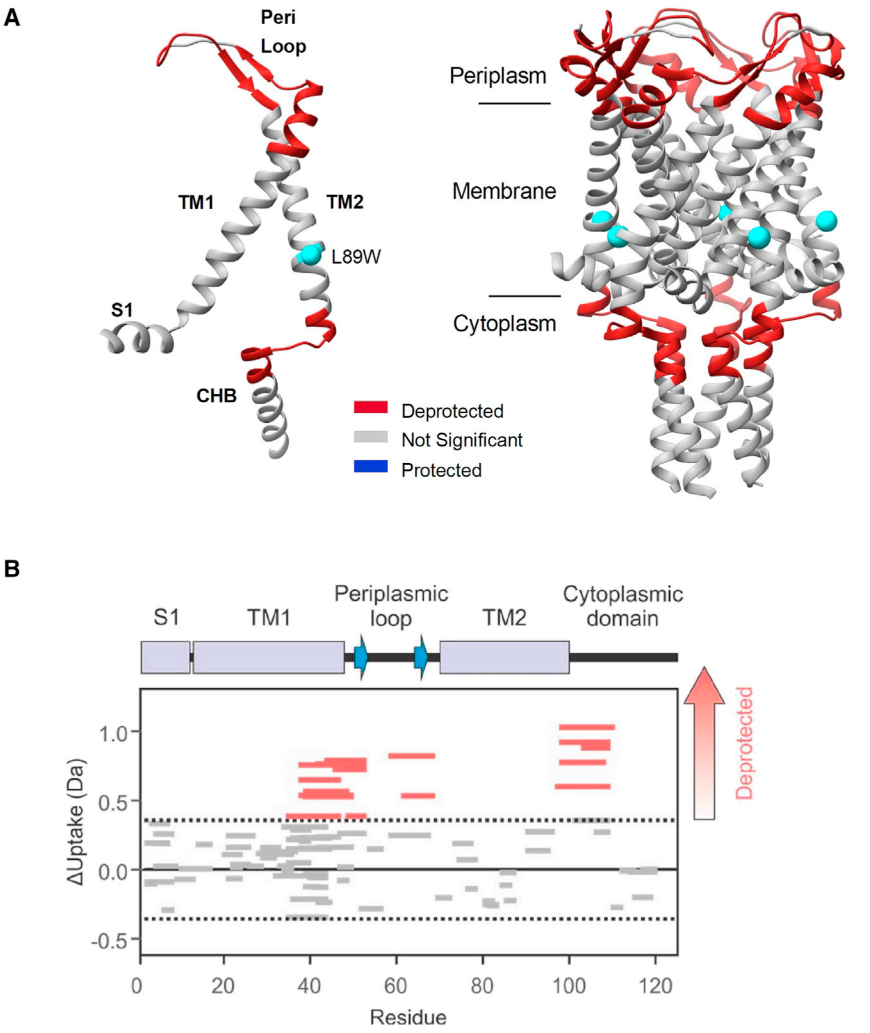

Figure 1. Global changes on MscL induced by pocket delipidation observed by HDX-MS

(A) Differences in the deuterium uptake of regions of TbMscL (PDB: 2OAR) when comparing the WT and the L89W (pocket lipid removal) modified protein. Regions highlighted in red are deprotected following the L89W modification. Regions of the protein in gray show no significant difference between the 2 conditions. L89W modification site is depicted as a cyan sphere.

(B) Wood's plots showing the summed differences in deuterium uptake in MscL over all 5 HDX time points, comparing WT with L89W MscL (Wood's plots were generated using Deuteros (Lau et al., 2019). Peptides colored in red are deprotected from exchange in L89W MscL. No peptides were significantly protected from exchange in L89W MscL compared with wild-type MscL. Peptides with no significant difference between conditions, determined using a 99% confidence interval (dotted line), are shown in gray. Example deuterium uptake curves and a map of the peptides detected are shown in Figure S1. Note that only 5 residues from residues 1–120 were not covered by peptides.

that are potentially dynamic during gating and solvent accessible), as this technology enables solvent accessibility to be probed at single-residue resolution. To this end, we generated 26 single-cysteine mutants, accounting for 20% of the total TbMscL length, and spanning all channel protein domains (e.g. S1 [cytoplasmic-facing short amphipathic helix], TM1, the peri-loop, TM2, the cytoplasmic loop, and the CHB in a Cys-free WT TbMscL background; Figure 2). We subsequently expressed, purified, and spin labeled each one of these mutants (*S*-(2,2,5,5-tetramethyl-2,5-dihydro-1H-pyrrol-3-yl)methyl methanesulfonothioate [MTSSL] modification is denoted as R1 hereafter) and performed 3pESEEM solvent accessibility measurements (Figure S2; Data S1). Solvent accessibility is based on the modulation depth of deuterium in the time domain signal, which is proportional to its associated signal intensity in the frequency domain. We used two independent analysis methods to determine solvent accessibility, both yielding very similar results (Figure S3). Unlike pulsed electron-electron double resonance (PELDOR), which requires high spin-labeling efficiency to obtain high-sensitivity data acquisition (Kapsalis et al., 2019; Pliotas et al., 2012), 3pESEEM can be performed with no major losses in sensitivity even if the sample of interest shows lower spin-labeling efficiencies for individual sites. This allowed us to obtain high-quality spectra and quantify the solvent (D_2O) accessibility for all 26 sites (Figures 2 and S4; Data S1). Residues on S1 showed low accessibility, which is consistent with previous reports suggesting that this helix is buried within the bilayer (Bavi et al., 2016). I23, V31, and F34 on the same side of TM1 are intermediately solvent exposed (or relatively buried) (Figures 2 and S4). This could be due to the presence of native lipids, the detergent used for membrane protein extraction, and/or hinderance by the presence of TM2 on the TM1 interface. L42 and V48 are intermediately to highly exposed, as they form part of the peri-loop connecting TM1 and TM2. N70, V71, L72, L73, and S74 present the largest disparity across our whole dataset. L72 is the most buried residue we measured (~3% compared to the most solvent-exposed cytoplasmic-facing labeled residue K100R1), while N70 (~90%) and V71 (~80%) are 2 of the most exposed sites (Figures 2 and S4; Data S1). The remaining residues present intermediate accessibilities to these 2 extremes, as expected for subsequent residues, which form a helical turn, with each residue having a different spatial orientation. Residues N70 and V71 are solvent exposed in the closed MscL state, while residues L72 and L73 are buried. The latter pair of residues is known to become exposed during opening, due to an anticlockwise TM2 rotation (Kapsalis et al., 2019, 2020; Li et al., 2015). F79, F84, and A85 are relatively buried and lie at the interface of TM helices between different MscL subunits. Y87 has been identified as a lipid-binding site (Powl et al., 2005), and along with F88 and L89 presented low to intermediate accessibilities and form part of the pocket region (Kapsalis et al., 2019, 2020; Michou et al., 2019). Except for R98, which showed intermediate accessibility, K99 and K100 presented high accessibilities,

CellPress
OPEN ACCESS

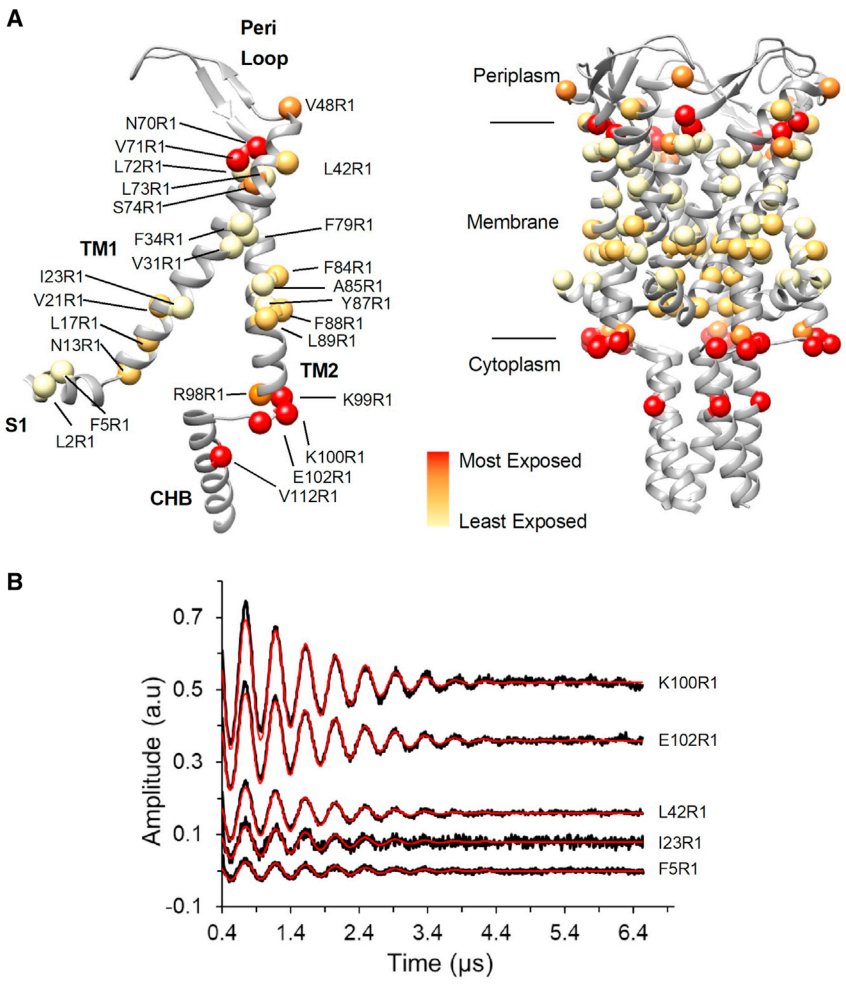

Figure 2. Single-residue mapping of MscL by 3pESEEM

(A) Solvent accessibility of spin-labeled residues are shown as red and pale yellow spheres for the most and the least solvent accessible, respectively. Spin-labeled residues are colored based on the quartile of their relative accessibility, which has been normalized on a scale of 0% and 100%, where the residue with the highest accessibility corresponds to 100%.
(B) Background-corrected time-domain 3pESEEM experimental spectra with fitting for representative spin-labeled mutants. Residue F5R1 is found on the S1 amphipathic helix, I23R1 and L42R1 on TM1, and K100R1 and E102R1 are at the interface between TM2 and the CHB.

indicating that these residues are solvent exposed in the closed state. R98, K99, and K100 form a positively charged cluster for attracting negatively charged lipids (Powl et al., 2005), and our data suggest that the non-ionic *N*-dodecyl-b-D-maltopyranoside (DDM) binds either differently or at a different site. Substitution of these residues with the neutral Gln did not affect the conformation of MscL, suggesting that specific lipid head group binding on this particular site does not influence MscL gating, demonstrated previously using PELDOR measurements (Kapsalis et al., 2019). E102 and V112, which are located in the cytoplasmic loop and the upper portion of CHB, respectively, and are expected to move apart upon MscL opening (Bavi et al., 2017b), presented high solvent accessibilities. This has enabled us to validate 3pESEEM as a suitable tool to map the structure of MscL and provides evidence that HDX-MS and 3pESEEM can afford complementary information on solvent accessibility and dynamics for integrative structural studies.

Monitoring channel gating with single-residue resolution by 3pESEEM

Typical HDX-MS experiments are limited to peptide-level resolution, although advances in MS fragmentation technologies (e.g., electron transfer dissociation) and data deconvolution algorithms are enabling residue-level uptake data to be realized. However, it remains challenging to resolve changes in deuterium uptake that occur at different residues within the same peptide. Here, we have already shown that the solvent accessibility of individual residues of MscL can be probed using 3pESEEM. Therefore, we used this approach to generate higher (single-residue) resolution information about the regions of structural change identified by HDX-MS, as well as to the hydrophobic gate (or vapor lock) of the channel, which restricts water molecule access through its pore and controls channel conductance (Figures 2A and 3B). In each of these regions, we introduced 8 distinct single-cysteine substitutions in an L89W MscL background for comparison with identical sites in the background of the WT protein, specifically, N13 and V21 (pore), L42 in the peri-loop, N70, V71, L72, L73 (top of TM2), and K100 (bottom of TM2 and cytoplasmic loop) (Figures 3, S5, and S6; Data S2). V21 forms the vapor lock (along with L17) that controls the channel gate, and N13 sits 1 turn of a helix below. No significant change in this region was highlighted via HDX-MS, while due to the proximity of spin labels pointing toward the pore, distances are out of the distance measurement range (<19 Å) of PELDOR (Figures 1 and 3B). However, 3pESEEM is highly sensitive to the local spin label environment, does not need a reference state (as is the case for HDX-MS), and is not subject to distance restriction between sites as for PELDOR. Differences in the vapor-lock region may not have been detected by HDX-MS as structural changes may have resulted in complex changes to hydrogen bonding networks, whereby some residues were deprotected from exchange and others became protected, resulting in only minor or no changes in deuterium uptake at the peptide level (Figures 1 and S1). To resolve this, we labeled the V21 and N13 sites crucial for forming the pore vapor lock and 6 other sites within the regions identified by HDX-MS, which undergo conformational changes when the L89W modification is present (Figures 3, S5, and S6; Data S2). Overall, the motivation behind this was to confirm the peptide-level HDX-MS data and complement this with single-residue

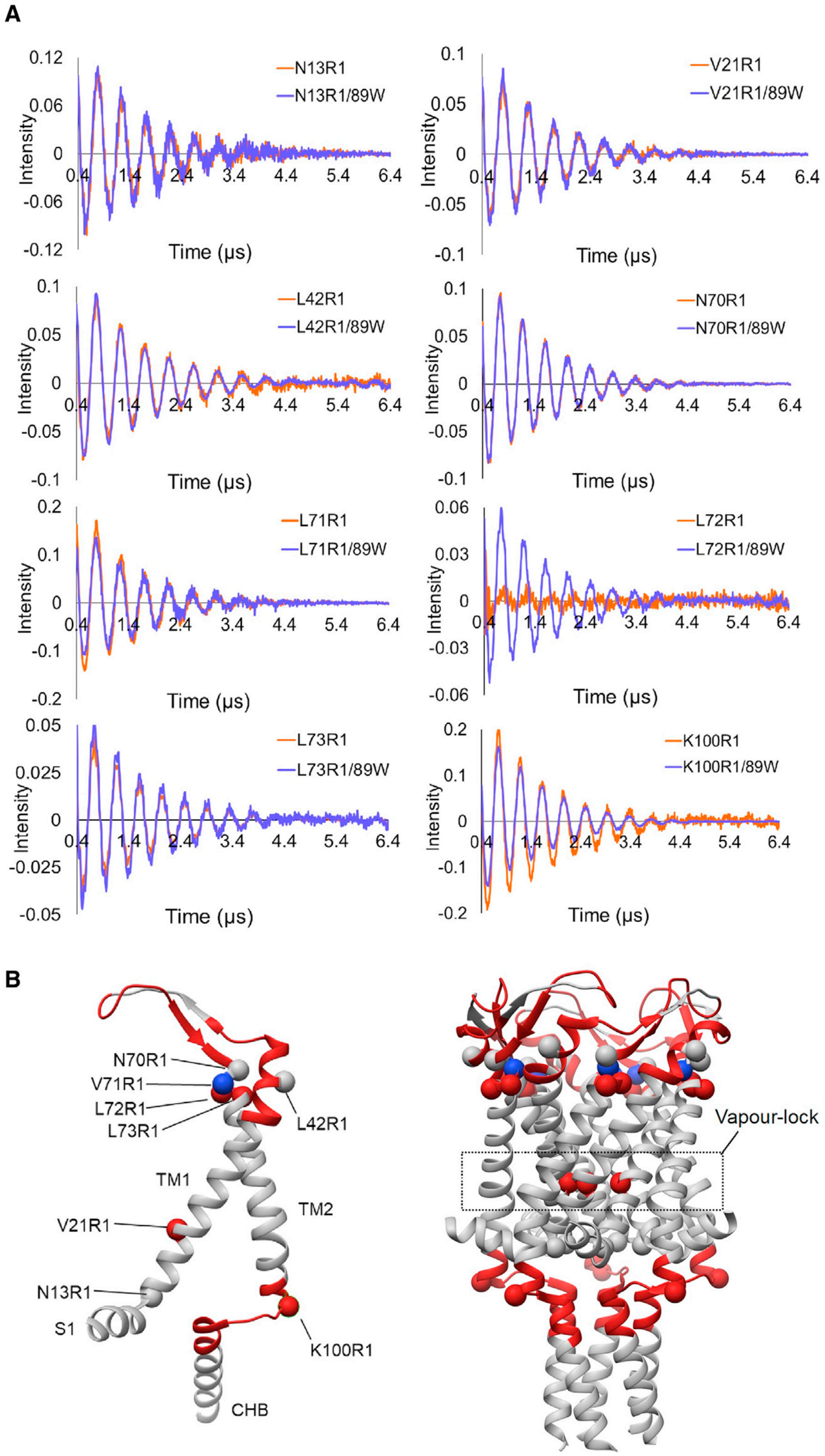

Figure 3. Effect of pocket delipidation on MscL structure investigated by 3pESEEM and HDX-MS

(A) Background-corrected time-domain 3pESEEM experimental spectra (orange) of the single mutants N13R1, V21R1, L42R1, N70R1, L72R1, L73R1, and K100R1 in DDM overlaid with their associated L89W double-mutant spectra (purple).

(B) Differences in solvent accessibility for TbMscL (PDB: 2OAR) following L89W modification. Spin-labeled mutation sites used for 3pESEEM accessibility measurements are represented by spheres, and peptides that demonstrate a change in accessibility in HDX-MS following the L89W modification are represented as highlighted helices. Red regions or spheres highlight areas that are deprotected, while blue spheres and regions show areas that are protected following the L89W modification. There was no significant difference in the solvent accessibility of N13R1, L42R1, and N70R1 compared to their 89W double-mutant counterpart. Solvent accessibility increased for V21R1 and L72R1 and decreased for L71R1 and K100R1 following the L89W modification. The associated column bar representation including errors are described in Figure S6.

resolution 3pESEEM data for crucial sites where changes may have not been detected due to limitations associated with peptide-level analysis in HDX-MS.

3pESEEM measurements at position L42, located in the periloop, showed no significant differences in solvent accessibility between the WT and the L89W states. For N13, which points toward the cytoplasm and is already solvent exposed in the closed state, we observed similar exposure in the expanded L89W MscL state (Figures 3, S6, and S7; Data S2). In L89W MscL, the solvent accessibility increased for V21, which is consistent with channel pore hydration, as its side chain sits within the TM domain in the closed state (Figure S7). A >10-fold increase in solvent accessibility was observed for L72, while a significant decrease was observed for K100, which sits at the C-terminal end of TM2 (Figures 3 and S6). Although N70A showed no change, a significant decrease in solvent accessibility (∼19%) was observed for V71 in an L89W MscL background. Despite being consecutive residues, 70–73 show distinct changes in solvent accessibility in 3pESEEM, unlike HDX-MS, which reported an average difference in deuterium uptake at the peptide level. A significant and smaller increase in accessibility is seen for L72 and L73, respectively, which is the opposite of the effect observed for N70 and V71, while the bottom of TM2 (K100) becomes less accessible to solvent in the L89W background (Figures 3 and S6). This result is consistent with a rotation of the top of TM2, which occurs during mechanical activation and agrees with PELDOR distance measurements and X-ray structures (Kapsalis et al., 2019, 2020; Li et al., 2015). HDX-MS experiments showed deprotection on peptides inclusive of these residues and are

consistent with a dramatic increase in the accessibility of residue L72 upon pocket lipid removal activation (i.e., when the L89W mutation is present) in 3pESEEM measurements (Figures 3 and S6). V21 forms the vapor lock for TbMscL and shows an increase in solvent accessibility in an L89W background, consistent with the pore gate hydration and the channel entering an expanded (and intermediate for full opening) state. HDX-MS measurements show a deprotection of the TM2 bottom for L89W versus WT, suggesting an overall solvent exposure of this region (Figures 1 and 3). The MscL cytoplasmic loop has been shown to participate in gating and provide access to streptomycin entering the cell, while shortening of the loop influences the function of MscL by irreversibly decreasing its conductance (Wray et al., 2016; Yang et al., 2012). Combined, HDX-MS and 3pESSEM allowed us to identify entire regions and individual residues on MscL, which undergo substantial conformational changes upon pocket delipidation (L89W background).

Membrane stretching promotes pore hydration in the WT channel

To mimic the naturally occurring activation of MscL by membrane tension, we implemented MD simulations on the WT TbMscL in lipids, with and without tension applied to the lipid bilayer. We performed all simulations with the same lipid composition to compare the pore properties and overall channel architecture between the states. Our simulations were initiated from the TbMscL X-ray structure (PDB: 2OAR) (Chang et al., 1998) carrying no modifications. Previously, significantly higher bilayer tension was required to open MscL in MD simulations over short periods of simulation time (Bavi et al., 2017a; Gullingsrud and Schulten, 2003; Martinac et al., 2017), in comparison to the lower tension (~67.5 mN/m) and longer simulation times (300 ns) used in this study, allowing us to capture intermediate states.

WT TbMscL was inserted in a 1-palmitoyl-2-oleoyl-sn-glycero-3-phosphocholine (POPC) bilayer, and simulations for 300 ns were performed in which the x- and y-pressure of the box was set at −50 bar, creating constant bilayer tension. During the simulations, the bilayer could expand to adapt to the expansion occurring in the membrane plane. As a result, the bilayer thickness decreased ~1.2 nm compared with the POPC bilayer without tension (Figures 4B and 5; Videos S1 and S2). The channel underwent large conformational changes, reaching an overall root-mean-square deviation (RMSD) of ~14 Å compared with the initial closed structure to adapt to the changes in the bilayer by tilting its TM helices toward the membrane plane (Figures 4A and S8A; Video S3). In particular, TM1 and TM2 gradually tilted by 30° and 15°, respectively, toward the end of the simulations, while the pressure-sensitive pocket surface and the area exposed to the bilayer decreased dramatically (Figures 4, S8B, and S8C). Increasing the timescales of the simulations was outside the scope of this study, as our main interest was to trap and investigate in detail this intermediately open state and was the first stop on the tension-mediated activation pathway of MscL. This has enabled us to compare this model of a tension-activated state with our experimentally derived state obtained by pocket lipid removal, in the absence of membrane tension (Kapsalis et al., 2019).

Analysis of pore hydration profiles using the Channel Annotation Package (CHAP) (Klesse et al., 2019) showed opening and hydration of the WT channel pore under tension in all of our simulations (Figure 5A). While the higher (compared to biologically relevant) membrane tension does not allow us to provide information about the timescales of the channel transition from the closed to the hydrated state, it enables us to obtain structural data for a key intermediate state, which has a hydrated partially open pore, essential in the gating process of MscL. For these pore hydration events, we monitored the annular lipids, which make direct contact with or reside close to the channel and simultaneously interrogated the pore radius profile using HOLE (Smart et al., 1993) (Figures 5B, 5C, and S8D). For WT, we observed that following constant tension application for ~200 ns, the pore becomes hydrated, and the channel undergoes major structural rearrangements (Figure 5A). Although a significant membrane thinning (~1.2 nm) occurs, the pore of L89W cannot open to allow water molecules to flow through, and the pore radius is still restricted to ~1 Å, despite global structure changes similar to those of the WT channel (Figures 5B, 5C, and S8; Table S2). Following bilayer stretching, the total pocket surface area decreases and limits access and availability to bilayer lipids throughout our simulations (Figure 5B). Overall, the under-tension WT MscL MD data agree well with the HDX-MS and 3pESEEM accessibility data obtained for L89W (pocket delipidation by modification). In both cases, similar channel regions become accessible to the solvent, suggesting a high similarity between the obtained expanded states (Figure 7A).

Safety pin-acting trapped lipids in the pockets do not allow MscL pore hydration

A direct consequence of the lipids-move-first model is that if lipids are trapped within the pockets, then the channel pore will not be able to open under membrane tension application. To test this hypothesis, we performed MD simulations with L89W TbMscL under bilayer tension and the same conditions we used for the WT protein (POPC lipid bilayer, pressure value, and simulation time). We tested whether L89W modification causes any distortion on the secondary structure of TbMscL. To this end, we calculated an overall RMSD of 1.8 Å between the L89W and WT TbMscL following equilibration in lipids (and in the absence of applied tension), suggesting that there is a negligible impact on the structure of MscL imposed by the L89W mutation. We observed that under bilayer tension, L89W undergoes similar global structural changes as the WT TbMscL. In all of our simulations, the modified pore of MscL does not become hydrated, even under the tension conditions that previously promoted WT pore hydration (Figures 5A, 7B, and S8; Table S2).

We observed that during the equilibration of our simulations, lipids intercalated into the pockets and occupied them. Following tension application, lipids were pulled toward the membrane, but the bulky tryptophan modification at the entrance disrupted the lipid exit path, despite that tension application sufficient to open the WT channel was applied (Figure 5). Interestingly, in the tension-activated state, F79 and L89 from adjacent subunits come very close to forming two new smaller pockets in the inner and outer leaflets of the now substantially thinner membrane (Figures 6A and 6B). This spatial "refinement" may facilitate the next stage in the activation of MscL, which follows this subconducting state and requires additional and highly

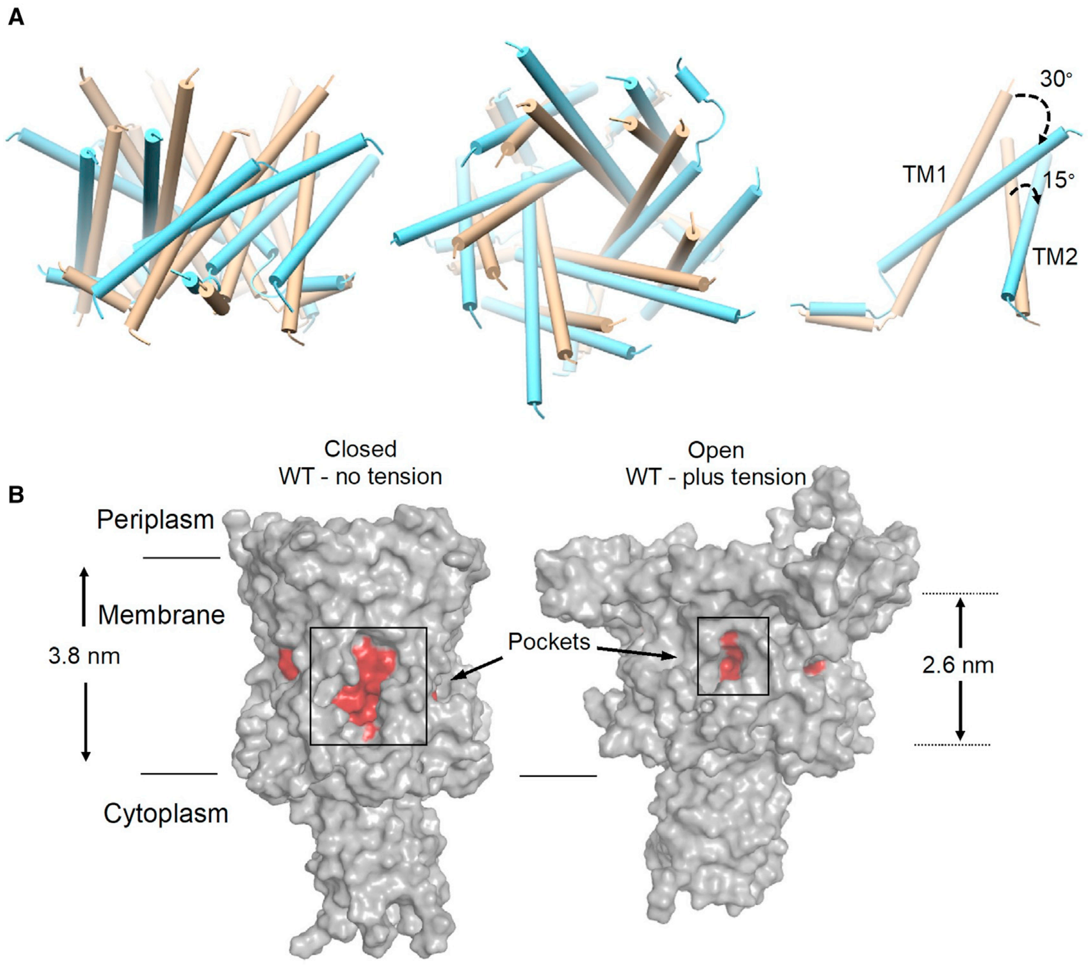

Figure 4. Tension-mediated expanded MscL state

(A) TM comparison between closed TbMscL (tan, PDB: 2OAR) and open state WT MscLs generated by MD simulations (cyan, under tension)—side, top, and single subunit views. The latter shows a substantial tilting of TM1 and TM2 toward the membrane plane. These conformational changes occurring under tension are more evident in Videos S1, S2, and S3.

(B) The pocket's surface dramatically decreases upon tension application, limiting lipid access. Uniform membrane thinning of 1.2 nm (3.8–2.6 nm) also occurs during channel expansion, due to tension application.

localized pressure for a full pore opening of 3 nS and ~30 Å (Kapsalis et al., 2019). This suggests that the expanded MscL state is an intermediate stop on the tension-mediated activation path and not the final destination (e.g., as in activation caused by pocket lipid removal of MscL). Asymmetry in bilayer tension sensitivity has been observed for MscL, MscS, and other mammalian MS channels (Belyy et al., 2010; Perozo et al., 2002), and we observed that inner-leaflet lipids previously occupying the pockets of MscL to keep the channel closed could subsequently penetrate either of the two newly created subpockets (Figures 5B, 6A, and 6B). This change restricts the access of new lipids from the bulk membrane to the pockets and immobilizes trapped lipids within the pockets that entered initially during equilibration. In such a case, these lipids should interact more strongly with residue 89 in the modified (W89) than in the WT (L89) channel. To test this hypothesis, we calculated the pairwise energy forces between all lipids and residue 89, L and W, for the WT and modified channels, respectively (Table 1). We found that the lipid interaction (Lennard-Jones) with residue 89 is twice as strong in the modified channel compared with the WT channel (Table 1). This suggests that lipids are *stuck* by the modification in the pockets, reducing their ability to exchange with the bulk bilayer during tension application (Figures 6A and 6B). Despite the substantial global structural rearrangements, we observed that in L89W MscL under tension, when lipids remain tightly associated with the pockets, its pore cannot hydrate (Figures 5 and S8D).

Lipid order and accessibility during tension application

To assess the lipid contact profile with the MscL TM domain, we calculated the relative difference of lipid contacts between the with tension and no tension TbMscL states (Figures 6 and S9). We found that the relative lipid contacts within the bilayer-facing residues on the TM domain were reduced over the course of the

CellPress
OPEN ACCESS

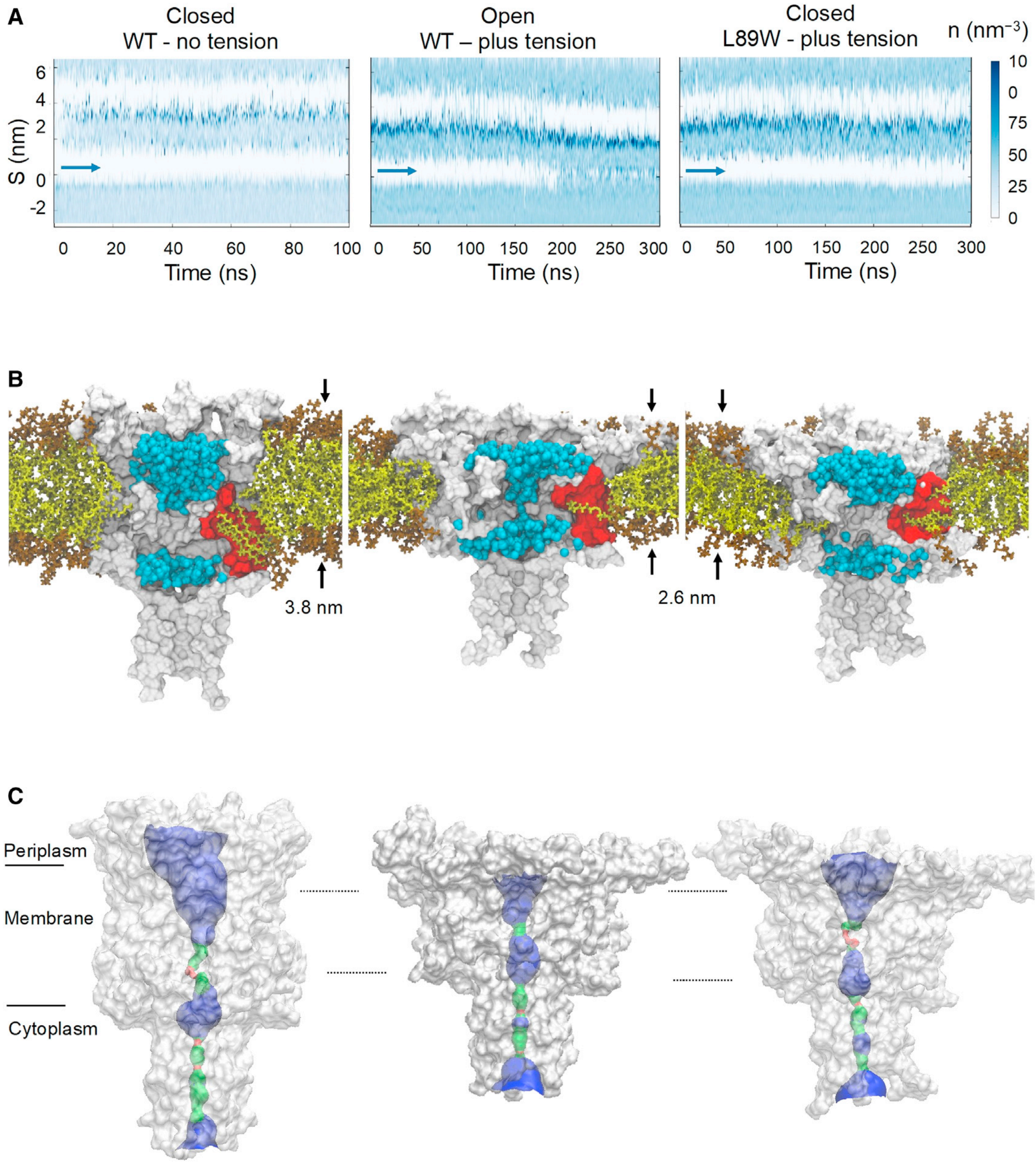

Figure 5. MscL pore hydration investigation under applied bilayer tension

WT under no tension (left column), WT under bilayer tension (right column), and L89W under bilayer tension (right column).

(A) Solvent density profile of the MD simulations using the CHAP (Klesse et al., 2019) software, with the vapor-lock position of MscL marked with blue arrows.

(B) Membrane becomes thinner when tension is applied, but only the WT TbMscL pore is hydrated (blue spheres), in contrast to L89W channel. Lipid (olive sticks) availability is larger in the closed state, and lipids have easier access within the pockets to provide force on the back of the vapor lock, keeping the MscL pore closed. This is in contrast to WT MscL under tension (center column), where the pockets become smaller, while lipid availability decreases, and the pore becomes hydrated. However, when lipids are trapped in the pockets of the L89W modified channel, the pore does not become hydrated, despite structural rearrangements occurring (right column).

(C) Surface visualization of the pore pathway using the program HOLE (Smart et al., 1993). Red color indicates a pore radius smaller than 1.15 Å (water molecules cannot go through such an opening), blue represents a radius larger than 2.3 Å, and green represents a radius between 1.15 and 2.3 Å.

simulation. In contrast, the lipid contacts of hydrophobic residues that were not lipid exposed in the closed state increased substantially (Figures 6 and S9). This is consistent with TM2 rotation occurring during MscL opening (Kapsalis et al., 2019) and with pockets decreasing their surface contact area and becoming less accessible to lipids (Figure 4B). To understand lipid rearrangement occurring upon tension application, we calculated the lipid order before and after bilayer tension application and found that all of the lipids (particularly the end of the lipid chains) orient substantially more horizontally in the presence of tension (Figures S10A and S10C). However, the pocket lipids specifically orient marginally more horizontal compared

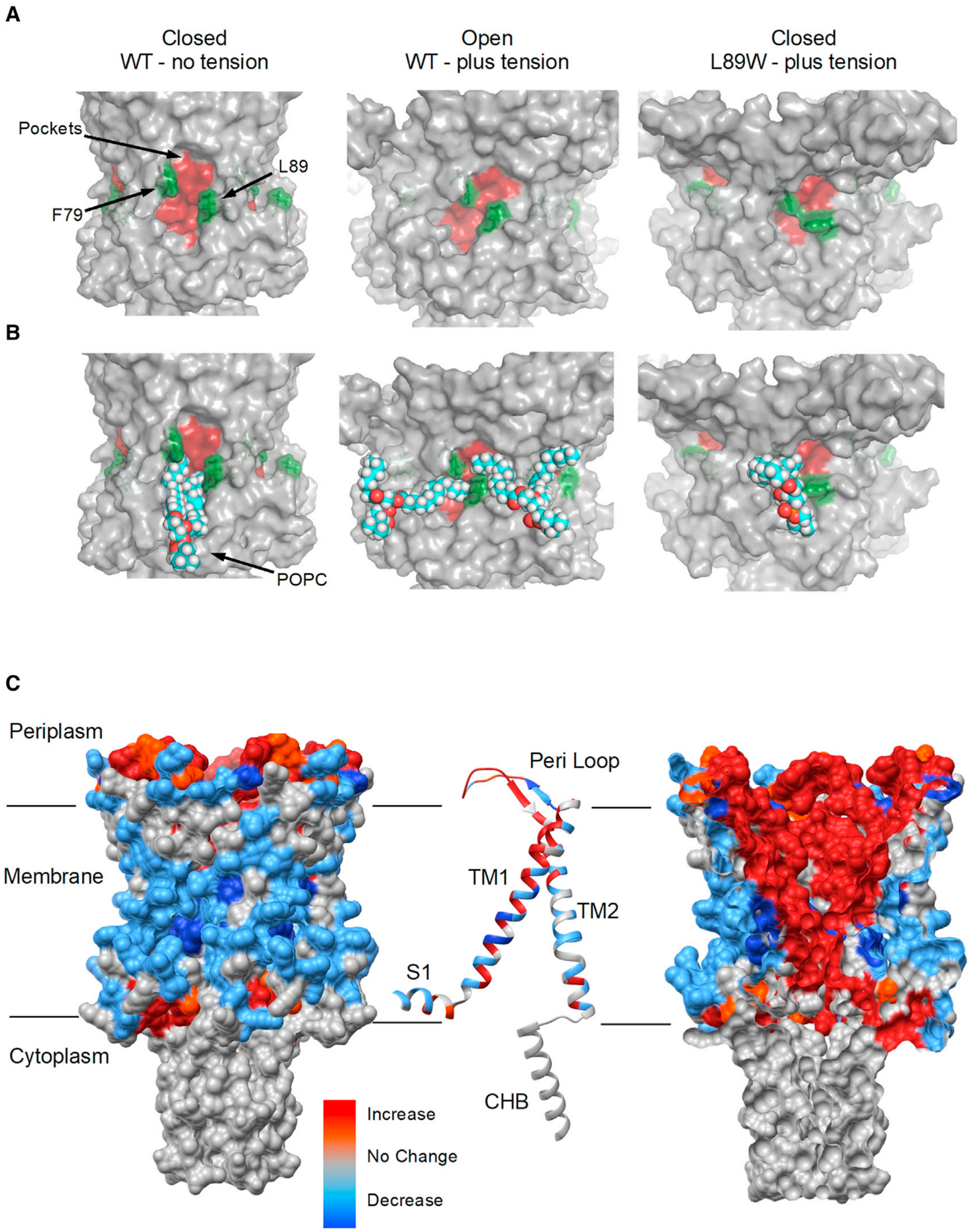

Figure 6. The effect of membrane tension to MscL protein-lipid contact interactions

(A and B) F79 and L89W form a *molecular bridge* in the L89W under tension state and consequently trap the lipids within the pockets (right column). F79 and L89 come into greater proximity under bilayer tension in the WT channel (expanded MscL, center column) compared to the closed state. However, they do not prevent the lipids from exchanging with the bulk bilayer during tension application. The L89W mutation locks lipids in the pockets, preventing the channel from transiting to a hydrated state.

(C) Relative changes in the number of lipid contacts following stimulated tension application in the membrane during MD. The blue regions show a decrease in lipid contacts, while the red regions show an increase in lipid contacts. From left to right: surface view, single subunit illustration representation, and internal MscL surface view. Internal channel regions become more exposed to lipids, while initially, membrane-exposed regions (closed state) become less exposed to lipids, suggesting a MscL TM helical rotation upon tension application and channel expansion or opening.

Table 1. Pairwise force energy calculations between TbMscL 89 (L or W) and lipids in the WT (L) and pocket-entrance-modified (W) channels under tension

89- POPC	Coulomb (KJ/mol)	SD coulomb (KJ/mol)	Lennard-Jones (KJ/mol)	SD Lennard-Jones (KJ/mol)	Total energy (KJ/mol)	SD total energy (KJ/mol)
WT	−14.50	1.76	−101.39	27.43	−115.89	29.19
L89W	−14.77	3.90	−192.48	3.39	−207.25	7.29

with the bulk bilayer lipids, in the presence of tension (Figure S10B). These findings suggest that although tension application results in a more horizontal lipid reorientation, with respect to the membrane plane, additional horizontal tilting other than that caused by membrane stretching is not required to favor lipid pocket preference or specificity (Kapsalis et al., 2019; Laganowsky et al., 2014).

DISCUSSION

According to the lipids-move-first model (Pliotas et al., 2015), MS channels respond and open their pore when under membrane tension or when lipid occupancy within the pockets is reduced. Equally the pore of the channel will not open if lipids are trapped within the pockets. It was not previously known whether the functional subconducting intermediate states adopted by MscL upon either membrane tension or pocket delipidation were analogous in MscL. Transitions between functional states of MscL may follow a similar path to cover the available conformational space between these states, but they may not necessarily sample the exact same discrete stable intermediates. Here, we find that both activation mechanisms (mechanical or pocket lipid removal) result in MscLs adopting a well-defined, expanded state.

Our accumulative data suggest that this could be the final destination of the pocket delipidation path, but only an intermediate stop on the tension-mediated activation path. Therefore, pocket lipid removal constitutes a major component of the initial stages (to open or not to open) of tension-mediated mechanical activation in MscL, consistent with pocket lipids acting akin to safety pins in a grenade (Kapsalis et al., 2019). Following bilayer tension application to WT and L89W channels, we observe similar global structural rearrangements (Figures 5 and 7). Despite this, only the WT channel pore increased in diameter enough for pore hydration. The pore of the L89W channel was significantly smaller in volume and diameter (Figure 5C and S8). Other previously reported loss-of-function or gain-of-function mutations at or in proximity to the lipid-binding pockets interfere with MscL protein-lipid interactions and may stabilize MscL states by restricting lipid access to the pockets (Anishkin et al., 2005; Iscla et al., 2015). Previously, when lipids were sterically blocked from entering the pockets, the probability of pore opening increased and the activation threshold significantly decreased (Kapsalis et al., 2019). Equivalently, in our present MD simulations, the L89W mutation locks the exit door and traps the lipids in the pockets by restricting the space available for lipids to move out. Consequently, this disallows channel pore hydration under applied tension, which we observe in our simulations of L89W TbMscL (under membrane tension) (Figures 6A and 6B; Table 1). Lipids are loosely associated with the pockets in the hydrated WT MscL, following tension activation, but these low pairwise force contacts are not sufficient to close the channel (Figures 5B and 6B; Table 1). We find that the pockets become substantially smaller under tension and consequently less accessible to lipids (Figures 4B, 5B, and 6). A similar activation mechanism has been suggested for the small-conductance MS channel MscS, where inner-leaflet lipids residing within the pockets are required to move before outer-leaflet lipids can disengage to allow gating, and the degree of their occupancy within the pockets was linked with distinct MscS states (Pliotas et al., 2015; Flegler et al., 2021; Zhang et al., 2021). This agrees with our entropy-driven lipids-move-first model and with pocket lipids being able to exchange with the bulk bilayer despite its distant positioning away from the pockets (Anishkin et al., 2008; Flegler et al., 2021; Kefauver et al., 2020; Pliotas et al., 2015; Rasmussen et al., 2019; Reddy et al., 2019; Zhang et al., 2021). Here, we have chosen to study pocket delipidation by mutation. In the future, we will exploit advances in both EPR and HDX-MS methods for the study of MS channels reconstituted in lipid nanodiscs of a defined composition, as previously implemented for integral membrane proteins (Hartley et al., 2021; Martens et al., 2019). This will enable us to explore the effect of the controlled depletion of specific lipids from channel pockets on MS channel structure and function (Cox et al., 2021; Zhang et al., 2021).

In the modified L89W MscL, F79 and W89 form a *molecular* bridge, resulting in a 2-compartment repartitioning of the smaller pockets (Figures 6A and 6B). The WT (under tension) expanded state is substantially different from the closed state X-ray structure used (PDB: 2OAR) (Figures 4 and S8; Videos S1, S2, and S3). Importantly, TM2 movement (translation and expansion due to anticlockwise TM2 rotation) is consistent with the archaeal X-ray structure of expanded MscL (Li et al., 2015), and the expanded (and subconducting) state revealed by PELDOR (Kapsalis et al., 2019, 2020) (Figure 7A).

Previously, cross-sectional expansions not associated with a conducting pore have been proposed for the initial stages of MscL activation (Betanzos et al., 2002; Konijnenberg et al., 2014), suggesting that MscL could act as a membrane tension dampener and undergo several transitions with the pore closed (Boucher et al., 2009). Our data support the notion that there are structural transitions that occur before channel opening and agree with these models. We observed transitions that are part of the closed-closed expansion gating process, since the overall RMSD between the L89W and WT structures following tension application was small, but they were both significantly different from the closed state (PDB: 2OAR) (Figures 4, 7B, and S8). This is substantiated by the fact that the channel is unable to reach the intermediate state under applied tension after the lipids are trapped within the pockets via the L89W modification, preventing pore hydration and stabilizing a closed (non-hydrated pore) state (Figures 3, 5, and 7).

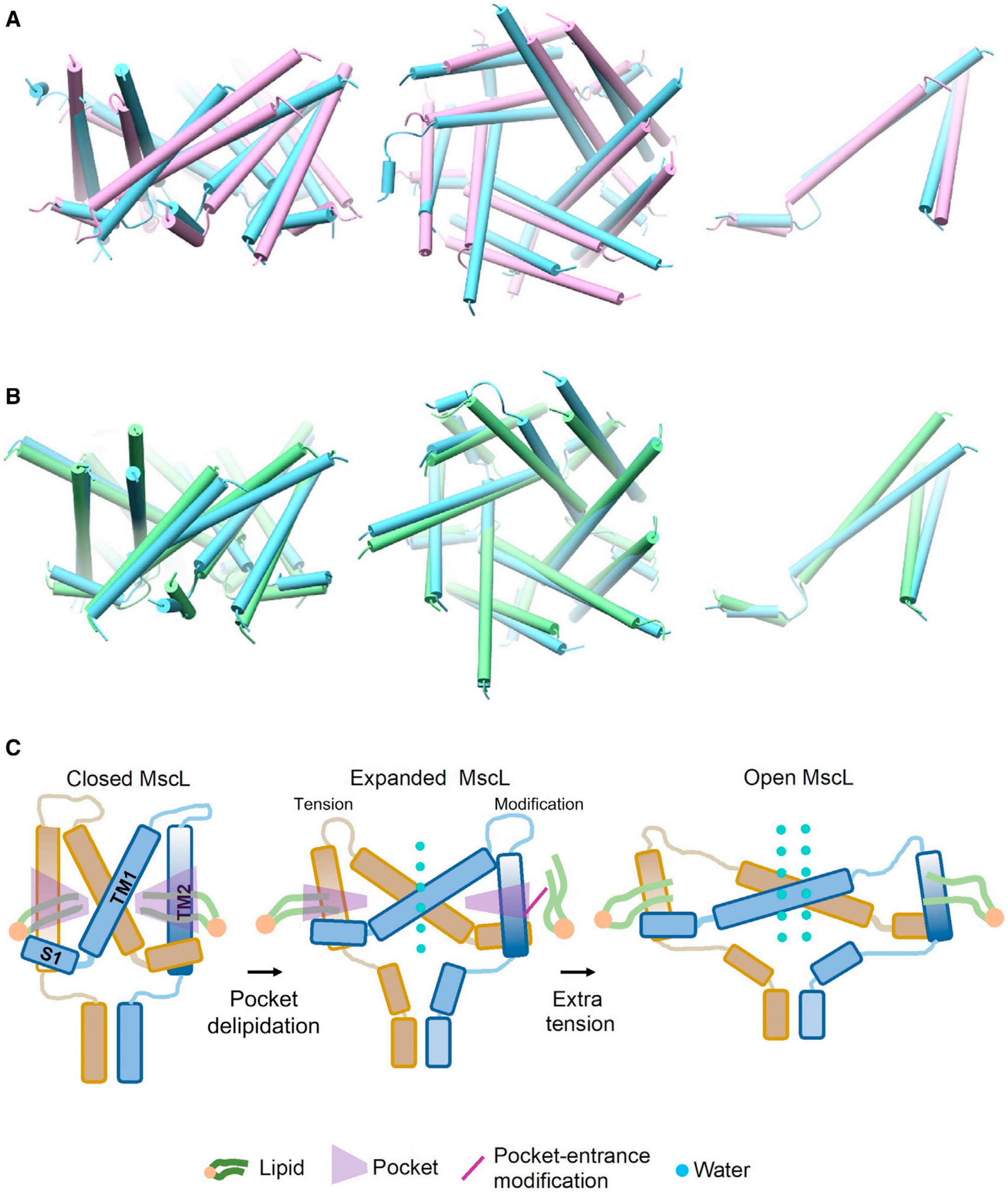

Figure 7. Comparison between tension- and modification-induced pocket delipidation MscL states and proposed model
(A and B) TM1 and TM2 tilt and rotate to initiate MscL opening. Comparison between expanded MaMscL (orchid, PDB: 4Y7J) and the open state WT TbMscL generated by MD (cyan, under tension) (A) and the open WT TbMscL state generated by MD (cyan, under tension) and L89W TbMscL (green, under tension) (B). (C) Proposed gating model for pocket lipid removal and tension-activated modes. Closed (vertical TM helices, large pockets, tightly associated annular lipids, and non-hydrated pore) and expanded (tilted TM helices, smaller pockets, loosely associated annular lipids, and hydrated pore). Extra membrane tension is required to fully open MscL.

Our data suggest that for MscL to reach the intermediate hydrated state, triggered either by tension or molecules, the lipids will have to substantially loosen their contacts with the pockets (Figure 5; Table 1). Lipids could move under tension and adopt a more horizontal orientation with respect to the membrane plane in the WT channel (Figure S10). The addition of molecules that could compete for the pockets may support or disrupt lipids acting as negative allosteric modulators within the pocket sites (Figure 7C). Following similar modifications in MscL that restrict lipid access to the pockets, or when molecules that specifically bind these pockets (and compete with lipids) were introduced, there was a reduction in the activation threshold of MscL, prolonged channel openings, and an increase in the open channel probability (Iscla et al., 2014; Kapsalis et al., 2019; Wray et al., 2019).

Disruption of lipid access into the pockets of TbMscL promoted a mechanical response in the absence of applied tension, while subtle structural differences between these pockets in MscL orthologs dramatically altered their function (Kapsalis et al., 2019, 2020). This pocket region in TbMscL is important for lipid binding (Powl et al., 2005) and binds specific molecules in *Escherichia coli* MscL (EcMscL), which could modulate its activity (Wray et al., 2016, 2019).

Our model is consistent with the notion that other modifications or sites near or within the pocket region could be stabilizing the MS state(s) by disrupting lipid access to the pockets even more effectively than the L89W modification. TbMscL L89 is located at the entrance of the pockets and is structurally aligned to EcMscL M94 and in close proximity to I92, F93, I96, and K97, modifications of which influence the activation of MscL by ultrasound pulses (Ye et al., 2018) and decrease its tension threshold for use in a multicompartmental lipid vesicle framework (Hindley et al., 2019). Molecules specific to MscL are expected to act through the disruption of lipid-protein interactions (Bavi et al., 2016) within these pockets. These did not fully open the channel, but caused a structural rearrangement of the cytoplasmic side of TM2 accompanied by stretching of the cytoplasmic loop (Wray et al., 2016, 2019). This is in agreement with the structural changes observed here by HDX and ESEEM and previously shown by PELDOR (Ackermann et al., 2017; Kapsalis et al., 2019, 2020) for the modified L89W TbMscL channel (or the structurally equivalent M94 EcMscL) and the tension-activated WT channel (Figures 1, 3, 4, 7, and S8).

Transition to the intermediate state increased solvent accessibility on the upper TM1 and TM2, the cytoplasmic loops, and the top portion of the cytoplasmic helical bundle (Figures 1 and 2), which is consistent with the stretching of the cytoplasmic loops, the shortening of which influences the gating properties, pore conductance, and oligomeric assembly of MscL (Herrera et al., 2018; Reading et al., 2015; Wray et al., 2016; Yang et al., 2012).

The top of TM1 (residues 36–41) partially unfolds within a single subunit of the MscL pentamer, while the remaining 4 subunits bend without fully converting into a loop. This finding was consistently observed across all 3 MD simulation repeats following the symmetric application of tension along the membrane plane and suggests the presence of subunit asymmetry during MscL opening (Birkner et al., 2012; Konijnenberg et al., 2014; Mika et al., 2013). In contrast to the WT channel, all 5 TM1 helices bend without a break in helical symmetry or conversion into a loop in the L89W channel (Figure 4). For the top of the TM1 helix to partially unfold, lipids should become loosely associated with the pockets before asymmetric opening can occur and eventually lead to full channel opening. Whether the lipids are sequentially released (or loosen their contact) following single subunit disengagement remains to be elucidated. Shifting of the equilibrium occurs to destabilize the closed state and initiate symmetric (simultaneous) or asymmetric (sequential) pocket lipid removal from the 5 MscL subunits. Our data are compatible with such an asymmetry in opening, and this structural effect may be the consequence of lipids moving out from the 5 pockets of MscL sequentially (Mika et al., 2013). Subsequently, following initial expansion and conversion into loops, these loops could then provide membrane handles for MscL to transit to its full opening state (Videos S1 and S2).

Despite a significant membrane thinning (~1.2 nm), the pore of L89W does not allow water molecules to flow through (Figures 4 and 5). Membrane thickness and hydrophobic mismatch energetically contribute to the gating of MscL, but our data suggest that they are not the sole driving force, and pocket lipids must be released first to destabilize the closed state of MscL. However, it should be noted that while tension application and channel gating occur within the 300 ns that our simulations run, this may be faster compared to the timescale of MscL opening transitions *in situ*.

Lipids must adopt certain angles in regard to the membrane plane to enter (or exit) the pockets, and these angles should be consistent with lipids oriented horizontally, contrary to the bulk bilayer lipids. Such lipids have been resolved in multiple X-ray and cryo-EM structures of MscS (Pliotas et al., 2015; Rasmussen et al., 2019; Zhang et al., 2021), YnaI (Flegler et al., 2020), MSL1 (Deng et al., 2020b), TRAAK (Brohawn et al., 2014), TREK-2 (Dong et al., 2015), and TRPV3 (Deng et al., 2020a) channels. When we calculated the lipid order parameters for our MD simulations, we found that under tension application, the lipids adopt a substantially more horizontal orientation (Figure S10). This is consistent with significantly lower pulling forces required to remove single lipids from the pockets of TbMscL when they are applied at 45° with respect to the membrane plane (Vanegas and Arroyo, 2014).

In conclusion, here, we have generated tension- and modification-activated MscL states, and interrogated their pore hydration properties and lipid-protein interactions, and monitored their structural dynamics by experimental (HDX-MS and 3pESEEM solvent accessibility measurements) and computational (MD simulations) tools. Accumulatively, our data demonstrate that the intermediate expanded MscL state derived by pocket delipidation (L89W) is structurally analogous to the tension-activated MscL state (mechanical) (Figure 7C). Our data further suggest that the expanded MscL state is the final destination of the pocket lipid removal activation path of MscL, but only an intermediate stop on the tension-mediated activation path, which leads to a fully open MscL state. It is quite intriguing that whether pocket lipid depletion is caused by tension application or modification, we find that MscL is sampling similar distinctive states, as is also the case for the structurally unrelated MscS, whose states are also guided by pocket lipid availability (Flegler et al., 2021; Zhang et al., 2021).

The structural similarities of these two differently derived states suggest that lipids could act as molecular triggers on pressure-sensitive domains and mimic the naturally occurring tension activation in MS channels, with implications for MS channel evolution and multimodality.

STAR METHODS

Detailed methods are provided in the online version of this paper and include the following:

- KEY RESOURCES TABLE
- RESOURCE AVAILABILITY
  - Lead contact
  - Materials availability
  - HDX mass spectrometry

- 3pESEEM and MD
- Data and code availability
- EXPERIMENTAL MODEL AND SUBJECT
- METHOD DETAILS
  - Mutagenesis and protein expression
  - Protein purification and spin labeling
  - 3pESEEM sample preparation
  - 3pESEEM spectroscopy
  - HDX-MS sample preparation
  - HDX and LC-MS/MS
  - Atomistic molecular dynamics simulations under no tension
  - Atomistic molecular dynamics simulations under bilayer tension
- QUANTIFICATION AND STATISTICAL ANALYSIS

SUPPLEMENTAL INFORMATION

Supplemental information can be found online at https://doi.org/10.1016/j.str.2021.12.004.

ACKNOWLEDGMENTS

This project was supported by a Biotechnology and Biological Sciences Research Council (BBSRC) grant (BB/S018069/1) to C.P., who also acknowledges support from the Wellcome Trust (WT) (219999/Z/19/Z) and the Chinese Scholarship Council (CSC) in the form of studentships for B.J.L. and B.W. respectively. A.N.C. is a Sir Henry Dale Fellow jointly funded by the WT and the Royal Society (220628/Z/20/Z). Funding from the BBSRC (BB/M012573/1) enabled the purchase of mass spectrometry equipment.

AUTHOR CONTRIBUTIONS

C.P. conceived and designed the project. B.W., B.J.L., C.K., J.R.A., H.E.M., A.C.K., and C.P. performed the research. B.W., B.J.L., C.K., J.R.A., F.S., H.E.M., A.N.C., A.C.K., and C.P. processed and analyzed the data. C.P. wrote the manuscript, assisted by B.W. and B.J.L., with contributions from H.E.M., A.N.C., and A.C.K.

DECLARATION OF INTERESTS

The authors declare no competing interests.

Received: September 9, 2021
Revised: October 13, 2021
Accepted: December 7, 2021
Published: January 4, 2022

REFERENCES

Abraham, M.J., Murtola, T., Schulz, R., Páll, S., Smith, J.C., Hess, B., Lindahl, E., and Lindahl, E. (2015). GROMACS: high performance molecular simulations through multi-level parallelism from laptops to supercomputers. SoftwareX *1-2*, 19–25.

Ackermann, K., Pliotas, C., Valera, S., Naismith, J.H., and Bode, B.E. (2017). Sparse Labeling PELDOR Spectroscopy on Multimeric Mechanosensitive Membrane Channels. Biophys J. *113*, 1968–1978.

Ahdash, Z., Pyle, E., Allen, W.J., Corey, R.A., Collinson, I., and Politis, A. (2019). HDX-MS reveals nucleotide-dependent, anti-correlated opening and closure of SecA and SecY channels of the bacterial translocon. Elife *8*, e47402. https://doi.org/10.7554/eLife.474022.

Anishkin, A., Akitake, B., and Sukharev, S. (2008). Characterization of the resting MscS: modeling and analysis of the closed bacterial mechanosensitive channel of small conductance. Biophys. J. *94*, 1252–1266.

Anishkin, A., Chiang, C.S., and Sukharev, S. (2005). Gain-of-function mutations reveal expanded intermediate states and a sequential action of two gates in MscL. J. Gen. Physiol. *125*, 155–170.

Aryal, P., Jarerattanachat, V., Clausen, M.V., Schewe, M., McClenaghan, C., Argent, L., Conrad, L.J., Dong, Y.Y., Pike, A.C.W., Carpenter, E.P., et al. (2017). Bilayer-mediated structural transitions control mechanosensitivity of the TREK-2 K2P channel. Structure *25*, 708–718.e2.

Bavi, N., Bavi, O., Vossoughi, M., Naghdabadi, R., Hill, A.P., Martinac, B., and Jamali, Y. (2017a). Nanomechanical properties of MscL alpha helices: a steered molecular dynamics study. Channels (Austin) *11*, 209–223.

Bavi, N., Cortes, D.M., Cox, C.D., Rohde, P.R., Liu, W., Deitmer, J.W., Bavi, O., Strop, P., Hill, A.P., Rees, D., et al. (2016). The role of MscL amphipathic N terminus indicates a blueprint for bilayer-mediated gating of mechanosensitive channels. Nat. Commun. *7*, 11984.

Bavi, N., Martinac, A.D., Cortes, D.M., Bavi, O., Ridone, P., Nomura, T., Hill, A.P., Martinac, B., and Perozo, E. (2017b). Structural dynamics of the MscL C-terminal domain. Sci. Rep. *7*, 17229.

Belyy, V., Kamaraju, K., Akitake, B., Anishkin, A., and Sukharev, S. (2010). Adaptive behavior of bacterial mechanosensitive channels is coupled to membrane mechanics. J. Gen. Physiol. *135*, 641–652.

Betanzos, M., Chiang, C.S., Guy, H.R., and Sukharev, S. (2002). A large iris-like expansion of a mechanosensitive channel protein induced by membrane tension. Nat. Struct. Biol. *9*, 704–710.

Birkner, J.P., Poolman, B., and Kocer, A. (2012). Hydrophobic gating of mechanosensitive channel of large conductance evidenced by single-subunit resolution. Proc. Natl. Acad. Sci. U S A *109*, 12944–12949.

Boucher, P.A., Morris, C.E., and Joos, B. (2009). Mechanosensitive closed-closed transitions in large membrane proteins: osmoprotection and tension damping. Biophys. J. *97*, 2761–2770.

Brohawn, S.G. (2015). How ion channels sense mechanical force: insights from mechanosensitive K2P channels TRAAK, TREK1, and TREK2. Ann. N. Y. Acad. Sci. *1352*, 20–32.

Brohawn, S.G., Campbell, E.B., and MacKinnon, R. (2014). Physical mechanism for gating and mechanosensitivity of the human TRAAK K+ channel. Nature *516*, 126–130.

Chang, G., Spencer, R.H., Lee, A.T., Barclay, M.T., and Rees, D.C. (1998). Structure of the MscL homolog from Mycobacterium tuberculosis: a gated mechanosensitive ion channel. Science *282*, 2220–2226.

Cieslak, J.A., Focia, P.J., and Gross, A. (2010). Electron spin-echo envelope modulation (ESEEM) reveals water and phosphate interactions with the KcsA potassium channel. Biochemistry *49*, 1486–1494.

Cornwell, O., Radford, S.E., Ashcroft, A.E., and Ault, J.R. (2018). Comparing hydrogen deuterium exchange and Fast photochemical oxidation of proteins: a structural characterisation of wild-type and DeltaN6 beta2-microglobulin. J. Am. Soc. Mass Spectrom. *29*, 2413–2426.

Cox, C.D., Nakayama, Y., Nomura, T., and Martinac, B. (2015). The evolutionary 'tinkering' of MscS-like channels: generation of structural and functional diversity. Pflugers Arch. *467*, 3–13.

Cox, C.D., Zhang, Y., Zhou, Z., Walz, T., and Martinac, B. (2021). Cyclodextrins increase membrane tension and are universal activators of mechanosensitive channels. Proc Natl Acad Sci U S A *118*, e2104820118.

Cryar, A., Groves, K., and Quaglia, M. (2017). Online hydrogen-deuterium exchange traveling wave ion mobility mass spectrometry (HDX-IM-MS): a systematic evaluation. J. Am. Soc. Mass *28*, 1192–1202.

Darden, T., York, D., and Pedersen, L. (1993). Particle mesh Ewald: an N·log(N) method for Ewald sums in large systems. J. Chem. Phys. *98*, 10089–10092.

Delano, W.L. (2002). The PyMOL Molecular Graphics System (Delano Scientific).

Deng, Z., Maksaev, G., Rau, M., Xie, Z., Hu, H., Fitzpatrick, J.A.J., and Yuan, P. (2020a). Gating of human TRPV3 in a lipid bilayer. Nat. Struct. Mol. Biol. *27*, 635–644.

Deng, Z., Maksaev, G., Schlegel, A.M., Zhang, J., Rau, M., Fitzpatrick, J.A.J., Haswell, E.S., and Yuan, P. (2020b). Structural mechanism for gating of a

CellPress
OPEN ACCESS

eukaryotic mechanosensitive channel of small conductance. Nat. Commun. *11*, 3690.

Dong, Y.Y., Pike, A.C., Mackenzie, A., McClenaghan, C., Aryal, P., Dong, L., Quigley, A., Grieben, M., Goubin, S., Mukhopadhyay, S., et al. (2015). K2P channel gating mechanisms revealed by structures of TREK-2 and a complex with Prozac. Science *347*, 1256–1259.

Erdogmus, S., Storch, U., Danner, L., Becker, J., Winter, M., Ziegler, N., Wirth, A., Offermanns, S., Hoffmann, C., Gudermann, T., et al. (2019). Helix 8 is the essential structural motif of mechanosensitive GPCRs. Nat. Commun. *10*, 5784.

Evans, D.J., and Holian, B.L. (1985). The Nose-Hoover thermostat. J. Chem. Phys. *83*, 4069–4074.

Flegler, V.J., Rasmussen, A., Borbil, K., Boten, L., Chen, H.A., Deinlein, H., Halang, J., Hellmanzik, K., Loffler, J., Schmidt, V., et al. (2021). Mechanosensitive channel gating by delipidation. Proc. Natl. Acad. Sci. U S A *118*, e2107095118. https://doi.org/10.1073/pnas.2107095118.3.

Flegler, V.J., Rasmussen, A., Rao, S., Wu, N., Zenobi, R., Sansom, M.S.P., Hedrich, R., Rasmussen, T., and Bottcher, B. (2020). The MscS-like channel Ynal has a gating mechanism based on flexible pore helices. Proc. Natl. Acad. Sci. U S A *117*, 28754–28762.

Gullingsrud, J., and Schulten, K. (2003). Gating of MscL studied by steered molecular dynamics. Biophys. J. *85*, 2087–2099.

Hartley, A.M., Ma, Y., Lane, B.J., Wang, B., and Pliotas, C. (2021). Using pulsed EPR in the structural analysis of integral membrane proteins. Electron Paramag Reson. *27*, 74–108.

Herrera, N., Maksaev, G., Haswell, E.S., and Rees, D.C. (2018). Elucidating a role for the cytoplasmic domain in the Mycobacterium tuberculosis mechanosensitive channel of large conductance. Sci. Rep. *8*, 14566.

Hess, B., Bekker, H., Berendsen, H.J.C., and Fraaije, J.G.E.M. (1997). INCS: a linear constraint solver for molecular simulations. J. Comput. Chem. *18*, 1463–1472.

Hindley, J.W., Zheleva, D.G., Elani, Y., Charalambous, K., Barter, L.M.C., Booth, P.J., Bevan, C.L., Law, R.V., and Ces, O. (2019). Building a synthetic mechanosensitive signaling pathway in compartmentalized artificial cells. Proc. Natl. Acad. Sci. U S A *116*, 16711–16716.

Humphrey, W., Dalke, A., and Schulten, K. (1996). VMD: visual molecular dynamics. J. Mol. Graph. *14*, 33–38, 27–38.

Iscla, I., Wray, R., Eaton, C., and Blount, P. (2015). Scanning MscL channels with targeted post-translational modifications for functional alterations. PLoS One *10*, e0137994.

Iscla, I., Wray, R., Wei, S., Posner, B., and Blount, P. (2014). Streptomycin potency is dependent on MscL channel expression. Nat. Commun. *5*, 4891.

Jeon, J., and Voth, G.A. (2008). Gating of the mechanosensitive channel protein MscL: the interplay of membrane and protein. Biophys. J. *94*, 3497–3511.

Jo, S., Kim, T., Iyer, V.G., and Im, W. (2008). CHARMM-GUI: a web-based graphical user interface for CHARMM. J. Comput. Chem. *29*, 1859–1865.

Kapsalis, C., Ma, Y., Bode, B.E., and Pliotas, C. (2020). In-lipid structure of pressure-sensitive domains hints mechanosensitive channel functional diversity. Biophys. J. *119*, 448–459.

Kapsalis, C., Wang, B., El Mkami, H., Pitt, S.J., Schnell, J.R., Smith, T.K., Lippiat, J.D., Bode, B.E., and Pliotas, C. (2019). Allosteric activation of an ion channel triggered by modification of mechanosensitive nano-pockets. Nat. Commun. *10*, 4619.

Katsuta, H., Sawada, Y., and Sokabe, M. (2019). Biophysical mechanisms of membrane-thickness-dependent MscL gating: an all-atom molecular dynamics study. Langmuir *35*, 7432–7442.

Kefauver, J.M., Ward, A.B., and Patapoutian, A. (2020). Discoveries in structure and physiology of mechanically activated ion channels. Nature *587*, 567–576.

Klesse, G., Rao, S., Sansom, M.S.P., and Tucker, S.J. (2019). CHAP: a versatile tool for the structural and functional annotation of ion channel pores. J. Mol. Biol. *431*, 3353–3365.

Konermann, L., Pan, J., and Liu, Y.H. (2011). Hydrogen exchange mass spectrometry for studying protein structure and dynamics. Chem. Soc. Rev. *40*, 1224–1234.

Konijnenberg, A., Yilmaz, D., Ingolfsson, H.I., Dimitrova, A., Marrink, S.J., Li, Z., Venien-Bryan, C., Sobott, F., and Kocer, A. (2014). Global structural changes of an ion channel during its gating are followed by ion mobility mass spectrometry. Proc. Natl. Acad. Sci. U S A *111*, 17170–17175.

Laganowsky, A., Reading, E., Allison, T.M., Ulmschneider, M.B., Degiacomi, M.T., Baldwin, A.J., and Robinson, C.V. (2014). Membrane proteins bind lipids selectively to modulate their structure and function. Nature *510*, 172–175.

Lau, A.M.C., Ahdash, Z., Martens, C., and Politis, A. (2019). Deuteros: software for rapid analysis and visualization of data from differential hydrogen deuterium exchange-mass spectrometry. Bioinformatics *35*, 3171–3173.

Lee, S., Tran, A., Allsopp, M., Lim, J.B., Henin, J., and Klauda, J.B. (2014). CHARMM36 united atom chain model for lipids and surfactants. J. Phys. Chem. B *118*, 547–556.

Li, J., Guo, J., Ou, X., Zhang, M., Li, Y., and Liu, Z. (2015). Mechanical coupling of the multiple structural elements of the large-conductance mechanosensitive channel during expansion. Proc. Natl. Acad. Sci. U S A *112*, 10726–10731.

Liu, L., Hess, J., Sahu, I.D., FitzGerald, P.G., McCarrick, R.M., and Lorigan, G.A. (2016). Probing the local secondary structure of human vimentin with electron spin echo envelope modulation (ESEEM) spectroscopy. J. Phys. Chem. B *120*, 12321–12326.

Malcolm, H.R., Blount, P., and Maurer, J.A. (2015). The mechanosensitive channel of small conductance (MscS) functions as a Jack-in-the box. Biochim. Biophys. Acta *1848*, 159–166.

Martens, C., Shekhar, M., Lau, A.M., Tajkhorshid, E., and Politis, A. (2019). Integrating hydrogen-deuterium exchange mass spectrometry with molecular dynamics simulations to probe lipid-modulated conformational changes in membrane proteins. Nat. Protoc. *14*, 3183–3204.

Martinac, A.D., Bavi, N., Bavi, O., and Martinac, B. (2017). Pulling MscL open via N-terminal and TM1 helices: a computational study towards engineering an MscL nanovalve. PLoS One *12*, e0183822.

Matalon, E., Kaminker, I., Zimmermann, H., Eisenstein, M., Shai, Y., and Goldfarb, D. (2013). Topology of the trans-membrane peptide WALP23 in model membranes under negative mismatch conditions. J. Phys. Chem. B *117*, 2280–2293.

Michou, M., Kapsalis, C., Pliotas, C., and Skretas, G. (2019). Optimization of recombinant membrane protein production in the engineered Escherichia coli strains SuptoxD and SuptoxR. ACS Synth. Biol. *8*, 1631–1641.

Mika, J.T., Birkner, J.P., Poolman, B., and Kocer, A. (2013). On the role of individual subunits in MscL gating: "all for one, one for all? FASEB J. *27*, 882–892.

Moller, I.R., Slivacka, M., Nielsen, A.K., Rasmussen, S.G.F., Gether, U., Loland, C.J., and Rand, K.D. (2019). Conformational dynamics of the human serotonin transporter during substrate and drug binding. Nat. Commun. *10*, 1687.

Mukherjee, N., Jose, M.D., Birkner, J.P., Walko, M., Ingolfsson, H.I., Dimitrova, A., Arnarez, C., Marrink, S.J., and Kocer, A. (2014). The activation mode of the mechanosensitive ion channel, MscL, by lysophosphatidylcholine differs from tension-induced gating. FASEB J. *28*, 4292–4302.

Naismith, J.H., and Booth, I.R. (2012). Bacterial mechanosensitive channels–MscS: evolution's solution to creating sensitivity in function. Annu. Rev. Biophys. *41*, 157–177.

Parrinello, M., and Rahman, A. (1981). Polymorphic transitions in single crystals: a new molecular dynamics method. J. Appl. Phys. *52*, 7182–7190.

Patrick, J.W., Boone, C.D., Liu, W., Conover, G.M., Liu, Y., Cong, X., and Laganowsky, A. (2018). Allostery revealed within lipid binding events to membrane proteins. Proc. Natl. Acad. Sci. U S A *115*, 2976–2981.

Perozo, E., Kloda, A., Cortes, D.M., and Martinac, B. (2002). Physical principles underlying the transduction of bilayer deformation forces during mechanosensitive channel gating. Nat. Struct. Biol. *9*, 696–703.

Pettersen, E.F., Goddard, T.D., Huang, C.C., Couch, G.S., Greenblatt, D.M., Meng, E.C., and Ferrin, T.E. (2004). UCSF Chimera–a visualization system for exploratory research and analysis. J. Comput. Chem. *25*, 1605–1612.

Pliotas, C. (2017). Ion channel conformation and oligomerization assessment by site-directed spin labeling and pulsed-EPR. Methods Enzymol. *594*, 203–242.

Pliotas, C., Dahl, A.C., Rasmussen, T., Mahendran, K.R., Smith, T.K., Marius, P., Gault, J., Banda, T., Rasmussen, A., Miller, S., et al. (2015). The role of lipids in mechanosensation. Nat. Struct. Mol. Biol. *22*, 991–998.

Pliotas, C., and Naismith, J.H. (2016). Spectator no more, the role of the membrane in regulating ion channel function. Curr. Opin. Struct. Biol. *45*, 59–66.

Pliotas, C., Ward, R., Branigan, E., Rasmussen, A., Hagelueken, G., Huang, H., Black, S.S., Booth, I.R., Schiemann, O., and Naismith, J.H. (2012). Conformational state of the MscS mechanosensitive channel in solution revealed by pulsed electron-electron double resonance (PELDOR) spectroscopy. Proc. Natl. Acad. Sci. U S A *109*, E2675–E2682.

Powl, A.M., East, J.M., and Lee, A.G. (2005). Heterogeneity in the binding of lipid molecules to the surface of a membrane protein: hot spots for anionic lipids on the mechanosensitive channel of large conductance MscL and effects on conformation. Biochemistry *44*, 5873–5883.

Rasmussen, T., Flegler, V.J., Rasmussen, A., and Bottcher, B. (2019). Structure of the mechanosensitive channel MscS embedded in the membrane bilayer. J. Mol. Biol. *431*, 3081–3090. https://doi.org/10.1016/j.jmb.2019.07.006.

Reading, E., Walton, T.A., Liko, I., Marty, M.T., Laganowsky, A., Rees, D.C., and Robinson, C.V. (2015). The effect of detergent, temperature, and lipid on the oligomeric state of MscL constructs: insights from mass spectrometry. Chem. Biol. *22*, 593–603.

Reddy, B., Bavi, N., Lu, A., Park, Y., and Perozo, E. (2019). Molecular basis of force-from-lipids gating in the mechanosensitive channel MscS. Elife *8*, e50486. https://doi.org/10.7554/eLife.50486.

Ridone, P., Grage, S.L., Patkunarajah, A., Battle, A.R., Ulrich, A.S., and Martinac, B. (2018). "Force-from-lipids" gating of mechanosensitive channels modulated by PUFAs. J. Mech. Behav. Biomed. Mater. *79*, 158–167.

Smart, O.S., Goodfellow, J.M., and Wallace, B.A. (1993). The pore dimensions of gramicidin A. Biophys. J. *65*, 2455–2460.

Teng, J., Loukin, S., Anishkin, A., and Kung, C. (2015). The force-from-lipid (FFL) principle of mechanosensitivity, at large and in elements. Pflugers Arch. *467*, 27–37.

Vanegas, J.M., and Arroyo, M. (2014). Force transduction and lipid binding in MscL: a continuum-molecular approach. PLoS One *9*, e113947.

Volkov, A., Dockter, C., Bund, T., Paulsen, H., and Jeschke, G. (2009). Pulsed EPR determination of water accessibility to spin-labeled amino acid residues in LHCIIb. Biophys. J. *96*, 1124–1141.

Ward, R., Pliotas, C., Branigan, E., Hacker, C., Rasmussen, A., Hagelueken, G., Booth, I.R., Miller, S., Lucocq, J., Naismith, J.H., et al. (2014). Probing the structure of the mechanosensitive channel of small conductance in lipid bilayers with pulsed electron-electron double resonance. Biophys. J. *106*, 834–842.

Wray, R., Iscla, I., Gao, Y., Li, H., Wang, J., and Blount, P. (2016). Dihydrostreptomycin directly binds to, modulates, and passes through the MscL channel pore. PLoS Biol. *14*, e1002473.

Wray, R., Iscla, I., Kovacs, Z., Wang, J., and Blount, P. (2019). Novel compounds that specifically bind and modulate MscL: insights into channel gating mechanisms. FASEB J. *33*, 3180–3189.

Yang, L.M., Wray, R., Parker, J., Wilson, D., Duran, R.S., and Blount, P. (2012). Three routes to modulate the pore size of the MscL channel/nanovalve. ACS Nano *6*, 1134–1141.

Ye, J., Tang, S., Meng, L., Li, X., Wen, X., Chen, S., Niu, L., Li, X., Qiu, W., Hu, H., et al. (2018). Ultrasonic control of neural activity through activation of the mechanosensitive channel MscL. Nano Lett. *18*, 4148–4155.

Zhang, R., Sahu, I.D., Gibson, K.R., Muhammad, N.B., Bali, A.P., Comer, R.G., Liu, L., Craig, A.F., McCarrick, R.M., Dabney-Smith, C., et al. (2015). Development of electron spin echo envelope modulation spectroscopy to probe the secondary structure of recombinant membrane proteins in a lipid bilayer. Protein Sci. *24*, 1707–1713.

Zhang, Y., Daday, C., Gu, R.X., Cox, C.D., Martinac, B., de Groot, B.L., and Walz, T. (2021). Visualization of the mechanosensitive ion channel MscS under membrane tension. Nature *590*, 509–514.

STAR METHODS

KEY RESOURCES TABLE

REAGENT or RESOURCE	SOURCE	IDENTIFIER
**Bacterial and virus strains**		
BL21 (DE3) competent cells	ThermoFisher	Cat# EC0114
**Chemicals, peptides, and recombinant proteins**		
N-Dodecyl-b-D-Maltopyranoside (DDM), anagrade	Anatrace or Glycon	Cat# D310 or D97002
S-(2,2,5,5-tetramethyl-2,5-dihydro-1H-pyrrol-3-yl)methyl methanesulfonothioate (MTSSL)	Santa Cruz or Toronto Research Chemicals	Cat# 81213-52-7 or O875000
**Critical commercial assays**		
Ni-NTA agarose resin	Invitrogen	Cat# R901-15
Superdex 200 increase 10/300 GL column	Cytiva	Cat# 28-9909-44
**Deposited data**		
MscL and MscL L89W HDX mass spectrometry data	This Paper	ProteomeXchange PXD021983
MscL and MscL L89W MD simulation data	This Paper	http://archive.researchdata.leeds.ac.uk/777/
MscL (various) mutants ESEEM data	This Paper	http://archive.researchdata.leeds.ac.uk/777/
Atomic coordinates and structural factors (MtMscL)	Chang et al., 1998	PDB: 2OAR
Atomic coordinates and structural factors (MaMscL)	Li et al. (2015)	PDB: 4Y7J
**Oligonucleotides**		
Primer: MscL N13C forward CGCGGTTGTATTGTTGACTTGGCGGT	This Paper	NA
Primer: MscL N13C reverse CAACAATACAACCGCGAGCCAGGAATTC	This Paper	N/A
Primer: MscL L17C forward TGACTGCGCGGTTGCGGTTGTCATTGG	This Paper	N/A
Primer: MscL L17C reverse CCGCGCAGTCAACAATATTA CCGCGAGC	This Paper	N/A
Primer: MscL V21C forward TTGCGTGTGTCATTGGTAC CGCGTTTACCG	This Paper	N/A
Primer: MscL V21C reverse CAATGACACACGCAACC GCCAAGTCAACAAT	This Paper	N/A
Primer: MscL V71C forward GATTTGAATTGCCTGCTG AGCGCCGCTATTAAC	This Paper	N/A
Primer: MscL V71C reverse GCAGGCAATTCAAATCG ATGGTCTGACCACC	This Paper	N/A
Primer: MscL S74C forward CTGCTGTGCGCCGCTATTAACTTC	This Paper	N/A
Primer: MscL S74C reverse GGCGCACAGCAGGACATTCAAATC	This Paper	N/A

(*Continued on next page*)

Continued		
REAGENT or RESOURCE	SOURCE	IDENTIFIER
Primers: Remaining primers have been reported	Kapsalis et al. (2019)	N/A
Recombinant DNA		
Plasmid: pJ411:140126-TbMscL	Kapsalis et al. (2019)	N/A
Software and algorithms		
PLGS (v3.0.2)	Waters	https://www.waters.com/waters/en_GB/ProteinLynx-Global-SERVER-(PLGS)/nav.htm?locale=en_GB&cid=513821
DynamX (v3.0.0)	Waters	https://www.waters.com/waters/library.htm?locale=en_US&lid=134832928
PAVED	Cornwell et al. (2018)	N/A
Deuteros	Lau et al. (2019)	https://github.com/andymlau/Deuteros_2.0
Pymol	(Delano, 2002)	https://pymol.org/
UCSF chimera	(Pettersen et al., 2004)	https://www.cgl.ucsf.edu/chimera/
VMD	(Humphrey et al., 1996)	https://www.ks.uiuc.edu/Research/vmd/
CHAP	Klesse et al. (2019)	https://www.channotation.org/
HOLE	Smart et al. (1993)	http://www.holeprogram.org/
CHARMM-GUI	Jo et al. (2008)	https://www.charmm-gui.org/
GROMACS_2016.4	Abraham et al., (2015)	http://www.gromacs.org/
MATLAB curve fitting toolbox	NA	https://uk.mathworks.com/products/curvefitting.html
Xepr software	Bruker	https://www.bruker.com/en/products-and-solutions/mr/epr-instruments/epr-software.html
Other		
Vivaspin-2 (100 kDa MWCO) concentrator	Sartorius	Cat# VS0241
Bio-beads SM-2 adsorbents	Bio-Rad	Cat# 1523920

RESOURCE AVAILABILITY

Lead contact

Further information and requests for resources and reagents should be directed to and will be fulfilled by the lead contact, Christos Pliotas (c.pliotas@leeds.ac.uk).

Materials availability

All unique reagents generated in this study are available from the lead contact upon reasonable request.

HDX mass spectrometry

HDX-MS data have been deposited to the ProteomeXchange Consortium via the PRIDE partner repository with the dataset identifier PXD021983.

3pESEEM and MD

Data are available within the following link: http://archive.researchdata.leeds.ac.uk/777/

This paper does not report original code. Any additional information required to reanalyze the data reported is available from the lead contact upon request.

Data and code availability

HDX mass spectrometry: HDX-MS data have been deposited to the ProteomeXchange Consortium via the PRIDE partner repository with the dataset identifier PXD021983.

3pESEEM and MD: Data are available within the following link: http://archive.researchdata.leeds.ac.uk/777/ and *via* the DOI: https://doi.org/10.5518/914.

This paper does not report original code. Any additional information required to reanalyze the data reported is available from the lead contact upon request.

EXPERIMENTAL MODEL AND SUBJECT

Plasmid propagation was performed using DH5α competent *E. coli* cells plated on Luria Broth (LB) agar (37°C) or inoculated in LB liquid media (37°C, 200 RPM); both grown overnight in the presence of a selective antibiotic. Recombinant MscL and associated mutants were produced in *E. coli* BL21(DE3) cells (Thermo Fisher), cultured in LB Broth supplemented with kanamycin (50 μg mL−1) for 4 hrs (25°C, 200 RPM) following IPTG induction (final concentration 1 mM) at an OD600 ~0.8.

METHOD DETAILS

Mutagenesis and protein expression

Mutagenesis of TbMscL with a C-terminal 6xHis-tag in a pJ411:140126 vector allowed the generation of single and double mutants. Primers were used for site-directed mutagenesis reactions to introduce the point mutations. The wild type and mutant plasmids were transformed into BL21(DE3) (ThermoFisher) E. coli cells. Cells were grown in 550 m LB medium in a 2L flask at 37°C until they reached an OD600 ~0.8 and subsequently cooled down to 25°C and induced with 1 mM IPTG for 3.5 hrs. Cells were harvested by centrifugation at 4000 × g and stored at - 80°C, until further use.

Protein purification and spin labeling

The protocols followed in this study were similar to the ones previously described (Kapsalis et al., 2019, 2020; Pliotas, 2017). In brief, following protein expression, cell pellets were resuspended in phosphate-buffered saline and subjected to lysis using a cell disrupter at 30 kpsi. To remove cell debris the suspension was centrifuged at 4000 × g for 20 min and the resulting supernatant was centrifuged again at 100,000 × g for 1 h. The membrane pellet was resuspended and solubilized in buffer containing 50 mM sodium phosphate at pH 7.5, 300 mM NaCl, 10% v/v glycerol, 50 mM imidazole and 1.5% w/v DDM (solubilization buffer) and incubated at 4°C for 1 h. The sample was then centrifuged at 4000 × g for 20 min and the supernatant was passed through a $^{+2}$Ni-NTA column containing 0.75 mL of $^{+2}$Ni-NTA beads. The column was then washed with 10 mL buffer containing 50 mM sodium phosphate of pH 7.5, 300 mM NaCl, 10% v/v glycerol and 0.05% w/v DDM (wash buffer) and then with 5 mL wash buffer supplemented with 3 mM TCEP, for reduction of the cysteines. Afterward, MTSSL dissolved in wash buffer at a 10-fold excess of the expected protein concentration was added to the column and left to react for 2 h at 4°C. The protein was then eluted from the column with 5 mL of wash buffer supplemented with 300 mM imidazole. Finally, the protein was subjected to SEC using a Superdex 200 column (GE Healthcare) equilibrated with buffer containing 50 mM sodium phosphate of pH 7.5, 300 mM NaCl and 0.05% w/v DDM. Collected fractions of TbMscL were then concentrated to ~800 μM monomer concentration, which is suitable for the EPR samples preparation.

3pESEEM sample preparation

Purified TbMscL detergent samples were diluted by 50% with deuterated ethylene glycol as cryoprotectant to a final monomer concentration of ~400 μM and a volume of 70 μL of the mixture was then transferred into a 3 mm (OD) quartz tubes and flash-frozen in liquid N_2.

3pESEEM spectroscopy

X-band three pulse ESEEM measurements were performed on a Bruker ELEXSYS E580 spectrometer with a 4 mm dielectric resonator (MD4). Measurements were conducted at 80 K. The 3-pulse sequence used for the experiments is π/2-τ-π/2-T-π/2-stimulated echo with a pulse length $t_{\pi/2}$ = 16 ns and inter-pulse delay τ that was adjusted to match either the blind spots of the proton or deuterium. The delay T was incremented from 400ns in 12 ns steps. A four-step phase cycling was used to eliminate the unwanted echoes. All the measurements were performed at the maximum of the field sweep spectrum of the nitroxide. For solvent accessibility determination, only -3pESEEM data recorded with τ that corresponds to the proton blind spot were used. The obtained time-domain traces were background-corrected, apodized with a hamming window and zero-filled prior to Fourier transformation. The solvent accessibility can be determined by different analysis methods from the deuterium 3pESEEM. In the present study, we used a model developed by Jeschke and co-workers and it is based on deuterium modulation depth (Volkov et al., 2009). The primary ESEEM data were background corrected in such a way to obtain a deconvoluted and normalized nuclear modulation function. We used two approaches to determine the solvent accessibility; the first one by fitting the obtained nuclear modulation function for each mutant by a damped harmonic oscillation function and the outcome from the fitting was used to estimate the solvent accessibility (Volkov et al., 2009), the second approach by Fourier transformation of the nuclear modulation function and in this case the water accessibility was determined directly form the intensities of the deuterium peaks in the magnitude ESEEM spectra. The solvent accessibility parameters derived from both methods are in good agreement within the error bars as shown in Figure S3. Although the standard deviations for the fitted parameters were less than 2% for all the mutants, we additionally accounted for errors that might emanate from differences in relaxation behavior or in contribution from no-water protons between the different mutants and use an error of 5%, which has been shown to exceed all the associated experimental and fitting errors (Volkov et al., 2009). All raw ESEEM data for this study are within the following link: http://archive.researchdata.leeds.ac.uk/777/.

HDX-MS sample preparation

For HDX WT or L89W mutant MscL were not spin-labelled. The cell disruption, membrane preparation, solubilization, and application of the solubilized membranes to the $^{+2}$Ni-NTA column occurred as previously. The column was washed, as previously, with 10 mL buffer containing 50 mM sodium phosphate of pH 7.5, 300 mM NaCl, 10% v/v glycerol and 0.05% w/v DDM (wash buffer). Following this step, the protein was eluted with 5 mL of wash buffer containing 300 mM imidazole. The protein was not treated with TCEP or MTSL supplemented wash buffer as the protein was not spin-labelled. The protein was then subjected to SEC using a Superdex 200 column as previously described. The fractions were collected, and the protein samples were concentrated to achieve 350 μL of protein at 16 μM.

HDX and LC-MS/MS

HDX-MS experiments were carried out using an automated HDX robot (LEAP Technologies, Fort Lauderdale, FL, USA) coupled to an M-Class Acquity LC and HDX manager (Waters Ltd., Wilmslow, Manchester, UK). All samples were diluted to 16 μM in equilibration buffer (50 mM potassium phosphate, 300 mM NaCl, pH 7.4, 0.05% DDM) prior to analysis. 5 μL sample was added to 95 μL deuterated buffer (50 mM potassium phosphate, 300 mM NaCl pD 7.4, 0.05% DDM) and incubated at 4°C for 0.5, 1, 2, 10 or 60 min. Following the labelling reaction, samples were quenched by adding 50 μL of the labelled solution to 100 μL quench buffer (50 mM potassium phosphate, 300 mM NaCl, 0.1% DDM pH 2.2) giving a final quench pH ~2.5. 50 μL of quenched sample was injected onto the HDX Manger and passed through immobilized pepsin and aspergillopepsin columns connected in series (AffiPro, Czech Republic) at 115 μL min-1 (20°C) and loaded onto a VanGuard Pre-column Acquity UPLC BEH C18 (1.7 μm, 2.1 mm × 5 mm, Waters Ltd., Wilmslow, Manchester, UK) for 3 mins in 0.3% formic acid in water. The resulting peptides were transferred to a C18 column (75 μm × 150 mm, Waters Ltd., Wilmslow, Manchester, UK) and separated by gradient elution of 0–40% MeCN (0.1% v/v formic acid) in H2O (0.3% v/v formic acid) over 7 min at 40 μL.min^{-1}. Trapping and gradient elution of peptides was performed at 0°C to reduce back exchange. The HDX system was interfaced with a Synapt G2Si mass spectrometer (Waters Ltd., Wilmslow, Manchester, UK). HDMSE and dynamic range extension modes (Data Independent Analysis (DIA) coupled with IMS separation) were used to separate peptides prior to CID fragmentation in the transfer cell (Cryar et al., 2017). HDX data were analyzed using PLGS (v3.0.2) and DynamX (v3.0.0) software supplied with the mass spectrometer. Restrictions for identified peptides in DynamX were as follows: minimum intensity: 1000, minimum products per MS/MS spectrum: 5, minimum products per amino acid: 0.3, maximum sequence length: 25, maximum ppm error: 5, file threshold: 3/3. Following manual curation of the data, PAVED (Cornwell et al., 2018) and Deuteros (Lau et al., 2019) were used to identify peptides with statistically significant increases/decreases in deuterium uptake (applying 99% or 95% confidence intervals) and to prepare Wood's plots. The raw HDX-MS data have been deposited to the ProteomeXchange Consortium via the PRIDE partner repository with the dataset identifier PXD021983. A summary of the HDX-MS data, as recommended by reporting guidelines, is shown in Table S1. All data is available via ProteomeXchange with identifier PXD021983. Project Name: Mechanical and molecular activation lead to structurally analogous MscL states. Project accession: PXD021983 Username: reviewer_pxd021983@ebi.ac.uk, Password: lnAkk2H3.

Atomistic molecular dynamics simulations under no tension

The set up and parameters of MD simulations without tension were as previously described (Kapsalis et al., 2019). In brief, CHARMM-GUI was used to insert the TbMscL structure (2OAR) into a pre-equilibrated patch of POPC bilayer containing approximately 387 lipids and occupying an area of 120 × 120 Å2. The protein and membrane bilayer were solvated with TIP3P water and 150 mM NaCl. The simulations were performed in an NPT ensemble at 303.15 K and 1bar pressure on all xyz axes using GROMACS_2016.4 with CHARMM36 force field. The particle mesh Ewald (PME) method was applied to calculate electrostatic forces, and the van der Waals interactions were smoothly switched off at 10–12 Å by the force-switch manner. The time step was set to 2 fs in conjunction with the LINCS algorithm. After the standard minimization and equilibration steps using the GROMACS input scripts generated by CHARMM-GUI, 100 ns dynamic simulation was calculated.

Atomistic molecular dynamics simulations under bilayer tension

These MD simulations were also set up using CHARMM-GUI (Jo et al., 2008). The MscL structure (PDB 2OAR) in the wild type and in a mutated form (L89W) were inserted in a symmetric bilayer containing 514 POPC lipids. Systems were neutralized with 150 mM concentration of NaCl. The obtained system was energy minimised and subsequently equilibrated in 6 steps following CHARMM-GUIs equilibration. Stretch-induced conformational changes in both the wild-type and mutant MscL were investigated by unrestrained simulations of 300 ns (3 simulation repeats for the WT and 2 for the L89W channel) where the bilayer plane (xy plane) pressure was changed semiisotropically to −50 bars (which corresponds to a tension of ~67.5 mN/m). The pressure in the bilayer normal (z-direction) was kept at +1 bar and the temperature at 310 K. All the atomistic systems were simulated using GROMACS 2016 (Abraham et al., 2015) with CHARMM36 force field (Lee et al., 2014) and a 2 fs time step. The Nose-Hoover thermostat (Evans and Holian, 1985) and the Parrinello-Rahman barostat (Parrinello and Rahman, 1981) were used for the stretch-induced simulations. Long-range electrostatics were managed using the particle-mesh Ewald method (Darden et al., 1993) and the LINCS algorithm was used to constrain bond lengths (Hess et al., 1997). All MD trajectories and data for this study is within the following link: http://archive.researchdata.leeds.ac.uk/777/.

QUANTIFICATION AND STATISTICAL ANALYSIS

ESEEM time-domain traces were background-corrected, apodized with a hamming window and zero-filled before Fourier transformation using the Xepr software. Fitting of ESEEM time traces was done using the MATLAB Curve Fitting Toolbox. Standard deviations for the fitted parameters were calculated and were less than 2% for all the mutants. We accounted for errors that might emanate from differences in relaxation behaviour or contribution from no-water protons between the different mutants and used an error of 5%, which has been shown to exceed all the associated experimental and fitting errors (Volkov et al., 2009). A full description can be found in the 'Method details' sections. HDX data were analyzed using PLGS (v3.0.2) and DynamX (v3.0.0) software. DynamX restriction parameters are described in the 'Method details' section. Following manual curation of the data, PAVED and Deuteros were used to identify peptides with statistically significant increases/decreases in deuterium uptake (applying 99% or 95% confidence intervals) and to prepare Wood's plots. See Table S1 for summary of HDX data. The pairwise force energy between TbMscL residue 89 and lipids (Table 1) was calculated using Gromacs' built-in command energy, in which the last 20 ns of the 300 ns simulations was specified to extract data. Standard deviation was calculated from the simulation repetitions, including the error bars presented in Figure S9B.

Structure, Volume *30*

Supplemental Information

Pocket delipidation induced by membrane tension or modification leads to a structurally analogous mechanosensitive channel state

Bolin Wang, Benjamin J. Lane, Charalampos Kapsalis, James R. Ault, Frank Sobott, Hassane El Mkami, Antonio N. Calabrese, Antreas C. Kalli, and Christos Pliotas

Supplementary Material for

Pocket delipidation induced by membrane tension or modification leads to a structurally analogous mechanosensitive channel state

Bolin Wang,[1,2†] Benjamin J. Lane,[1,2†] Charalampos Kapsalis,[3] James R. Ault,[1,4] Frank Sobott,[1,4] Hassane El Mkami,[5] Antonio N. Calabrese,[1,4] Antreas C. Kalli,[1,6] Christos Pliotas[1,2,3*]

[1]Astbury Centre for Structural Molecular Biology, University of Leeds, UK
[2]School of Biomedical Sciences, Faculty of Biological Sciences, University of Leeds, UK
[3]Biomedical Sciences Research Complex, School of Biology, University of St Andrews, UK
[4]School of Molecular and Cellular Biology, Faculty of Biological Sciences, University of Leeds, UK
[5]School of Physics and Astronomy, University of St Andrews, UK
[6]Leeds Institute of Cardiovascular and Metabolic Medicine, School of Medicine, University of Leeds, UK

[†]These authors contributed equally to this work

*Corresponding author: Christos Pliotas, c.pliotas@leeds.ac.uk

This file contains supplementary:

Figures with legends 1 to 10
Tables with legends 1 to 2

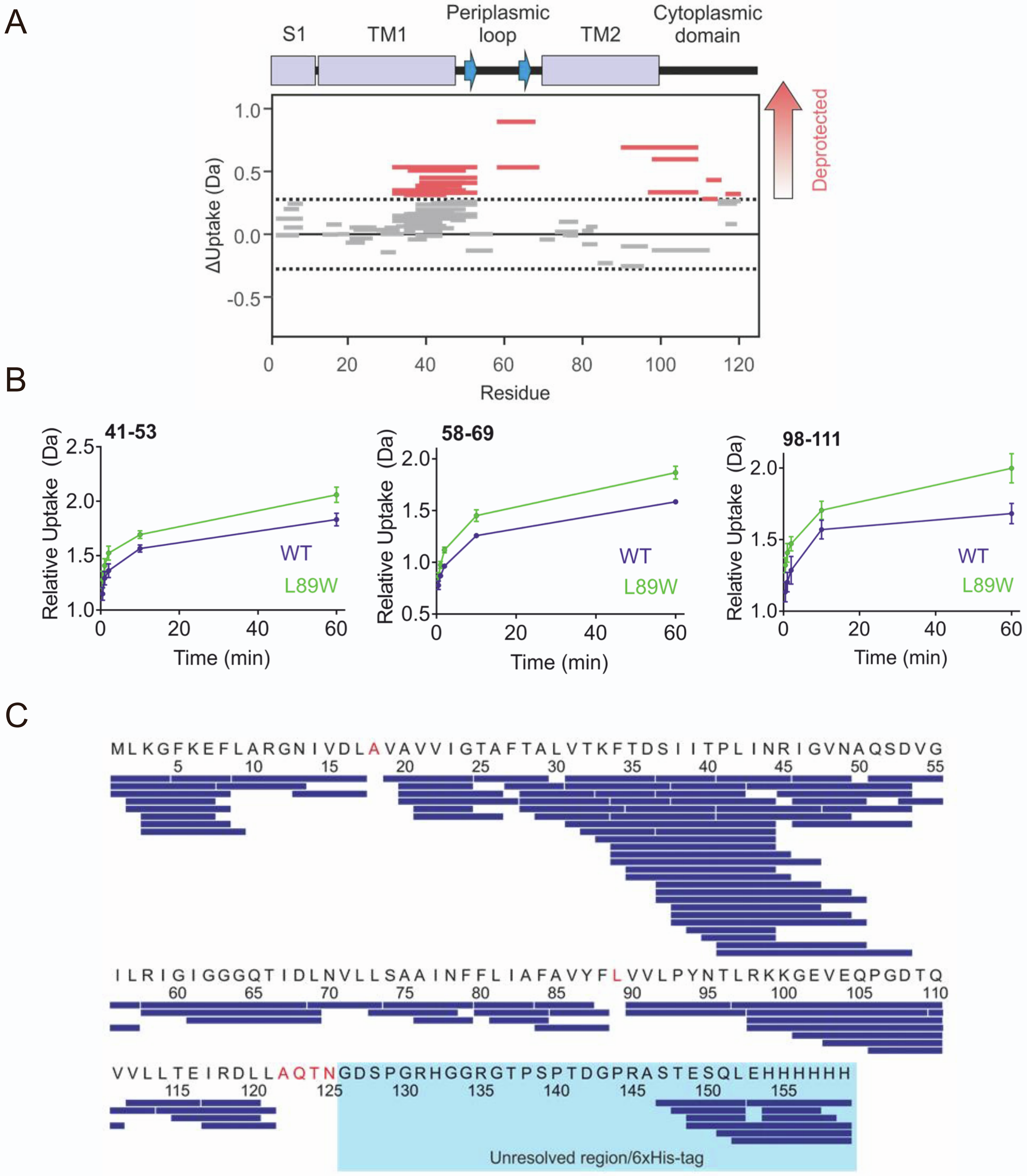

Figure S1. HDX-MS comparing WT and L89W MscL, Related to Figure 1. A. Wood's plots showing the summed differences in deuterium uptake in MscL over all five HDX timepoints, comparing wildtype MscL with L89W MscL (Wood's plots were generated using Deuteros (Lau et al., 2019). Peptides coloured in red, are deprotected from exchange in L89W MscL. No peptides were significantly protected from exchange in L89W MscL compared with wild type MscL. Peptides with no significant difference between conditions, determined using a 95% confidence interval (dotted line), are shown in grey. B. Example deuterium uptake curves for MscL WT (blue) L89W (green). The residue numbers in each peptide are indicated in the top left of each plot. C. Map showing peptides from MscL detected in the HDX-MS experiment (blue bars). Residues in red are not covered by any of the detected peptides. The region highlighted in light blue corresponds to the region of the protein that is not resolved in the x-ray structure and the 6xHis-tag.

A

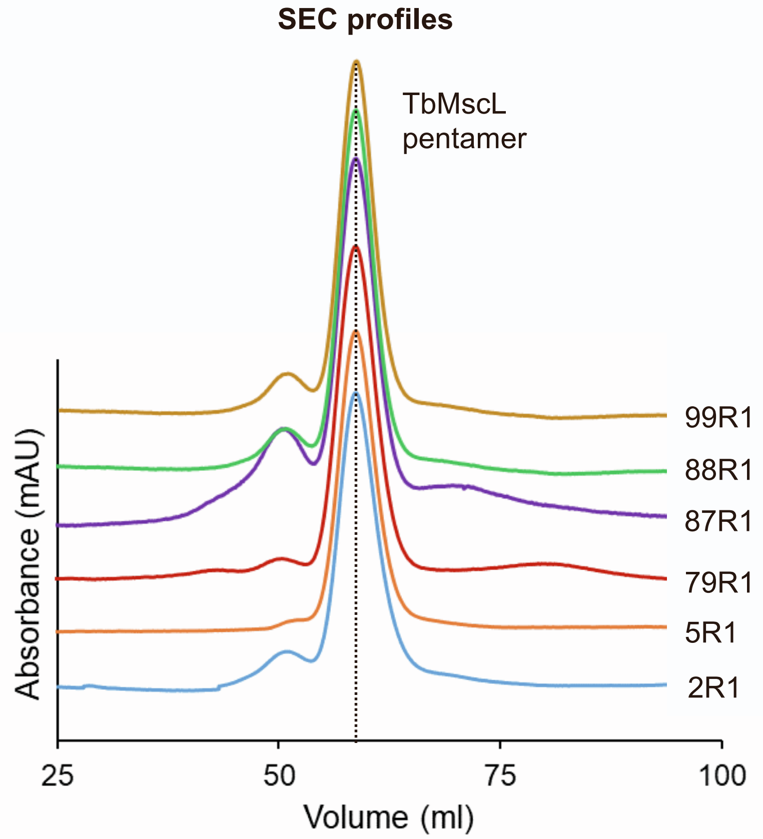

B

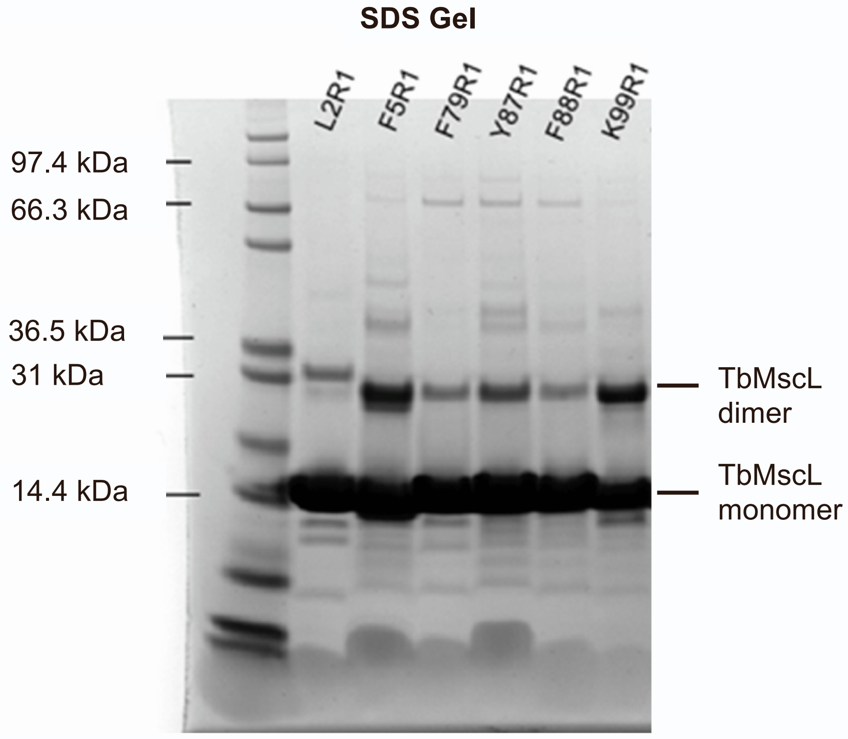

Figure S2. Purification of MscL Mutants, Related to Figure 2 and Figure 3. Sample characterisation and assessment of purified TbMscL proteins used for 3pESEEM and HDX-MS. A. Size exclusion chromatography (SEC) profiles of spin labelled TbMscL mutants representative of multiple protein domains. All purified mutants are monodisperse and in pentameric states in DDM solution, consistent with previous data (Kapsalis et al., 2019). B. SDS gel analysis of the respective mutants (SEC profile peaks). Two distinct bands appear for each mutant, which correspond to the monomeric and dimeric form(s) of the labelled proteins, under SDS denaturing conditions.

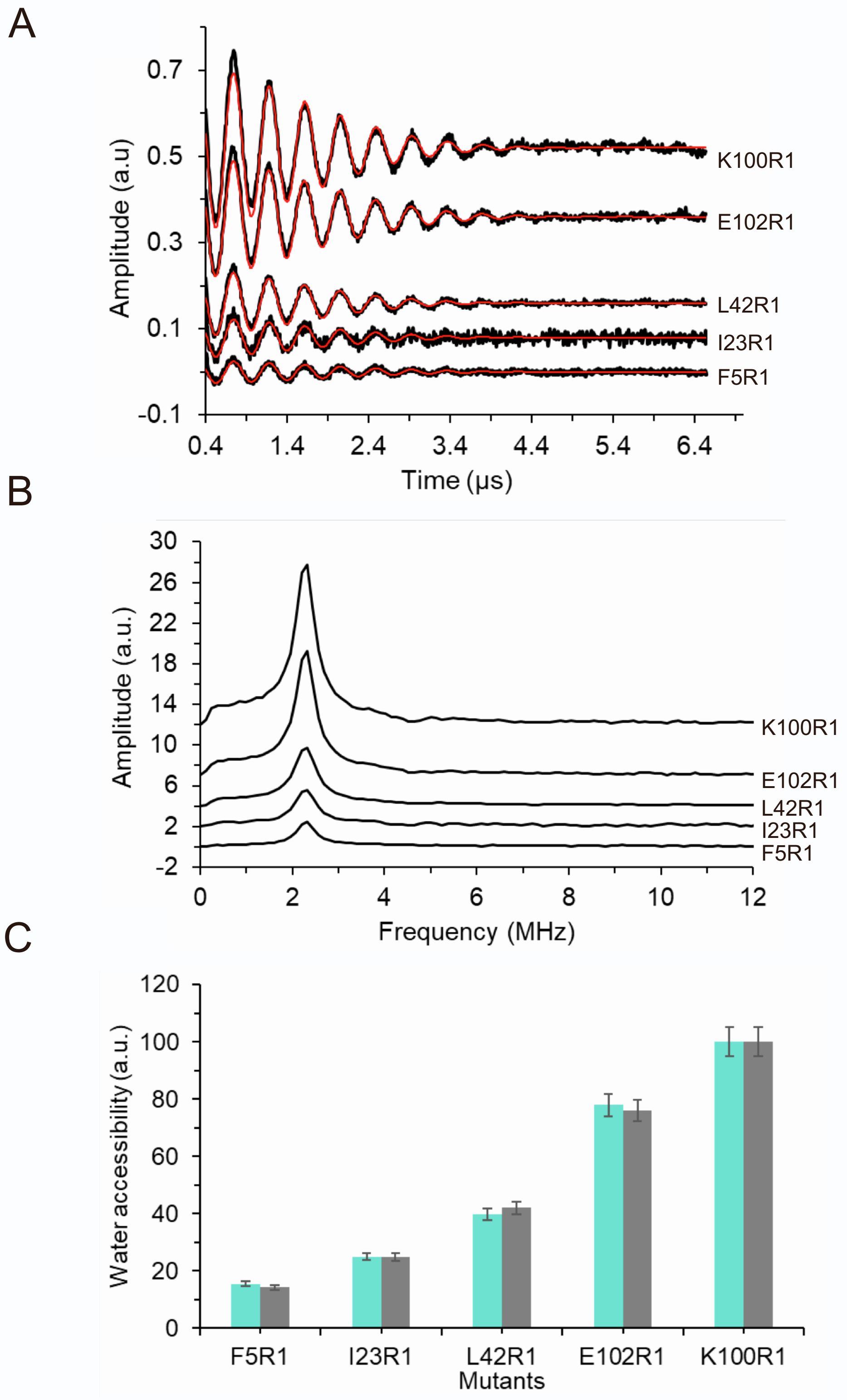

Figure S3. Comparison of Deuterium (solvent) accessibility obtained from 3pESEEM time-domain and frequency spectra, Related to Figure 2 and Figure 3. A Background-corrected time-domain 3pESEEM raw spectra (black traces) with fitting (red) of representative in respect of solvent exposure level and MscL domain coverage spin-labelled mutants. F5R1 is found on the S1, I23R1 and L42R1 on TM1, and K100R1 and E102R1 are at the interface between TM2 and the CHB. B Frequency domain spectra of 3pESEEM data of F5R1, I23R1, L42R1, K100R1, and E102R1. C Column bar charts representing solvent accessibility parameters obtained by two different analysis method approaches. For each sample, the cyan bars correspond to the solvent accessibility derived from the deuterium amplitudes in frequency domain 3pESEEM spectra and normalized to the highest accessibility corresponding to 100%. The grey bars correspond to the solvent accessibility determined from the fitting model to the time domain 3pESEEM spectra and normalized to the highest accessibility corresponding to 100%. More details on data processing in *Materials and Methods.*

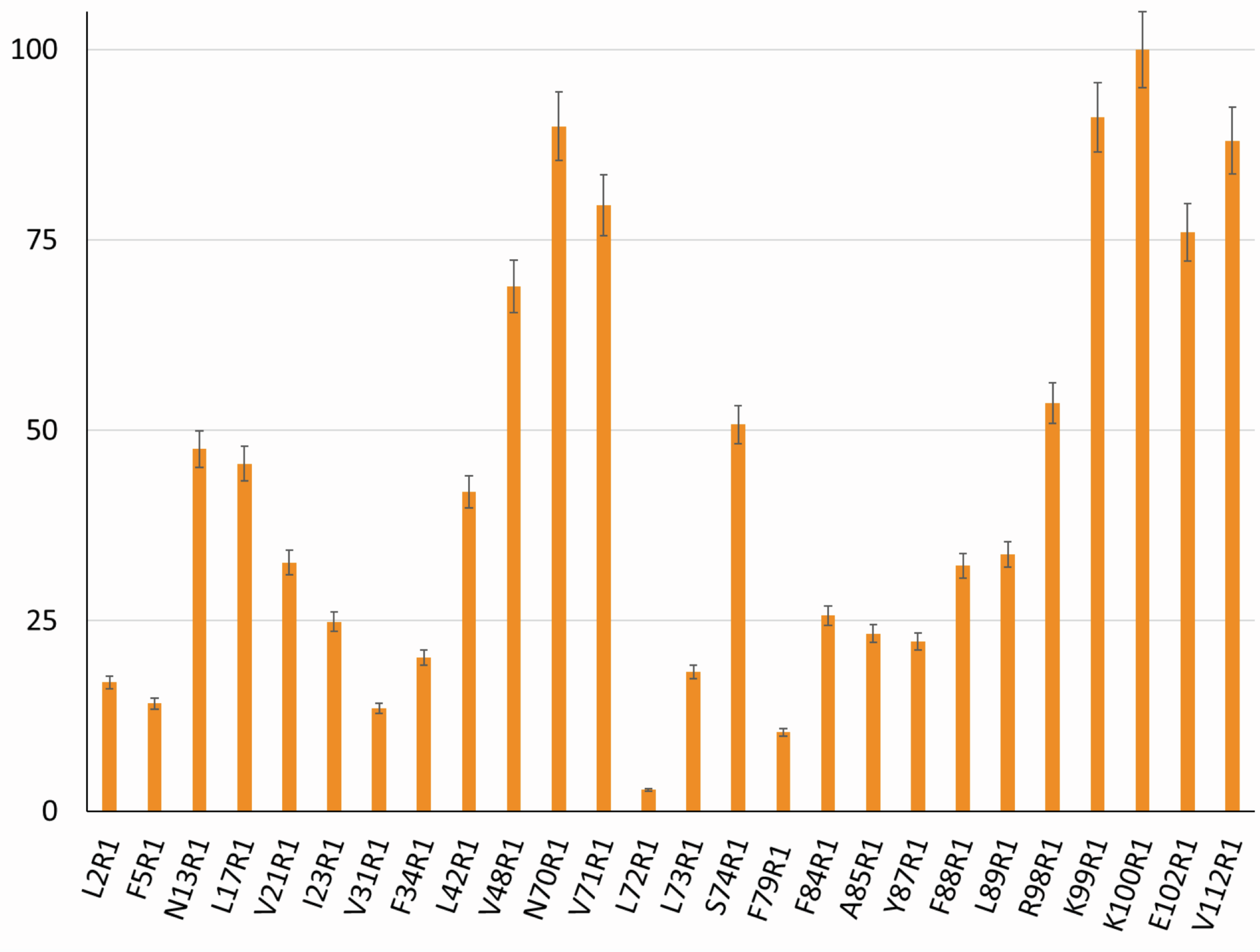

Figure S4. Solvent accessibility of spin-labelled residues, Related to Figure 2. The column bar charts represent the deuterium (or solvent) accessibility of each spin-labelled residue derived from the fitting of 3pESEEM time-domain traces, normalised on a scale between 0 and 100**.** Errors are calculated at 5% to compensate for fitting errors and differences in the relaxation times of different spin labelled residues.

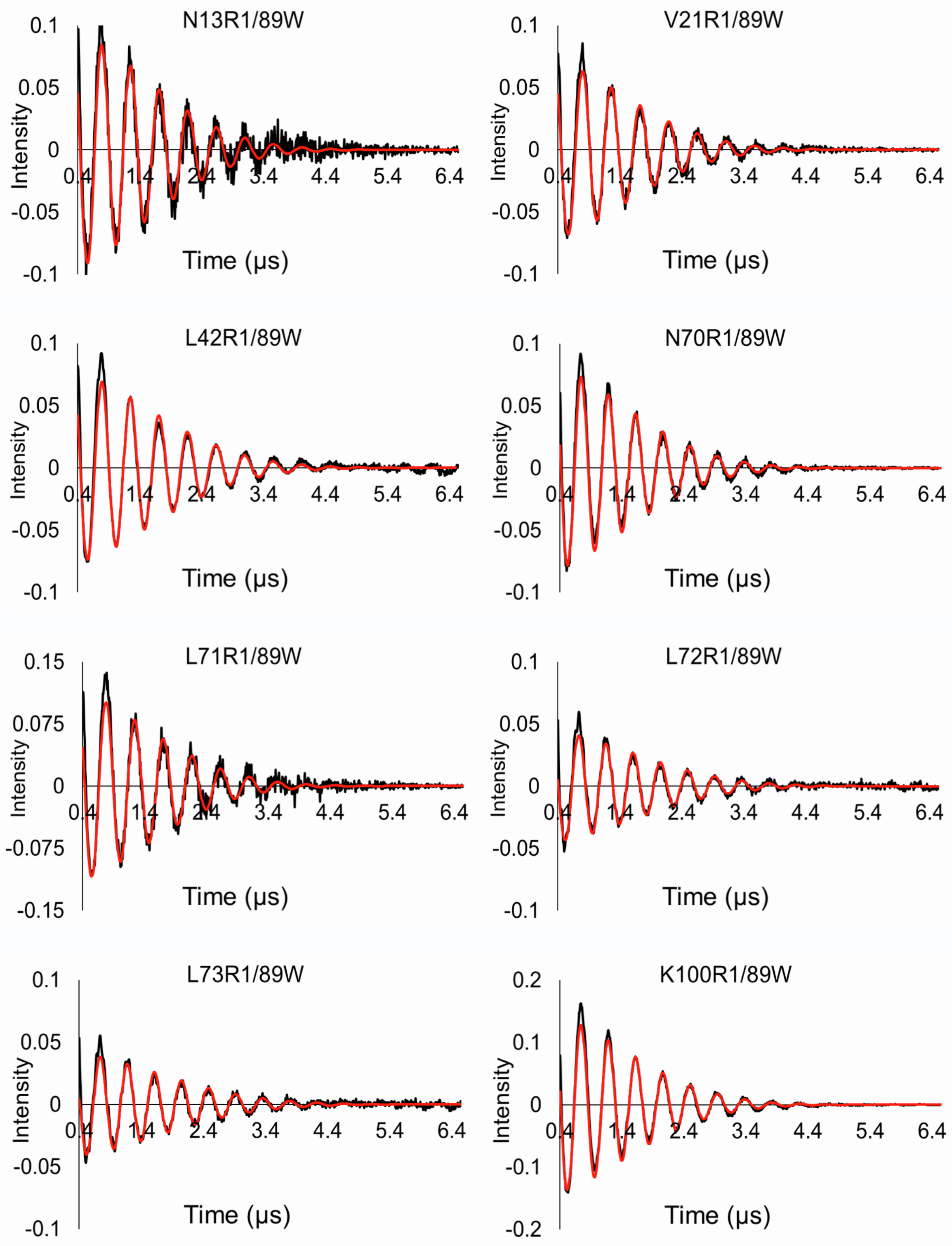

Figure S5. Time-domain 3pESEEM raw (black) and fitted (red) experimental spectra of the double TbMscL mutants N13R1/89W, V21R1/89W, L42R1/89W, N70R1/89W, L71R1/89W, L72R1/89W, L73R1/89W, and K100R1/89W, Related to Figure 3.

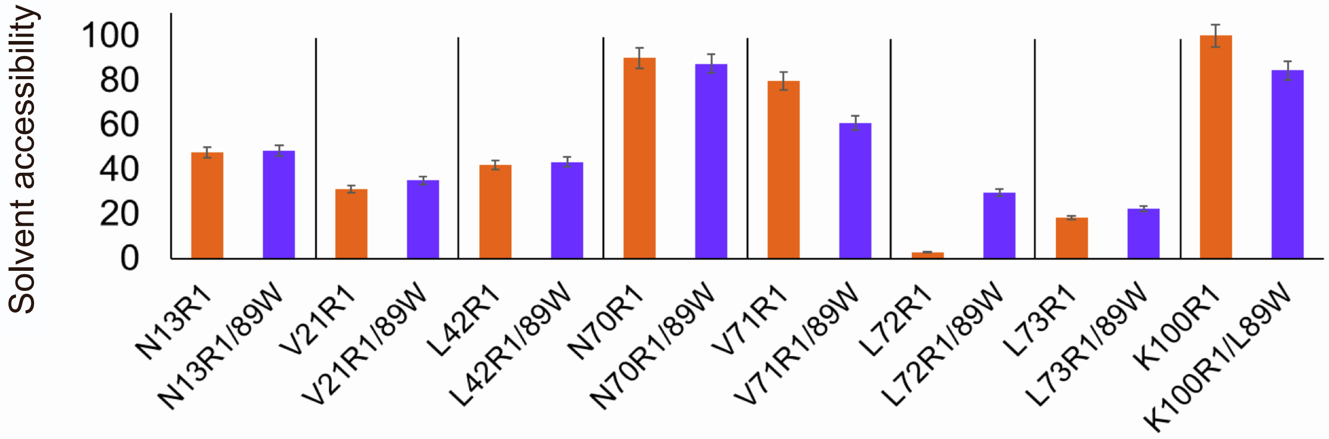

Figure S6. Column bar charts represent the Deuterium accessibility derived from the fitting of 3pESEEM time-domain traces, Related to Figure 3. Errors are calculated at 5% to compensate for the fitting error and differences in relaxation times of different spin labelled residues (*for more details see Methods*).

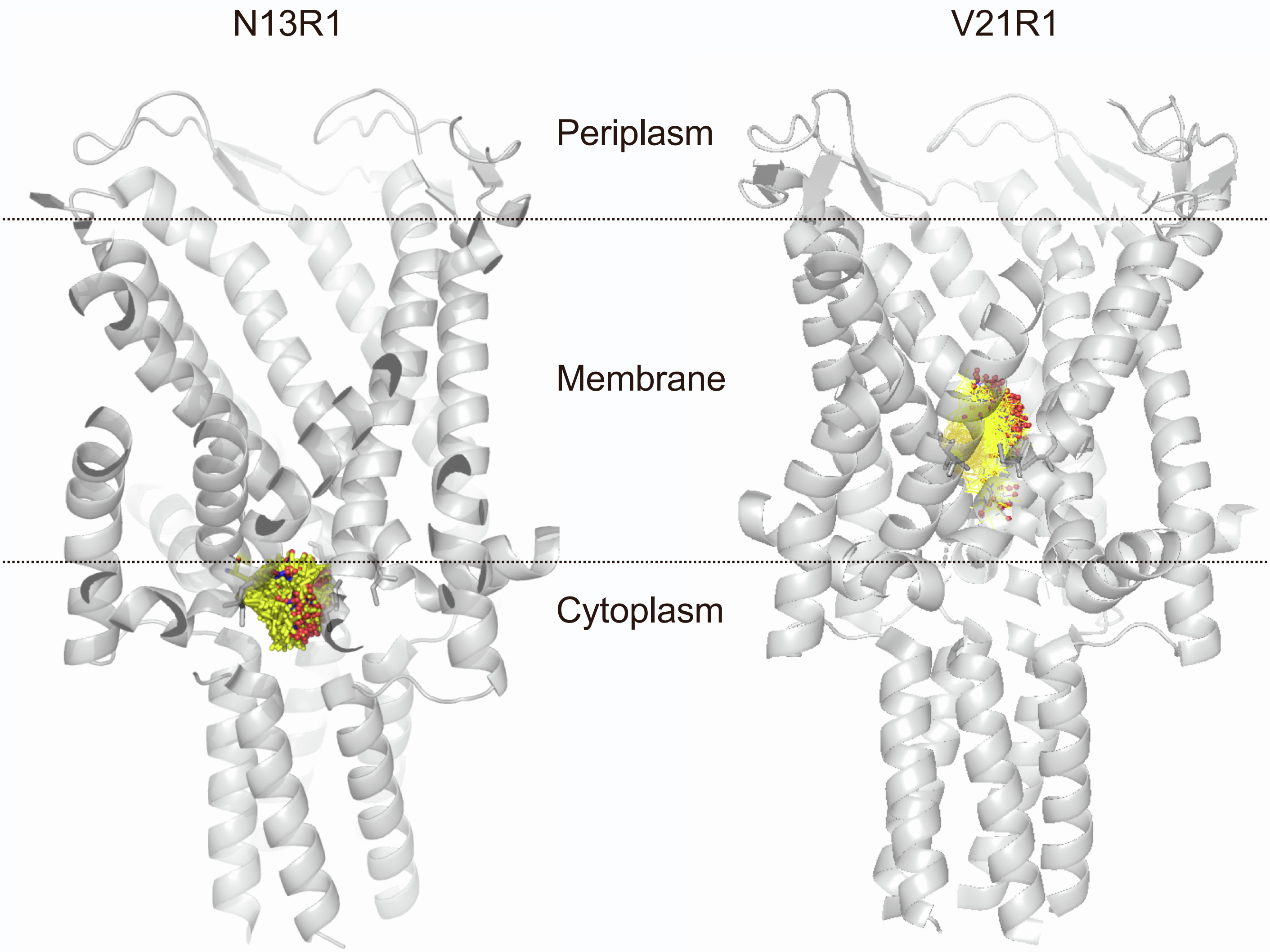

Figure S7. *In sillico* spin labelling of TbMscL (PDB 2OAR, closed state) N13 and V21, Related to Figure 3. N13R1's side chain is already solvent exposed in the closed state, due to pointing into the cytoplasm, thus unaffected in the expanded intermediate state.

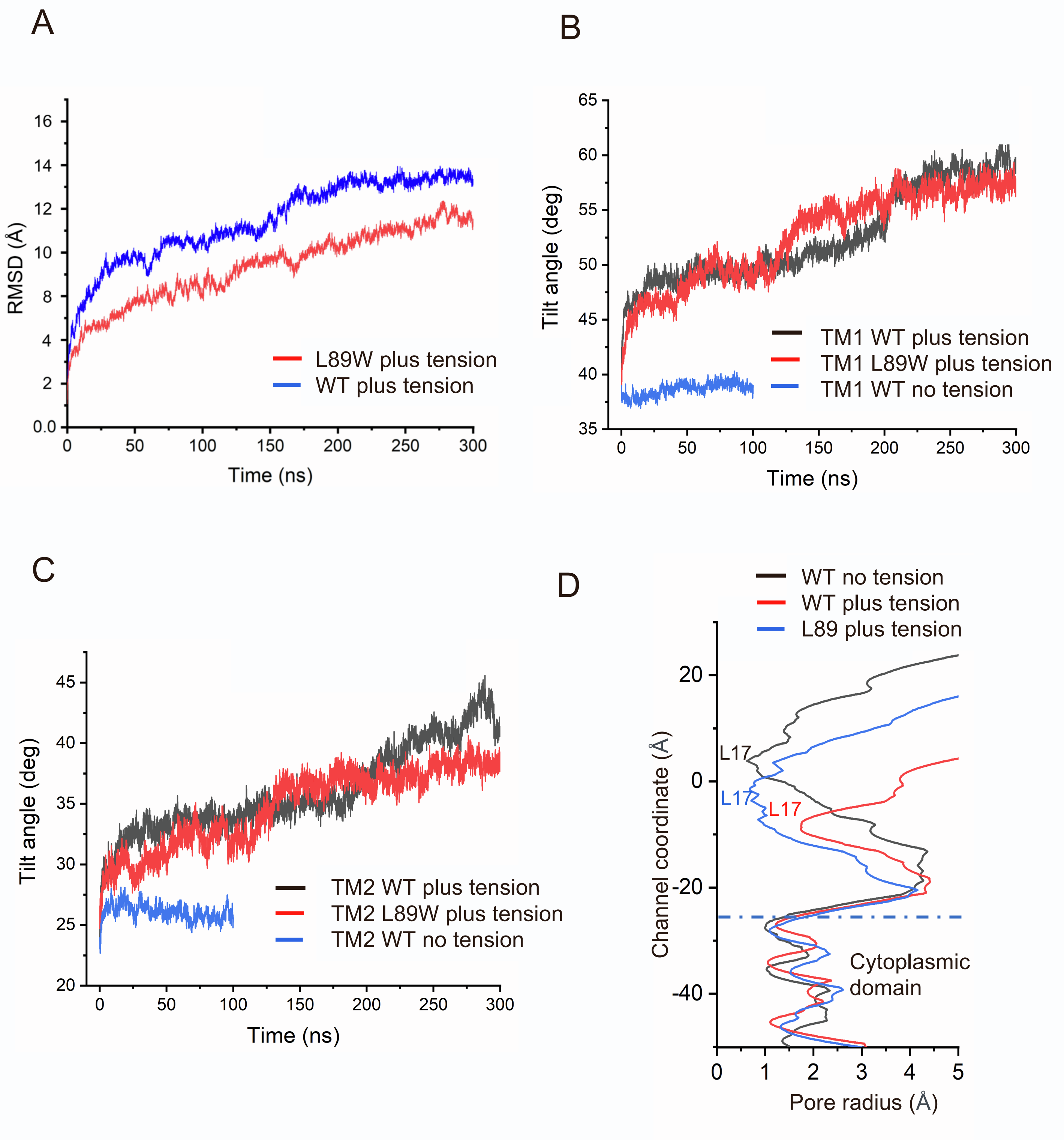

Figure S8. Comparison of WT and L89W MscL in MD simulations under no and applied membrane tension conditions, Related to Figure 4 and Figure 5. A. RMSD between WT and L89W TbMscL under applied to the x-y, parallel to the membrane plane bilayer tension. Tilting angle comparison between under tension and no tension and modified states of B. TM1 and C. TM2 with respect to the z-axis over time. D. MscL pore radius profiles analysis and comparisons using HOLE(Smart et al., 1993). Although L89W MscL undergoes similar major rearrangements to WT MscL under tension its pore remains significantly smaller and almost identical to WT closed MscL under no tension.

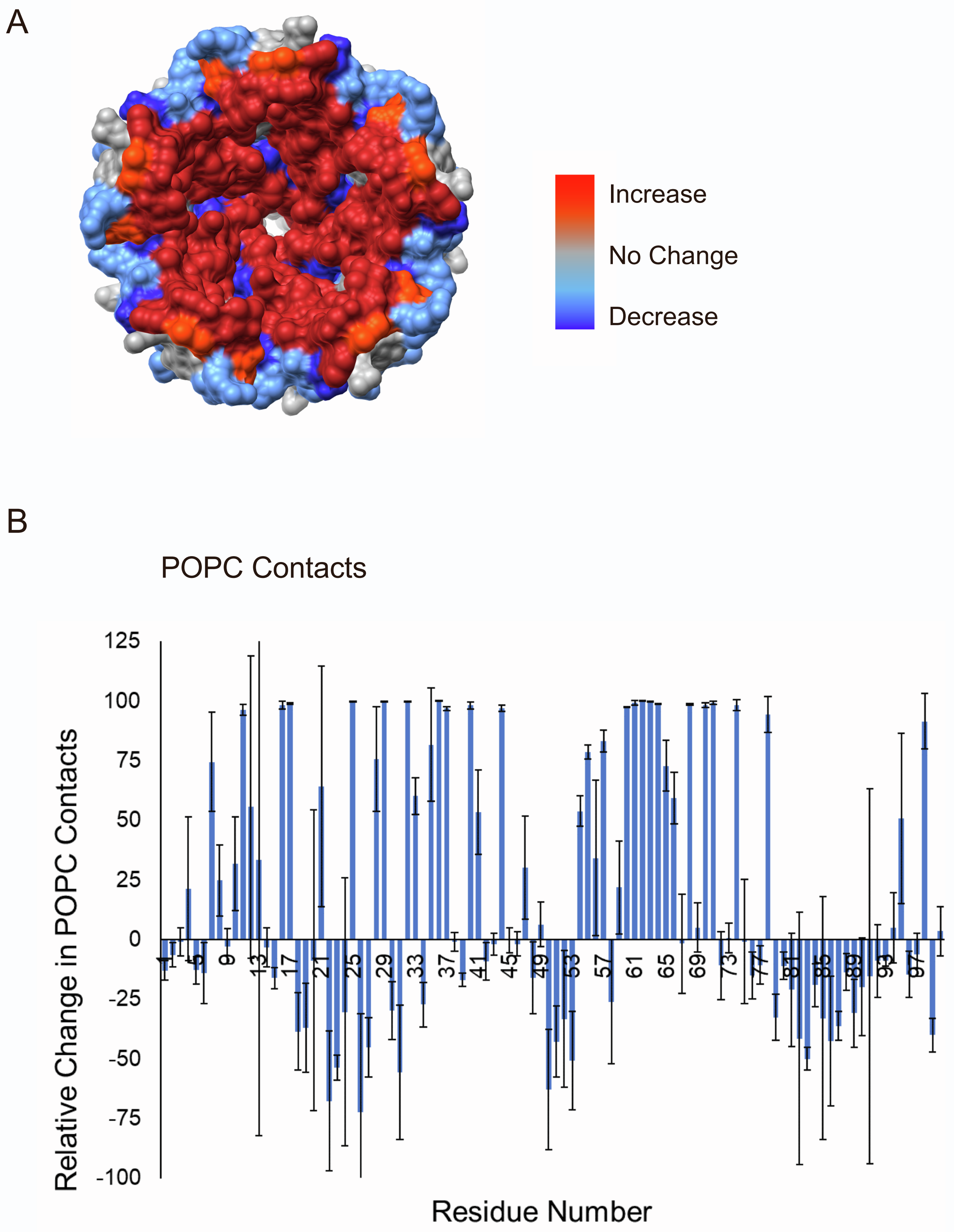

Figure S9 The effect of tension on lipid-residue contacts in MD simulations, Related to Figure 6. A. MscL top pore view showing relative changes in the number of lipid contacts following simulated tension application in the membrane during MD. The blue regions show decrease in lipid contacts, while the red regions show an increase in lipid contacts. B. relative difference between the expanded tension-activated state and the closed (no tension) state of POPC contacts with TbMscL residues.

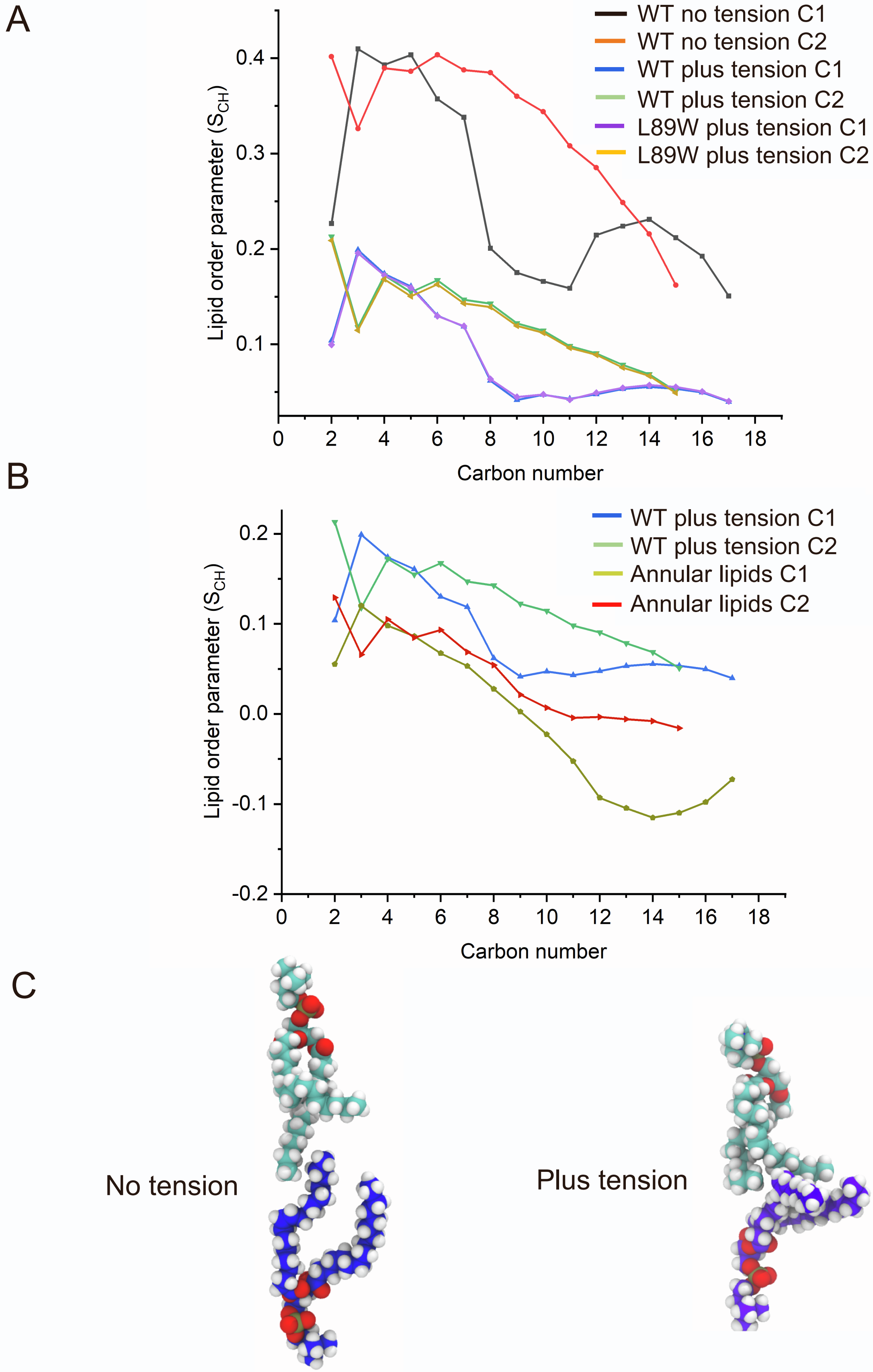

Figure S10. Comparison of the lipid order parameters, Related to Figure 6. A. Comparison for all the lipids included in the simulation of WT MscL with no tension, WT and L89W MscL under tension. Lipid chains are more horizontally oriented under tension compared to chains with no tension while in both L89W and WT MD simulations lipid chains adopt similar horizontal orientations. B. Comparison for all bilayer and annular lipids included in the WT MscL MD simulations under tension. Annular lipids are more "horizontally" oriented than bulk bilayer lipids. C. representative lipids in MD with and no tension. S_{ch} was calculated using the formula: $S_{ch} = ½ * (3 \cos^2θ – 1)$, where *θ* is the angle formed between the (C-1) and (C+1) vector and the membrane horizontal axis.

Table S1. HDX Data Summary Table, Related to Figure 1.
SD = standard deviation, CI = confidence interval

**Data Set**	**Tb MscL**	**L89W MscL**
**HDX reaction details**	50 mM potassium phosphate pH 7.4, 300 mM NaCl, 0.05% DDM	
**HDX time course (min)**	0.5, 1, 2, 10, 60	
**HDX control samples**	Maximally-labelled controls were not performed.	
**Back-exchange**	~ 30 %	
**# of Peptides**	101	101
**Sequence coverage**	83 %	83 %
**Average peptide length / Redundancy**	7.3 / 5.6	7.3 / 5.6
**Replicates (biological or technical)**	3 technical, 2 biological	3 technical, 2 biological
**Repeatability**	0.049 (average SD)	0.053 (average SD)
**Significant differences in HDX (delta HDX > X D)**	Reference	99% or 95 % CI in summed data using Deuteros

Table S2. Overall RMSD among structures generated in this study by MD (under membrane tension) and the expanded x-ray MscL structure (PDB 4Y7J), Related to Figure 4 and Figure 7.

	WT under tension (MD) (Å)	L89W under tension (MD) (Å)	Expanded x-ray structure (Å)
WT under tension (MD) (Å)	-	3.1	3.3
L89W under tension (MD) (Å)	3.1	-	3.4
